# Supplementary material for: Seroprevalence of IgG antibodies against SARS-CoV-2 among the general population and healthcare workers in India, June–July 2021: A population-based cross-sectional study
Source: PLoS Med. 2021 Dec 10;18(12):e1003877. doi: 10.1371/journal.pmed.1003877 (PMC8726494; doi:10.1371/journal.pmed.1003877)
Supplement: S1 Appendix — (DOCX) [file pmed.1003877.s002.docx]

**S1 Appendix: Acknowledgements**

We thank Indian Council of Medical Research for funding this study. Authors gratefully acknowledge the technical inputs provided by the Epidemiology and Surveillance Working Group of the ICMR COVID-19 National Task Force. The authors also thank state level survey teams for their field work and WHO India, state and district health officials, and primary healthcare staff for coordination of field operations. We thank the laboratory and data management teams at ICMR-National Institute of Epidemiology, Chennai (S1 Appendix).

**Epidemiology and Surveillance Working Group:**

Tanu Anand, Giridhara R Babu, Himanshu Chauhan, Tanzin Dikid, Raman R Gangakhedkar, Shashi Kant, Sanket Kulkarni, J P Muliyil, Ravindra Mohan Pandey, Swarup Sarkar, Naman Shah, Aakash Shrivastava, Sujeet K Singh, Sanjay Zodpey

**Laboratory and data management team:**

K Aparna, K Arul, PR Asish, M Chellakumar, D Chokkalingam, R Gunasekaran, Anusha Hindupur, S Kalaivani, K Kalaiyarasi, C Kanagasivam, N N Karthick, G Kiruthika, S Manickam, M Purushothaman, S Sarathkumar, M P Sarath Kumar, E Michaelraj, Josephine Pradhan, E B Arun Prasath, Sudha Rani, Amanda Rozario, R Sivakumar, P Gnana Soundari, K Sujeetha, P Tamilselvi, G Veeravel, Arya Vinod.

**State Survey teams**

**Assam**

Doirai Basumatary, Liladhar Boro, Jumanjyoti Sarmah, Anjan Saikia, Anu Chouhan, Dhurbajyoti Pathak, Nagen Sarmah, Tapan Hazarika, Nandeswar Hazarika, Arup Kr. Deka, Joydhon Timung, Suprotin Khatoniar, Upashna Bora, Dondodhar Gogoi, Thomas Langthasa, Thaneswar Teron, Bijoybhusan Deka, Sibsankar Chakaraboty, Pranjal Nath Ranjit Das, Bibek Saikia, Ritwik Dutta, Dipti Kangkana Kachari, Amir Sohail Khan, Mrinal Nagtey, Rimpi Konwar, Jasuda Chetia, Gunin Mili, Boidujya Rai Gogoi, Bidya Pegu, Ananta Borah, Gamuk Kuttum, Ranjali Doley, Agastin Kerketta, Santanu kakoty, Pullab Das, Prahlad Das, Paramananda Upadhaya, Krishna Khadka, Pankaj Phukan, Pranjal Das, Samir Ranjan Borah, Rituraj Buragohain, Tapan Kalita, Mrinmoy Gogoi, Amit Kr. Ray, Diganta Karmakar and Mahanta Gogoi

**Bihar**

Naveen Kumar Mandal, Kumar Gautam, Sanjeev Gupta, Ujjwal Prakash, Sahdeo Mandal, Prakash Ranjan, Saurav Kumar, Santosh Kumar, Rohit Kumar, Sashi Bhusan Awasthi, Mritunjay Kumar Pandey, Paras Kumar, Kumar Rakesh Mandal, Satish Kumar Thakur, Subhash Kumar, Manohar Kumar, Amit Lakra, Ashish Kumar, Binod Kumar, Rishikesh Kumar, Prateek Raushan, Vikas Kumar, Saurabh Krishna, Baidyanath Roy, Alok Kumar, Santosh Singh, Sakaldeep Kumar, Ajeet Kumar, Aaditya Panday, Umesh Kumar, Dhirendra Kumar, Amar Kant Singh, Sanjeet Kumar, Bhoop Dhakar, Vikash Kumar Roy, Kundan Kunal, Vikash Kumar, Kumar Abhijeet, Amrendra Kumar, Vikram Kumar Chaudhary, Mohammad Arif, Raneeta Singh, Madhu Kumari, Roushan Kumar, Parul Priya, Pooja Kumari, Manoj Kumar, B.P. Subramanya, Ujjwal Prasad Sinha, Rajeev Chandra Kumar, Ashish Tigga, Sanjay Kumar Singh, Geetika Shankar, Anand Gautam, Susheel Gautam, Rajendra Kumar, Adarsh Varghese, Anisur Rehman Bhuiyan and Kunnal Kuvalekar.

**Chhattisgarh**

Archana Nagwanshi, Sunil Kumar Pankaj, Irshad Khan, Chetan Ravishankar Raut, Kunjbihari Patel, Chandra Kishore Thakre, Pekhan Kumar Sahu, Nand Kumar Modi, Nand Kumar Sahu, Bhoopendra Thakur, Awadh Baghel, Hemant Bawthande, Dev Kumar Sahu, Rajesh Kumar Soni, Devdas Joshi and Vokesh Yadu

**Gujarat**

Dinkar Raval, SK Makwana, A.M. Kadri, Harsh Bakshi, Pranav Patel, Arthur Mcwan, Anand Santoke, Pankaj Nimawat, Shabbir Ali Dedhrotiya, YK Jani, Jitendra Patel, Hasmukh Parmar, Hardik Nakshiwala, Vaidehi Gohil, Jaydip Oza, Vikas Kokare, Mihir Rupani, Ankit Sheth, Parulben Patel, Jigneshbhai Tadvi, Priyank Gandhi, Piyushbhai Parasar, Vinodbhai Valvi, Jagdishbhai Padvi, Dhawal Patel, Divyaben Zala, Mayurbhai Vasava, Manmitbhai Solanki, Darshnaben Patel, Chetnaben Chaudhari, Aartiben Rathva, Riyaben Mistry, Nikiben Bhau, Jyotsnaben Bariya, Tejasbhai Patel, Kartikbhai Prajapati, Babita Roy, Pareshbhai Parmar, Manojbhai Bhagora, Pareshbhai Patel, Hemantbhai Kalasva, Shardaben Vankar, Divya Patel, Ravisinh Chauhan, Nimisha Patel, Misha Patel, Harsha Sadat, Puja Patel, Girish Shah, Partapsinh Taviyad, Raginiben Gosai, Krutikaben Rana, Imtiyazbhai Shaikh, Madhuben Mahera, Bhavikaben Patel, Prakashakumar Patel, Sangitaben Patel, Geetaben Patel, Pratapbhai Pagi, Bharatbhai Rana, Jinalben Patel, Archanaben Pandavi, Dilipbhai Baria, Ishavar Sinh Rathod, Sharmishtha Patel, Sunitaben Solanki, Bhavesh Vaghela, Moinuddin Mansuri, Nitesh Rathore, Purvi Nayak, Hardeep Khair, Rajendra Acharya, Vijyaben Amin, Nirmal Prajapati, V.J. Pargi, Asmitaben Kharadi, Rajubhai Patel, Komalben, Hemangini Baria, Meenaben Bamaniya, Shantaben Prajapati, Rameshbhai Patel, Imran Mansuri, Yashvantbhai Nayak, K.K. Parmar, Rahul Siroi, Krunal Darji, Mahavir Solanki, Shivani Joshi and Mahesh Gavit.

**Jammu and Kashmir**

Haseena Mir, Syed Arshad Rafiq, Iram Sabah, Misbah Ferooz Kawoosa , Abdul Aziz Lone, Ishtiyaq Ahmad Sumji, Mehvish Afzal Khan, Shaista Ismail, Anjum Asma, Shifana Ayoub, Javed Ahmad Bhat, Shafin Ashraf wani, Asima Nazir, Mohd. Ashraf Bhat, Irfana Gani, Neelofar Akram, Riyaz A Wani, Manzoor A Magray, Gulban Fayaz, Parsa Shafi, Aasim Abubakar, Aymen Deeba, Aasim Maqbool, Firdous Mushtaq Khan, Mir Waleed, Inam Zahoor, Nimrat ul Ain Banday, Kaisar Hamid Malla, Saika Parvaiz, Wajahat Nazir Shazia Akhtar, Nazir Ahmed Bhat, Mushtaq Ahmad Bhat, Jehangi Ahmad Bhat, Naseeb Singh Bali, Ferooz Ahmad, Altaf Ahmad, Abdul Rashid Dar, Farooq Ahmad Magray, Manzoor ahmad, Gh Mustafa Bhat, Feroz Ahmad Bhat.

**Jharkhand**

Asit Mansingh, Amiya Ranjan Mohanta, Anjan Kumar Bishoyee, Trilochan Bhoi, Matrujyoti Pattnaik, Santosh Kumar Sahoo, Rajesh Kumar Panda, Ashok Kumar Mahakud, Debashish Mishra, Partha Sarathi Patra, Dasrath Majhi, Santosh Kumar Behuria, Jeevan Kumar Mohanto, Biren Kumar Padhy, Chanan Kumar Majhi, Krushna Chandra Dalei, Soumya Ranjan Panda

**Karnataka**

Jawaid Akhtar, K V Thrilok Chandra, Arundathi Chandrashekar, Patil Om Prakash, Rameshchandra Reddy,N Udanesha, Sarika Jain Agrawal, H P Arundathi Das, Ranganath R, Vivekanand Reddy,Mahesh Kumar, Bilagumba Ramaiah Venkateshaiah, Prathibha Javare Gowda, Ambaraya Rudrawadi, Basavaraj Biradar, Anand PrakashK R Nischith, G Hamsaveni, Satish Ghatage, R S Sreedhar, Mishba Hani, Anil Talikoti, N T Nagraja, , Pranesh Katti, Ratnasheela, Qayyum, Sangamesh,Shridhar, Amrut, Sankar Gouda, Maruthi Kamble, Anitha Jadhav, Deepak Patil, Amaraya Rudrawadi, S K Ravikanti,G Abhay Kumar, Ratnakar Toran, Suresh Doddamani, Suresh Mekin ,Deepak Patil, Siddaramappa Patil, Avinash Khaske, Abdul Jabbar, Shashidhar Patanaik, Amaresh Kolur, Asha,Shobha Nagesh, Ramya R, Babu Mahendra, Kusuma S, Roopashree, Malathi Y S, Bharath,Latha, Sudha,Shivakumar, G, Srinivas, Sadiqulla Sharif, Jagadeesh, Dilip,Vijay, Mallikarjunappa, Kavitha, Sudha, Premasudha, Chandrashekhar Kambalimat, Girish, Jayashimha, Venkatesh, Ananda Prakash, Shree Harsha, Vijayalakshmi N, Somashekharayya S Hiremath, K Jaya Ganesh, H S Charan Raj Rao, T.N.Basavaraju, B.Sampath, T.G.Gangadharaiah, H T Mangalagowri, Nagaraja P C, Ravikumara M T, Srikantha Y G, Hariprashanth R, Praveen Pujar, Bheema Zakeer Hussain, Namewar Hanmanth, Kokila Manickam, Gowri K, Shadul Fakirsab Sayyad, Lal Kumar R, Ullera Ashoka, A N Sunil, Umar Farooque M Dalawai, Manjunatha M, Venkatesh A Millanatti, B.Dinesh Kumar, Narasimharaju N, Deepak, Sundara Murthi.R.

**Kerala**

Aravind Krishnan, Manoj M, Anumol Raju, Anupranam M P, Arima A R, Soniya Joseph, Shilna A, Vishnu Raj, Prakash Jaison V, Venoth V S, Gladson J, Ganga G S

**Maharashtra**

Ankita Dahiwade, Shabana Khan, Swati Salunkhe, Seema Nair, Shudhanshu Rane, Kanad Patil, Suresh Kumar, Savita Bahekar, Rakesh Gaikwad, Pravin Dandge, Namrata Hajari, Anil Rathod, Shaikh Shahrukh, Dinesh Jadhav, Pooja Pawara, Sonal Gawai, Priyanka Bawane, Vinod Pethkar, Dhiraj Panpatil, Ajit Buchude, Sourabh Tidake, Sagar Rokade, Bhuddhabhushan Bhadke, Kapil Khandagale, Chaitanya Deshmukh, Adinath Rokde, Suraj Rakhunde, Prathamesh Chavan, Pramod Jamale, Rahul Arke, Vivek Yengade, Tejas Phale, Amarkumar Ambade, N. Ramaswami, Satish Pawar, Archana Patil, Pradeep Awate, Jyoti Gurav, Abhijeet Raut, Deepak Mugalikar, Vipin Itankar, Rajendra Bhosale, Ravindra Jagtap, Abhijeet Chaudhari, Rajesh Deshmukh, Nagurao S. Chavan, Bhimashankar Jamadar, Nilkanth Bhosikar, Balaji Shinde, Suryankant Sable, Radhakrishna Pawar, Sunil Pokhrna, Sandip Sangle, Sanjay Salunkhe, Milind Pore, Balasaheb Nagargoje, Shankararo Deshmukh, Sandip Bharaswadkar, Aniruddha Kadu, Rajiv Kumar, Hemant Kharnare, Suhasini Kadhe, Avinash Jadhav, Chetan Khade, Amol Gaikwad, Virendra Wankhede, Prakash Nandapurkar, Jyoti Salve

**Madhya Pradesh**

M.P. Sharma, Shivendra Mishra, Mahavir Khandelwal, Sunita Parmar, Devendra Gothwal, Manish Sharma, Seema Jaiswal, B.K. Tiwari, Arvind Verma, Ajay Goel, Purushottam Patel Ganesh Damor, , Bhagwansingh Patil, Ramswaroop Uikey, , Akanksha Kushram, Sandip Sharma, Himmat Singh, Yogendra Mourya, Prahlad Soni, Pushpendra Rajput, Priyanka Birha, Monu Sen, Rekha Prajapati, Priyanka Singore, Lipi Jain, Ashok Solanki, Kalpana Patel, Bhagat Dhurvey, Rahul Choubey, Pehal Singh Tekam, Rajendra Mehra, Ramcharit Rakesh Tiwari, Prathinesh Parouha, Rahul Belawi, Yagendra Kumar, Amit Raikwar and Twinkle Dewaker

**Odisha**

Asit Mansingh, Shakti Ranjan Barik, Siba Prasad Mallik, Prashant Majhi, Deepak Sahoo, ParthaSarathi Patra, A.K. Bishoyee, Hitesh Kumar Jain, Indurani Sagar, Santosh Kumar Sahoo, Rajesh Panda, Santosh Kumar Beuria, Sidhartha Kar, Soumya Panda, K. Sahu, Matrujyoti Pattnaik, Dasarath Majhi, Biren Kumar Padhi, Padmamohan Pradhan and Arun Padhi.

**Rajasthan**

Rajkumar Kalunda, Rajneesh Kumar, Anil Purohit, Chet Ram Meena, Pankaj Kumar, Trilok Kumar, Bhanwar Manohar, Mohit Yadav, Jograj Singh, Ayub Khan, Ram Nareshani

**Tamil Nadu**

Y. John Arokyadoss, P. Kumaravel, A. Vasudevan, Magesh Kumar, J. Chitra, Santhana Kumar, Sadham Hussain, Kuppusamy Chandrabalu, Chandra Kumar, Selvam Suresh, Nandha Kumar, Dhanagopal Rajmohan, G, Thanappan Selvendran, Suryanarayanan Santhosh, Annadurai Arjun, N. Kumaravel, Vinoth

**Telangana**

R. Ananthan, Dudekula Anwar Basha, Blessy Prabhu Priyanka Salavadi, ChandraKumar Dolla, S. Devindra, Gargi Meur, Indrapal.I. Meshram, P Ravindranadh, Paras Sharma, P. Raghavendra, Santosh Kumar Banjara, CVHS Saia ram, J.Srinivasa Rao, F. Sylvia, , MV Surekha, N.Samarasimha Reddy, Tata Aruna Kumari, V.Aruna, Ch. Anitha, N. Anjaiah, G.Bhavani, Ch. Bujji, Hrusikesh Panda, G. Hari Babu, S.V.J.Mohan Naidu, G.Neeraja, B.V. Nancharamma, D.Narasimhulu, Sk.Nasarvali, S.P.V. Prasad, B. Praveen, R.Raghunadh Babu, R.Rajyalaxmi, G.Venkat Raji Reddy, J.Raju Naik, V.Ravi, Ch.Sai Babu, M. Srinivas, G.L.Stephen, K. Sree Ramakrishna, PV. Sunu, P. Sreenu, D.Swaroopa, E. Sheela, M.Srinivas, P.Sathaiah, M.Suresh, P.Sriram, K.Sridhar, B. Srikant, R.Satyanarayanan, V.Sai Santosh, G.Tulasi, T.Usha Rani, K.Venkata Ramana, D.Vasundhara, and G. Vijaya Lakshmi.

**Uttar Pradesh- West**

Prabhat kumar, Dilip Singh, Naresh Dhakar, Rahul Kumar, Akash Yadav, Arpita Chaturvedi, Swati Singh, Brijesh Maurya, Manisha Dhakar, Narendra Singh Yadav, Sheena Singh, Renu Kanwar, Sonu Yadav, Rahul Yadav, Mewa kumar, Himalaya Kumar, Raju Deen Dayal, Balijeet Sodhi, Rajesh Jain, Shivanka Gaur, Akhil Tondon, Deepak Ohari, Saubhagya Prakash, Amit kumar, Haridutt Nemi

**Uttar Pradesh- Central/Himachal Pradesh & Uttarakhand**

Dechen Yangdol, Upendra Singh, Amit Yadav, Mohit Tiwari, Gopal Prasad, Sapna Yadav, Basudev Singh, Deepak Babu, Rahul Kumar, Chakrapani Katara, Chandra Pratap Singh, Simran Kaur Bhojwani, Manish Kumar, P. Vedival, Ranjan Karmakar, Vivek Kumar, Rahul Gond, Prabhat Kumar, Hariom Kushwah, Gani Aftridi, Nistha Verma, Veer Vishal, Rakesh Sharma, Uday Singh, Saurav Yadav, Navneet Rajput, Satya Prakash, Sunny Sharma, Santosh Kushwah, Akhalesh Yadav, Papai Das, Mahavir Singh Chaudhary, Prabha Shakya, Gulshan Sahu, Kumari Dipika, Sushil Chander, Manoj Sharma, Satyvrat Vaidya, Archana Srivastava, Shishir Puri, Vishal Agnihotri, Ashish Dixit, Ashutosh Kumar, Jairam Singh, Lalit Kumar, Tanmay Kakkar, Misaam Abbas, Akhileshwar Singh, Ashish Nautiyal, Manoj Kumar, Sushil Kumar, Shailendra Barthwal, Prakash Tapriyal, Subodh Kumar, Sanjay Pandey, Namita Puri, Arti Behal, V.C Kala, Ghambir Taliyan, Gaurav Raturi, Rashi Ranjan Kukreti, Pankaj Juyal, Kuldeep Kumar, Ashok Kumar, Dhani Ram, Prem Kumar, Loknath Sharma, Suraj Mani, Raj Kumari, Jitendra Kumar, Kiran Chauhan, Prakash Tapriyal, Vineet Kumar Shukla, J.S. Rawat, Himanshu Sharma, Madhu Kumari, Rajesh Mourya, R.S. Yadav, Surinder Singh, Raju Kumavat, Sandeep Patil, Pradhumn Katara, Namrata Soni, Prashant Upadhyaya, Praveen Pachauri, Ajay Rawat, Sanjay Chopra, Jyoti Mishra, Mohammed Husain, Devi Lal

**Uttar Pradesh- East**

Amit Mohan Prasad, Shruti Pandey, Markandey Shahi, Amit S Bansal, Atul Kumar Singhal, Sushil Kumar, Radhey Shyam Kesari, Ajay Pratap Singh, Vinay Dange, Ghanshyam Singh, Shri Prakash Agrawal, Shyam Narayan Dubey, Birendra Panchal, Vishal Yadav, Mukesh Kumar Mishra, Ravi Shankar Singh, Kamlesh Sah, Sonal Rajput, Sushil Pal, Ravi Nishad, Rohit Baghel, Punit Kumar, Abhishek Kumar Mishra, Avdhesh Kumar, Anugunj Chaudhary, Pawan Kumar, Surabhi Kushwaha, Mohammed Afroz, Lal Chand, Nisha Yadav, Anil Kushwaha, Deepak Kumar, Vipul Kumar, Kiran Kumari, Akash Kushwaha, Vinod Kumar, Ram Poojan Yadav and Santosh Kumar.

**West Bengal**

Falguni Debnath, Agniva Majumder, Gargi Dutta Bhattacharyya, Subrata Biswas, Ajay Chakraborty, Jayesh Mehta, Bandita Sengupta, Abhijit Dey, Arup Chakrabortty, Subhendu Kumar Ray, Shourav Tarak, Subhadip Bhunia, Debasish Roy, Shyamal Soren, Jagannath Sarkar, Somnath Mukherjee, Prakash Chandra Mridha, Girish Chandra Bera, Bibhas Roy, Santanu Sahu, Atrayee Chakraborty, Rabiul Islam Gayen, Dilip Biswas, Samudra Sengupta, Barnaman Tudu, Tanmoy Kumar Ghosh, Ananya Biswas, Poulami Sen, Sonali Das, Tridib Das, Tarun Khatua, Paritosh Mondal, Saptarshi Bannerjee, Amit Baran Barat, Arindam Sarkar, Soumen Jana, Joyeeta Bhattacharyya, Medhavi Manish, Biswajit Namasharma, Chandan Ghosh, Debarati Chakraborty, Kunal Maiti, Milan Barman, Pintu Manik, Purnima Roy, Rajani Kurmi, Rocky Ansari, Sanglap Maity, Somobrota Naskar, Sourav Pradhan, Bishakha Pramanik, Dipannita Sardar, Sujit Kumar Shreshta, Arpita Das, Ujjal Maitra, Nawaid Ali, Chandan Ghosh, Susanta Bera, Sk. Monirul Jaman, Dev Kumar Dolai, Purnima Das, Wasim Reza, Shrikant Shankar Gawali and Rajesh Das.
