# Supplementary material for: Seroprevalence of IgG antibodies against SARS-CoV-2 among the general population and healthcare workers in India, June–July 2021: A population-based cross-sectional study
Source: PLoS Med. 2021 Dec 10;18(12):e1003877. doi: 10.1371/journal.pmed.1003877 (PMC8726494; doi:10.1371/journal.pmed.1003877)
Supplement: S1 Protocol — (PDF) [file pmed.1003877.s003.pdf]

Population-based serosurvey to estimate prevalence of SARS-CoV-2 antibodies in India: Fourth survey, June - July 2021

India is witnessing a second wave of SARS-CoV-2 infections from February 2021, spread across majority of states of India (1). As of May 29, 2021, India had reported around 25 million cases and more than 3,00,000 deaths (2). The first round of national serosurveillance was conducted from May 11, 2020, to June 4, 2020, among adults in randomly-sampled 700 clusters selected from 70 districts of 21 Indian states. The population-weighted seroprevalence after adjusting for test performance was 0.73% (95% CI: 0.34-1.13). The second round of nationwide serosurvey was conducted between 17 August and 22 September 2020 among individuals aged 10 years and above in the same clusters from 70 districts of 21 states (3). The findings indicated an overall seroprevalence of around 7%, with a ten-fold rise in adult prevalence between May and August 2020. It also indicated a substantial transmission in rural areas with the seroprevalence continuing to be higher in urban slum and non-slum areas (4).

ICMR conducted the third population-based serosurvey among individuals aged 10 years and above and health care workers between Dec 18, 2020 and Jan 6, 2021 in the same 700 villages or wards from 70 districts in 21 states selected for the first and second serosurveys. In the third serosurvey, prevalence was estimated based on the presence of both IgG antibodies against SARS-CoV-2 S1 protein of the receptor binding domain (S1-RBD) and nucleocapsid (N) protein of SARS-CoV-2 while in second serosurvey prevalence was based on IgG antibodies nucleocapsid (N) protein of SARS-CoV-2. The weighted and assay characteristic adjusted seroprevalence among general population was 24.1 (95%CI: 23.0% to 25.3%) The survey indicated that three fourth of the population were still susceptible and a higher sero-prevalence of SARS-CoV-2 infection in urban areas compared to the rural areas (5). Apart from the nationwide serosurveys, several cities and states conducted serosurveys. Metropolitan cities like Delhi, Mumbai, Pune, Chennai, Ahmedabad, Hyderabad have reported seroprevalence ranging between 17.6% and 56% at different timepoints (5).

India started COVID-19 vaccination with BBV152 vaccine (Covaxin; Bharat Biotech) and ChAdOx1 nCoV-19 vaccine (COVID-19 Vaccine AstraZeneca) since January 16, 2021 under Emergency Use Authorization for the health care workers. The vaccination strategy was expanded for frontline workers in February 1, 2021 for frontline workers. In Phase 2 individuals above 60 years old and above 45 years old with specified comorbidities

became eligible for vaccination from March 1, 2021 followed by Phase 3 for all above 45 years of age from April 1, 2021. And finally Phase 4 for all above 18 years of age began on May 1, 2021 (6). Around 44 million individuals have received two doses while 160 million have received at least one dose of vaccine as on May 29, 2021 (7).

Population-based sero-epidemiological studies measure the presence of antibodies against SARS-CoV-2 (natural and vaccine induced) in different age groups. Repeated cross sectional serosurveys conducted in the same geographical location provide estimates to monitor trends over a period of and provide information valuable for the public health decision makers to design and revise containment strategies (8). The earlier serosurveys were conducted among individuals aged 10 years and above and seroprevalence among children below 10 years is still unknown. SARS-CoV-2 poses a high occupational risk to healthcare workers who are at the forefront of managing COVID-19 cases in hospital settings. The third serosurvey indicated seroprevalence around 25% among the HCWs. Also, the HCWs were prioritised for COVID-19 vaccination in the first phase of vaccine rollout in India owing to higher risk of exposure to infected persons.

In this context, it is proposed to conduct fourth round of serosurveillance to determine the prevalence of SARS-CoV-2 antibodies in the community and health care workers as per change in epidemiology and public health response to the epidemic in the country.

The objectives of the fourth survey are:

1. Estimate the prevalence of SARS-COV-2 antibodies among (a) children aged 6-17 y and (b) adults in the general population, at the national level
2. Estimate the prevalence of SARS-COV-2 antibodies among healthcare workers at the national level

Methods

Design

Serial cross-sectional survey

Study population

A] General Population

Inclusion criteria: Usual resident, age ≥6 years, available at the time of the household visit and willing to participate

Exclusion criteria: Age < 6 years; locked household; guest/visitor; not willing to participate

B] Health care workers

Inclusion criteria: Doctors, nurses, paramedical/laboratory staff, employed at the district hospital and sub-district (taluk/CHC) levels, available at the time of hospital visit and willing to participate

Exclusion criteria: Administrative and field staff of hospital; not willing to participate

Sample size

A] General population

We plan to enrol 28,000 individuals (19,600 individuals in the age group > =18 years, 5600 children in the age group of 10-17years and 2,800 children in the age group of 6-9 years) across the country by allocating a minimum of 400 individuals to each of the 70 districts that were included in the earlier survey. We have divided the overall sample size of 28,000 in the above-mentioned age groups as per age structure of the population (Census 2011).

The sample size assuming a seroprevalence of 30%, with 10% relative precision, for 95% CI and design effect of 2 would be 1792 for children in the 6-9 age group. The required sample size is sufficient to estimate prevalence for each age group. However, in order to have a sufficiently large sample for a national level survey, we will keep the same overall sample size for the fourth serosurvey.

B] Health care workers

We will enrol 7,000 health care workers across the country as done in the third survey.

Sampling method

A] General population

We will select 10 clusters (villages in rural areas and wards in urban areas) for each of the district by Probability proportional to size sampling. In each of the selected village/ward, we will select four random locations. In each location, the survey team will choose a random start household and visit consecutive households until 7 individuals from age-group >=18 years, 2 children in the age group of 10-17 years and 1 child in the

age group of 6-9 years are enrolled. All household members, 6 years and older, will be included. Within each cluster, 40 individuals (28 individuals from >=18 years, 8 children in the age group of 10-17 years and 4 children in the age group of 6-9 years) will be included for the survey. Individuals with history of laboratory confirmed SARS-CoV-2 infection by RT-PCR and/or rapid antigen test will not be excluded. SARS-CoV-2 cases in home isolation will not be included in the survey.

B] Health care workers

We will select 100 healthcare workers from each of the 70 districts selected for the general population survey. We will conduct the survey for the HCWs in the district hospital and sub-district (taluk/CHC) levels. We will consecutively enrol the willing HCWs (Doctors, nurses, para medical staff and lab staff) working in the district hospital of the selected district. If we are unable to enrol 100 HCWs in the district hospital, then we will select sub-district (taluk/CHC) hospital for further enrollment till 100 HCWs are enrolled.

Study procedures

The study team will visit the selected households and brief them about the survey objectives and process involved. After obtaining consent, information on basic demographic details, exposure history to laboratory-confirmed COVID-19 cases, symptoms suggestive of COVID-19 since March 2020, clinical history, COVID-19 testing and COVID-19 vaccination details will be recorded. All data will be entered in an Open Data Kit (ODK) application on mobile phones by the survey teams.

Trained phlebotomists in each survey team will collect 3 ml of venous blood from each participant. The serum will be separated after centrifugation in a local health facility and transported to the ICMR-NIE laboratory in Chennai.

Study duration

Two months including data collection and analysis.

Laboratory procedures

All serum samples will be tested for presence of IgG antibodies against SARS-CoV-2 on the Advia Centaur Immunoassay system using the Siemens SARS-CoV-2 IgG Assay (Siemens Healthineers India, Mumbai) and Abbott Architect i2000SR automated

analyzer using the Abbott SARS-CoV-2 IgG assay (Abbott Park, IL, USA). The Siemens assay detects IgG antibodies against S1 protein of the receptor binding domain (S1-RBD) whereas the Abbott assay detects IgG antibodies against nucleocapsid (N) protein of SARS-CoV-2. The sensitivity and specificity of the Siemens IgG assay is 100% and 99.90% respectively (9), while the Abbott IgG assay has a sensitivity of 100% and specificity of 99.6% (9).

Testing procedures will be followed as per the manufacturer's instructions. Sera with indeterminate results will be repeat tested. For quality assurance, one percent of negative sera will be repeat tested. CLIA testing will be done at ICMR-NIE laboratory.

Analysis plan

A] General population

We will calculate the frequency of characteristics of survey participants. The reported occupations will be categorised into high and low risk considering the potential risk of exposure to known or unknown COVID-19 case.

We will estimate the seroprevalence of SARS-CoV-2 infection along with 95% confidence intervals using appropriate sampling weights and considering the cluster sampling used for the survey. We will define an antibody positive case as an individual whose sera tested positive on either of the two assays. Hence the sero-prevalence estimated will be based on presence of either N antibodies or S1-RBD antibodies. The final prevalence estimates will be calculated after adjusting for the IgG test performance characteristics of both assays.

We will use the survey data analysis module in STATA software (StataCorp LLC, Texas, USA).

We will separately estimate the seroprevalence of infections only among the adults. In order to maintain comparability with the previous surveys, we will randomly select one adult survey participant per household from the survey database.

We will estimate proportion of individuals with COVID-19 vaccination along with 95% CI. We will also estimate the seroprevalence with 95% CI for SARS-CoV-2 infection by age, sex, locality, occupation, symptom status, contact history, RT-PCR testing and confirmation status.

We will estimate the infection-to-case ratio as the number of individuals with SARS-CoV-2 infection (as per IgG detection) compared to the number of RT-PCR reported cases of COVID-19. We will apply the seroprevalence to the total population above 10 years projected for the year 2020 using 2011 census data to estimate the number of infections in the country. The published literature indicates that the IgG antibodies against SARS-CoV-2 infection start appearing by the end of first week, and most cases are IgG positive by the end of the second week (10). We will consider the number of reported COVID-19 cases 15 days and one week before the initiation of serosurvey to estimate the plausible range of the number of infections. We will estimate the range of the infection fatality ratio (IFR) assuming a three-week lag time from infection to death (11,12)

B] Health care workers

We will estimate the prevalence of SARS-CoV-2 antibodies along with 95% CI nationally among healthcare workers.

Ethical considerations

Ethical approval will be obtained. Informed Consent and assent will be obtained as per the ICMR ethics guidelines (2017): less 7 years parental consent, 7-11 years – Oral assent and parental/LAR written consent; 12-17 years –Written assent and parental/LAR consent; ≥18 years – Written consent.

Informed consent will be obtained from everyone after reading out the participant information sheet. Individuals will be encouraged to ask questions regarding study details. Individuals who can sign their name will be asked to sign the consent form. Illiterate individuals will be read the consent form, given the opportunity to ask questions, and documented by thumbprint and witnessed by an independent literate witness who is not part of the study team.

Interviews will happen in a place as per the convenience of the participants to ensure privacy. All data will be stored securely under the investigator’s responsibility, focusing on ensuring the participant’s confidentiality. Final report will be based on aggregate data without any identifying information. eCRFs will be used to collect data. A database with electronic tracking, password restricted access, audit trail, with time and date stamps on data entry and edits, will be used. Approval for the protocol will be obtained from the ethics committee of the lead institution.

Implementation plan

The survey will be led by ICMR-NIE in partnership with other ICMR institutes and the State Health authorities, with the active engagement of the District Health Administration.

The serosurvey protocol will be shared and discussed with the respective State health authorities. State health department/district/hospital authorities will be actively engaged to ensure smooth operationalization of the surveillance teams and also to reduce any stigma arising for the participants due to their participation in this surveillance at the community level. The survey will be conducted primarily as a public health surveillance activity in the context of epidemic disease.

Expected benefits

The fourth population-based serosurvey will help us to determine the prevalence of SARS-CoV-2 antibodies at the community level. It would also provide a better understanding of the transmission dynamics of SARS-CoV-2 infection among various population-level demographics and health care workers. This would serve as an objective measure of the impact of second wave on the transmission of SARS-CoV-2 infection in the country. The survey also would provide an opportunity to estimate the proportion vaccinated for COVID-19 in the community level.

References

1. Government of India. COVID-19 Dashboard. Available from: https://www.mygov.in/covid-19, accessed on May 20, 2021.
2. Govt. of India. COVID-19 Dashboard, 29 May 2021. Available from: https://www.mygov.in/covid-19, accessed on 29 May 2021.
3. Murhekar MV, Bhatnagar T, Selvaraju S, et al. Prevalence of SARS-CoV-2 infection in India: Findings from the national serosurvey, May-June 2020. Indian J Med Res. 2020 Jul & Aug;152(1 & 2):48-60. doi: 10.4103/ijmr.IJMR_3290_20.
4. Murhekar MV, Bhatnagar T, Selvaraju S, et al. SARS-CoV-2 antibody seroprevalence in India, August-September, 2020: findings from the second nationwide household serosurvey. Lancet Glob Health. 2021 Mar;9(3):e257-e266. doi: 10.1016/S2214-109X(20)30544-1. Epub 2021 Jan 27. PMID: 33515512; PMCID: PMC7906675.
5. Murhekar MV, Bhatnagar T, Thangaraj JWV, et al.SARS-CoV-2 sero-prevalence among general population and healthcare workers in India, December 2020 - January 2021. Int J Infect Dis. 2021 May 19:S1201-9712(21)00442-2. doi:

10.1016/j.ijid.2021.05.040. Epub ahead of print. PMID: 34022338; PMCID: PMC8132496

6. Ministry of Health and Family Welfare. Frequently Asked Questions – COVID vaccination. Government of India. https://www.mohfw.gov.in/covid_vaccination/vaccination/faqs.html#who-will-get-the-vaccine
7. Ministry of Health and Family Welfare, Government of India. Cumulative Coverage Report of COVID-19 Vaccination dated May 20, 2021. Available at https://www.mohfw.gov.in/pdf/CumulativeCovidVaccinationCoverageReport19thMay2021.pdf. Accessed on May 29, 2021
8. World Health Organization. Population-based age-stratified seroepidemiological investigation protocol for coronavirus 2019 (COVID-19) infection, 26 May 2020. Version 2.0.
9. U.S. Food & Drug Administration. EUA authorized serology test performance. Available from: https://www.fda.gov/medical-devices/coronavirus-disease-2019-covid-19-emergency-use-authorizations-medical-devices/eua-authorized-serology-test-performance
10. Long Q, Liu B, Deng H, Wu G, Deng K, Chen Y, et al. Antibody responses to SARS-CoV-2 in patients with COVID-19. Nat Med (2020). https://doi.org/10.1038/s41591-020-0897-1.
11. Bendavid E, Mulaney B, Sood N, Shah S, Ling E, Bromley-Dulfano R, et al. COVID-19 antibody seroprevalence in Santa Clara county, California. MedRxiv; April 17, 2020. doi: https://doi.org/10.1101/2020.04.14.20062463.
12. Pearce N, Vandenbroucke JP, VanderWeele TJ, Greenland S. Accurate Statistics on COVID-19 Are Essential for Policy Guidance and Decisions. Am J Public Health. 2020 Apr 23:e1-e3. doi: 10.2105/AJPH.2020.305708.

Population-based serosurvey to estimate prevalence of SARS-CoV-2 antibodies in India: Fourth survey, June - July 2021

Participant Information Sheet - Adults

Warm Greetings!
We are from <Insert name of the department/institute>. We are conducting a survey to understand the extent of transmission of coronavirus in the community and monitor the trends of transmission of SARS-CoV-2 infection in this area.

1. Purpose

SARS-CoV-2 causing Coronavirus disease [COVID 19] is a new infection that was not reported before. The purpose of this survey is to generate information on the extent of COVID-19 spread among the general population in this area. The development of antibodies is a sign of exposure to COVID-19 infection. This survey is likely to help us understand the extent of transmission in different age groups and genders of the population. The knowledge gained might help the program managers to evolve guidelines to help prevent and control the spread of infection in communities.

2. Procedures

If you agree to participate in this survey, then we will ask you a few questions about your personal and family background, household details, exposure and medical history. We will take 3 ml of venous blood sample taking all precautions to cause minimum discomfort to you. Blood samples will then be tested for the presence of antibodies against COVID-19. The interview and blood collection procedure might take around 20 minutes, and we sincerely request you to give us that time and participate in the survey. We may preserve the serum samples and use it in future for research studies only. We will not allow any commercial use of your samples.

3. Risk of participation

You may experience some discomforts while participating. During the interview for data collection, you may have emotional discomfort while recollecting the circumstances under which the contact/exposure to COVID 19 case might have taken place. If needed, you can contact the helpline where trained counsellors will answer your questions and suggest help.

There might be mild discomfort during the collection of blood at the site of blood collection. We will employ trained personnel to minimize the discomfort. You may also feel some anxiety since the person collecting the sample will be wearing Personal Protection Equipment (PPE).

4. Benefits

Your participation will help us to understand the dynamics of transmission of the COVID-19. After analyzing the results, a report will be shared with the health officials so that adequate preventive measures can be ensured in future. You need not have to pay for the tests, and any further management will be offered by the State health authorities as per the State guidelines. You will not incur any expenditure for the survey procedures and will not receive any reimbursements for participation.

We will share the test results with you after the completion of laboratory analysis. A positive result will mean that you have been exposed to SARS-CoV-2 virus infection in the past. However, at this point, we cannot be sure whether you will not get infected with the virus again. A negative result will mean that you have not yet developed an antibody against the virus.

More than the individual benefit, by taking part in the study, you will be doing a contribution to society and humanity. The knowledge that we gain from this survey will contribute to advancing medical and public health knowledge and practice in our country.

5. Participants rights

Taking part in this survey is voluntary. You can choose not to take part in. You can choose not to answer a specific question. You can also stop answering these questions at any time without having to provide a reason. This will not affect any of your rights, including the right to seek care in a Government facility.

6. Privacy and confidentiality

We understand the difficult times that you are facing. Appropriate care will be taken to safeguard your identity and personal information. Your data will be collected using a structured interview schedule by a trained interviewer.

We will collect blood samples to detect antibodies against COVID19 virus. Your specimens will be processed using code numbers. The left-over samples will be preserved for future research. The laboratory results will be given to the study team and will be entered into the study database.

We propose to collect relevant data that includes socio-demographics, exposure details of the contact with that of the COVID-19 patients. Data related to you will be used only for scientific purposes, and your records will be kept safely to maintain confidentiality.

7. Compensation

No compensation in cash or kind will be paid to you for taking part in this survey.

8. Contact details

If you wish to find out more about this survey, you can ask me all the questions you want and seek clarifications. You can contact the <Name and contact number of PI> for more details. For queries related to the rights as a study participant, please write to:

For queries related to the rights as a study participant, please write to: The Chairperson, NIEIHEC, National Institute of Epidemiology (ICMR), 2nd Main Road, Ayapakkam, Chennai –600077, Ph: 044-26136234).

If you are willing to participate, we will go ahead now.

Population-based serosurvey to estimate prevalence of SARS-CoV-2 antibodies in India: Fourth survey, June - July 2021

Indian Council of Medical Research

Informed consent form - Adults

Participant ID no:

I have read the participant information sheet (It has been read to me), and the details provided in the participant information sheet and consent form have been explained to me. I was given the opportunity to ask questions, and they have been answered to my satisfaction. I was given the time and freedom to decide to participate at my own free will.

I hereby consent voluntarily to take part in this surveillance and understand that I have the right to refuse to answer any question/ item and withdraw from the survey at any time. I am also aware that my withdrawal from the survey will not affect my future medical care. By signing the consent, I agree to the use of the data provided by me for surveillance/research purposes and consent to be contacted for further information.

Kindly tick (✔) the relevant option for consenting

( ) I consent for the survey team to interview and contact me for follow-up related to the survey
( ) I consent for providing the blood sample
( ) I permit to store the left-over (if any) blood samples for future research

Date	Name of the participant	Signature/thumb impression of the participant

[The literate witness selected by the participant must sign the informed consent form. The witness should not have any relationship with the research team; If the participant doesn't want to disclose his / her participation details to others, in view of respecting the wishes of the participant, he / she can be allowed to waive from the witness procedure (This is applicable to literate participant ONLY). This should be documented by the study staff by getting signature from the prospective participant]

"I have witnessed the accurate reading of the consent form to the potential participant, and the individual has had the opportunity to ask questions. I confirm that the individual has given consent freely."

Date	Name of the witness	Signature of the witness
Date	Name of the investigator/ person obtaining consent	Signature of the investigator/person obtaining consent

Population-based serosurvey to estimate prevalence of SARS-CoV-2 antibodies in India: Fourth survey, June - July 2021

Participant Information Sheet for children

Greetings!

We would like to ask you to take part in a research study. Before you decide, it is important for you to understand what the study is about and what will happen to you if you take part. Please read this leaflet carefully and ask us about anything that you do not understand.

What is the study for?

This study is trying to understand spread of newly spreading corona infection in community. We are working with doctors and researchers from all over India who are helping us to gather information and blood samples. The results of all this work may help us to understand the spread of the infection and might help in prevention and control

Why have I been chosen to take part?

You are chosen because you are more than 6 years old, and the newly spreading corona virus infection could infect irrespective of age therefore your participation considered very important

What will happen if I take part?

We will ask you to tell us how you feel by asking you some questions. We will also ask your parents several information about you, if you agree. We will also collect 3 ml (about one teaspoonful) of blood from your vein. We will test these samples for antibodies against this new corona virus infection.

What will happen to my data?

The information we collect about you and family details which will be labelled only with your study number. Your name will be stored separately and safely. Only the interviewer and researcher will know who you are. If there is any local injury at the blood prick site, the same will be taken care by health workers.

Do I have to take part?

No. You do not have to take part if you do not want to. The research may not help you, but we hope the information learned from the study will help other members in the future. You can change your mind about taking part at any time without saying why. If you withdraw from the study, it will not affect any treatment you might need in the future.

What happens afterwards?

The study results will be made available for you to read after few days of study. We will send you a simple text message about the result of study.

Contact details

If you wish to find out more about this survey, you can ask me all the questions you want and seek clarifications. You can contact the <Name and contact number of PI> for more details. For queries related to the rights as a study participant, please write to:

For queries related to the rights as a study participant, please write to: The Chairperson, NIEIHEC, National Institute of Epidemiology (ICMR), 2nd Main Road, Ayapakkam, Chennai –600077, Ph: 044-26136234).

If you are willing to participate, we will go ahead now

Informed Parental Consent Form

(for participants between 6 years and 17 years of age)

Population-based serosurvey to estimate prevalence of SARS-CoV-2 antibodies in India: Fourth survey, June - July 2021

Participant ID No:

"I have read the foregoing information, or it has been read to me. I have had the opportunity to ask questions about it and any questions I have asked have been answered to my satisfaction. I give my consent voluntarily to include my child as a participant in this study and understand that I have the right to withdraw from the study at any time without in any way it affecting my further medical care."

- ☐ I give my consent for my child to be included as a participant in this study.
- ☐ I agree to permit storage of my child's blood/serum sample for future analyses
- ☐ I am also aware of my right that I need not agree to take part in the study without assigning any reasons to do so.
- ☐ I agree for my child's participation in the study.

Date	Name of the Child/Participant	Signature/thumb impression of the Parent of the Child/Participant

[The literate witness selected by the participant must sign the informed consent form. The witness should not have any relationship with the research team; If the participant doesn't want to disclose his / her participation details to others, in view of respecting the wishes of the participant, he / she can be allowed to waive from the witness procedure (This is applicable to literate participant ONLY). This should be documented by the study staff by getting signature from the prospective participant]

"I have witnessed the accurate reading of the consent form to the potential participant and the individual has had the opportunity to ask questions. I confirm that the individual has given consent freely

Date	Name of the witness	Signature of the witness

Date	Name of the interviewer	Signature of the interviewer

Informed Assent Form

(for participants between 12 years and 17 years of age)

Population-based serosurvey to estimate prevalence of SARS-CoV-2 antibodies in India: Fourth survey, June - July 2021

Participant ID No:

"I have read the foregoing information, or it has been read to me. I have had the opportunity to ask questions about it and any questions I have asked have been answered to my satisfaction. I consent voluntarily to participate as a participant in this study and understand that I can say NO to taking part in the study, even if my parents have agreed to my participation. I understand I have the right to withdraw from the study at any time without giving any reason, without anyone upset at me, or my medical care or legal rights being affected."

Date	Name of the child participant	Signature/thumb impression of the child participant

[The literate witness selected by the participant must sign the informed consent form. The witness should not have any relationship with the research team; If the participant doesn't want to disclose his / her participation details to others, in view of respecting the wishes of the participant, he / she can be allowed to waive from the witness procedure (This is applicable to literate participant ONLY). This should be documented by the study staff by getting signature from the prospective participant]

"I have witnessed the accurate reading of the consent form to the potential participant and the individual has had the opportunity to ask questions. I confirm that the individual has given consent freely."

Date	Name of the witness	Signature of the witness

Date	Name of the investigator/person obtaining consent	Signature of the investigator / person obtaining consent

Parent/guardian has signed an informed consent: Yes ____________ No____________
Initialed by the researcher
