# Supplementary material for: Seroprevalence of IgG antibodies against SARS-CoV-2 among the general population and healthcare workers in India, June–July 2021: A population-based cross-sectional study
Source: PLoS Med. 2021 Dec 10;18(12):e1003877. doi: 10.1371/journal.pmed.1003877 (PMC8726494; doi:10.1371/journal.pmed.1003877)
Supplement: S1 Table — (DOCX) [file pmed.1003877.s004.docx]

**S1 Table: Seroprevalence of IgG antibodies against Nucleocapsid protein by selected characteristics, June-July 2021**

| Characteristics | General population | | | Healthcare workers | | |
| --- | --- | --- | --- | --- | --- | --- |
|  | **No. tested** | **No. positive**  **(anti-N antibodies)** | **Weighted and test-performance-adjusted seroprevalence**  **% (95% CI)** | **No. tested** | **No. positive**  **(anti-N antibodies)** | **Test-performance-adjusted seroprevalence**  **% (95% CI)** |
| Age (years) | | | | | | |
| 6-9 | 2892 | 1076 | 36.5 (34.4 - 38.8) | - | - | - |
| 10-17 | 5798 | 2329 | 39.0 (37.2 - 40.7) | - | - | - |
| 18-44 | 12522 | 4847 | 37.7 (36.2 - 39.1) | 5133 | 1635 | 31.0 (28.7 - 33.4) |
| 45-60 | 5545 | 2149 | 38.3 (36.4 - 40.0) | 1997 | 624 | 30.2 (27.5 - 33.1) |
| >60 | 2218 | 888 | 41.5 (39.1 - 44.0) | 122 | 46 | 36.5 (28.1 - 46.0) |
| Sex | | | | | | |
| Male | 13783 | 5311 | 37.1 (35.8 - 38.6) | 3523 | 1162 | 31.5 (29.0 - 34.0) |
| Female | 15160 | 5967 | 39.2 (37.9 - 40.6) | 3722 | 1141 | 30.3 (27.9 - 32.8) |
| Other | 32 | 11 | 16.4 (6.1 - 36.3) | 7 | 2 | 26.6 (6.2 - 65.7) |
| Area of residence | | | | | | |
| Rural | 21794 | 8556 | 39.0 (37.6 - 40.4) | - | - | - |
| Urban non-slum | 5266 | 1997 | 37.2 (34.7 - 39.8) | - | - | - |
| Urban slum | 1915 | 736 | 37.3 (33.1 - 41.6) | - | - | - |
| History of COVID-19 related symptoms since 1 Jan 2021 | | | | | | |
| Yes | 1748 | 808 | 51.6 (48.7 - 54.6) | 925 | 422 | 45.9 (41.8 - 50.1) |
| No | 27227 | 10481 | 37.6 (36.3 - 38.8) | 6327 | 1883 | 28.7 (26.5 - 31.0) |
| Results of COVID-19 testing | | | | | | |
| Reported positive for COVID-19 | 782 | 464 | 63.4 (59.8 - 66.7) | 1354 | 630 | 46.6 (42.9 - 50.3) |
| Reported Negative for COVID-19 | 3419 | 1222 | 38.1 (35.8 - 40.3) | 3395 | 922 | 26.3 (24.0 - 28.9) |
| Don’t know | 171 | 55 | 34.5 (24.4 - 46.3) | 143 | 56 | 37.4 (26.6 - 49.8) |
| COVID-19 vaccination among adults | | | | | | |
| 0 dose | 12599 | 5122 | 40.3 (38.9 - 41.7) | 759 | 339 | 43.2 (39.0 - 47.5) |
| 1 dose | 5038 | 1898 | 36.1 (34.3 - 38.0) | 972 | 358 | 35.9 (32.2 - 39.8) |
| 2 doses | 2631 | 856 | 31.3 (29.1 - 33.5) | 5521 | 1608 | 28.3 (26.2 - 30.5) |
| Vaccine type | | | | | | |
| Covaxin | 587 | 274 | 50.6 (45.7 - 55.5) | 498 | 255 | 51.6 (46.5 - 56.8) |
| Covishield | 6945 | 2422 | 32.7 (30.9 - 34.5) | 5973 | 1702 | - 1. (25.5 - 29.8) |
