# Supplementary material for: Seroprevalence of IgG antibodies against SARS-CoV-2 among the general population and healthcare workers in India, June–July 2021: A population-based cross-sectional study
Source: PLoS Med. 2021 Dec 10;18(12):e1003877. doi: 10.1371/journal.pmed.1003877 (PMC8726494; doi:10.1371/journal.pmed.1003877)
Supplement: S2 Table — (DOCX) [file pmed.1003877.s005.docx]

**S2 Table: Cluster adjusted proportion of individuals with SARS-CoV-2 IgG antibodies by states, Jun-Jul 2021**

| State/Union Territory | Total Tested | Number positive for either anti-N and or anti-S-RBD antibody | Cluster Adjusted Seroprevalence %  (95% CI) |
| --- | --- | --- | --- |
| Madhya Pradesh | 1229 | 683 | 80.0 (76.3 - 83.2) |
| Rajasthan | 1226 | 628 | 77.1 (73.2 - 80.7) |
| Bihar | 2461 | 1174 | 76.7 (73.9 - 79.3) |
| Gujarat | 1219 | 540 | 76.4 (72.4 - 80.1) |
| Chhattisgarh | 1198 | 509 | 75.7 (71.6 - 79.4) |
| Uttarakhand | 401 | 202 | 74.2 (66.7 - 80.6) |
| Uttar Pradesh | 3733 | 1848 | 71.7 (69.3 - 74.1) |
| Andhra Pradesh | 1260 | 462 | 71.2 (66.8 - 75.2) |
| Karnataka | 1326 | 535 | 70.3 (65.9 - 74.4) |
| Tamil Nadu | 1258 | 524 | 70.1 (65.6 - 74.2) |
| Odisha | 1230 | 437 | 68.9 (64.3 - 73.1) |
| Punjab | 1581 | 597 | 67.2 (63.2 - 71.0) |
| Telangana | 1373 | 512 | 63.5 (58.8 - 67.9) |
| Jammu & Kashmir | 430 | 124 | 63.4 (55.0 - 71.0) |
| Himachal Pradesh | 400 | 82 | 62.5 (54.0 - 70.3) |
| Jharkhand | 1231 | 415 | 61.9 (57.1 - 66.6) |
| West Bengal | 2042 | 529 | 61.3 (57.6 - 65.0) |
| Haryana | 398 | 137 | 60.4 (51.8 - 68.4) |
| Maharashtra | 2468 | 785 | 58.4 (54.9 - 61.8) |
| Assam | 1203 | 285 | 50.3 (45.3 - 55.3) |
| Kerala | 1308 | 281 | 44.3 (39.5 - 49.2) |
