# Supplementary material for: Seroprevalence of IgG antibodies against SARS-CoV-2 among the general population and healthcare workers in India, June–July 2021: A population-based cross-sectional study
Source: PLoS Med. 2021 Dec 10;18(12):e1003877. doi: 10.1371/journal.pmed.1003877 (PMC8726494; doi:10.1371/journal.pmed.1003877)
Supplement: S3 Table — (DOCX) [file pmed.1003877.s006.docx]

**S3 Table: Unweighted proportion of individuals with SARS-CoV-2 IgG antibodies by districts, Jun-Jul 2021**

| District | State | District Population | Cluster Population* | Total Tested | Number positive for anti-N antibody (%) | Number positive for anti-S1-RBD antibody (%) | Number positive for anti-N and/or anti-S1-RBD antibodies (%) |
| --- | --- | --- | --- | --- | --- | --- | --- |
| Buxar | Bihar | 1706352 | 66081 | 400 | 216 (54.0) | 335 (83.8) | 347 (86.8) |
| Gwalior | Madhya Pradesh | 2032036 | 119030 | 416 | 219 (52.6) | 330 (79.3) | 340 (81.7) |
| Dewas | Madhya Pradesh | 1563715 | 32216 | 407 | 230 (56.5) | 319 (78.4) | 330 (81.1) |
| Vizianagaram | Andhra Pradesh | 2344474 | 39835 | 434 | 196 (45.2) | 340 (78.3) | 346 (79.7) |
| Surguja | Chhattisgarh | 2359886 | 14684 | 397 | 176 (44.3) | 301 (75.8) | 316 (79.6) |
| Chennai | Tamil Nadu | 4646732 | 448245 | 415 | 184 (44.3) | 320 (77.1) | 330 (79.5) |
| Madhubani | Bihar | 4487379 | 81720 | 424 | 213 (50.2) | 327 (77.1) | 337 (79.5) |
| Jalor | Rajasthan | 1828730 | 37803 | 409 | 207 (50.6) | 310 (75.8) | 323 (79.0) |
| Sabar Kantha | Gujarat | 2428589 | 34580 | 402 | 183 (45.5) | 303 (75.4) | 313 (77.9) |
| Arwal | Bihar | 700843 | 56456 | 410 | 202 (49.3) | 302 (73.7) | 317 (77.3) |
| Balrampur | Uttar Pradesh | 2148665 | 41769 | 431 | 192 (44.5) | 317 (73.5) | 331 (76.8) |
| Rajsamand | Rajasthan | 1156597 | 16065 | 410 | 215 (52.4) | 294 (71.7) | 308 (75.1) |
| Narmada | Gujarat | 590297 | 18345 | 398 | 160 (40.2) | 289 (72.6) | 297 (74.6) |
| Dausa | Rajasthan | 1634409 | 34954 | 407 | 206 (50.6) | 280 (68.8) | 303 (74.4) |
| Begusarai | Bihar | 2970541 | 73706 | 417 | 186 (44.6) | 303 (72.7) | 310 (74.3) |
| Ujjain | Madhya Pradesh | 1986864 | 44409 | 406 | 234 (57.6) | 286 (70.4) | 301 (74.1) |
| Mau | Uttar Pradesh | 2205968 | 27959 | 455 | 245 (53.8) | 328 (72.1) | 337 (74.1) |
| Saharanpur | Uttar Pradesh | 3466382 | 43653 | 399 | 220 (55.1) | 275 (68.9) | 295 (73.9) |
| Mahisagar | Gujarat | 994624 | 21194 | 419 | 197 (47.0) | 294 (70.2) | 308 (73.5) |
| Ludhiana | Punjab | 3498739 | 119520 | 394 | 158 (40.1) | 279 (70.8) | 288 (73.1) |
| Garhwal | Uttarakhand | 687271 | 12634 | 401 | 202 (50.4) | 273 (68.1) | 293 (73.1) |
| Unnao | Uttar Pradesh | 3108367 | 25822 | 395 | 202 (51.1) | 270 (68.4) | 288 (72.9) |

| District | State | | District Population | | Cluster Population | | Total Tested | Number positive for anti-N antibody (%) | Number positive for anti-S1-RBD antibody (%) | | Number positive for anti-N and/or anti-S1-RBD antibodies (%) |  |
| --- | --- | --- | --- | --- | --- | --- | --- | --- | --- | --- | --- | --- |
| Auraiya | Uttar Pradesh | | 1379545 | | 38100 | | 398 | 234 (58.8) | 259 (65.1) | | 290 (72.9) |  |
| Kabeerdham | Chhattisgarh | | 822526 | | 11999 | | 403 | 192 (47.6) | 267 (66.3) | | 293 (72.7) |  |
| Ganjam | Odisha | | 3529031 | | 25467 | | 419 | 154 (36.8) | 297 (70.9) | | 304 (72.6) |  |
| Bangalore | Karnataka | | 9621551 | | 432255 | | 447 | 165 (36.9) | 313 (70.0) | | 323 (72.3) |  |
| Bijapur | Chhattisgarh | | 255230 | | 21092 | | 398 | 141 (35.4) | 272 (68.3) | | 285 (71.6) |  |
| Bareilly | Uttar Pradesh | | 4448359 | | 47828 | | 400 | 185 (46.3) | 261 (65.3) | | 284 (71.0) |  |
| Gautam Buddha Nagar | Uttar Pradesh | | 1648115 | | 227497 | | 398 | 186 (46.7) | 265 (66.6) | | 281 (70.6) |  |
| Muzaffarpur | Bihar | | 4801062 | | 68761 | | 404 | 194 (48.0) | 264 (65.3) | | 281 (69.6) |  |
| Tiruvannamalai | Tamil Nadu | | 2464875 | | 40859 | | 415 | 168 (40.5) | 282 (68.0) | | 288 (69.4) |  |
| Gulbarga | Karnataka | | 2566326 | | 36473 | | 428 | 177 (41.4) | 288 (67.3) | | 296 (69.2) |  |
| Simdega | Jharkhand | | 599578 | | 30948 | | 400 | 151 (37.8) | 261 (65.3) | | 275 (68.8) |  |
| Gonda | Uttar Pradesh | | 3433919 | | 25921 | | 457 | 232 (50.8) | 293 (64.1) | | 314 (68.7) |  |
| Bankura | West Bengal | | 3596674 | | 21114 | | 411 | 144 (35.0) | 274 (66.7) | | 281 (68.4) |  |
| Kamareddy | Telangana | | 974227 | | 33723 | | 471 | 186 (39.5) | 309 (65.6) | | 322 (68.4) |  |
| Chitradurga | Karnataka | | 1659456 | | 30040 | | 451 | 193 (42.8) | 293 (65.0) | | 307 (68.1) |  |
| Krishna | Andhra Pradesh | | 4517398 | | 171299 | | 409 | 144 (35.2) | 266 (65.0) | | 278 (68.0) |  |
| Jalandhar | Punjab | | 2193590 | | 86960 | | 398 | 148 (37.2) | 261 (65.6) | | 270 (67.8) |  |
| Purnia | Bihar | | 3264619 | | 57806 | | 406 | 163 (40.1) | 263 (64.8) | | 275 (67.7) |  |
| Gurdaspur | Punjab | | 2298323 | | 30441 | | 390 | 144 (36.9) | 247 (63.3) | | 261 (66.9) |  |
| Latehar | Jharkhand | | 726978 | | 14102 | | 422 | 180 (42.7) | 239 (56.6) | | 279 (66.1) |  |
| Rayagada | Odisha | | 967911 | | 17728 | | 411 | 134 (32.6) | 259 (63.0) | | 271 (65.9) |  |
| Koraput | Odisha | | 1379647 | | 14039 | | 400 | 149 (37.3) | 245 (61.3) | | 263 (65.8) |  |
| South Twenty-Four Parganas | West Bengal | | 8161961 | | 72661 | | 412 | 106 (25.7) | 257 (62.4) | | 260 (63.1) |  |
| Pulwama | Jammu & Kashmir | | 560440 | | 30595 | | 430 | 124 (28.8) | 263 (61.2) | | 271 (63.0) |  |
| District | | **State** | | **District Population** | | **Cluster Population** | **Total Tested** | **Number positive for anti-N antibody (%)** | | **Number positive for anti-S1-RBD antibody (%)** | **Number positive for anti-N and/or anti-S1-RBD antibodies (%)** | |
| Sri Potti Sriramulu Nellore | | Andhra Pradesh | | 2963557 | | 66168 | 417 | 122 (29.3) | | 253 (60.7) | 261 (62.6) | |
| Parbhani | | Maharashtra | | 1836086 | | 28328 | 420 | 130 (31.0) | | 252 (60.0) | 262 (62.4) | |
| Nanded | | Maharashtra | | 3361292 | | 34371 | 403 | 141 (35.0) | | 242 (60.0) | 251 (62.3) | |
| Kullu | | Himachal Pradesh | | 437903 | | 20383 | 400 | 82 (20.5) | | 243 (60.8) | 248 (62.0) | |
| Alipurduar | | West Bengal | | 1426018 | | 76702 | 408 | 83 (20.3) | | 241 (59.1) | 247 (60.5) | |
| Nalgonda | | Telangana | | 3488809 | | 52021 | 451 | 171 (37.9) | | 252 (55.9) | 273 (60.5) | |
| Jangoan | | Telangana | | 561422 | | 43661 | 451 | 155 (34.4) | | 265 (58.8) | 271 (60.1) | |
| Kurukshetra | | Haryana | | 964655 | | 38384 | 398 | 137 (34.4) | | 218 (54.8) | 239 (60.1) | |
| Ahmadnagar | | Maharashtra | | 4543159 | | 66513 | 419 | 131 (31.3) | | 243 (58.0) | 251 (59.9) | |
| Coimbatore | | Tamil Nadu | | 3458045 | | 98833 | 428 | 172 (40.2) | | 244 (57.0) | 252 (58.9) | |
| Patiala | | Punjab | | 1895686 | | 40481 | 399 | 147 (36.8) | | 210 (52.6) | 233 (58.4) | |
| Jalgaon | | Maharashtra | | 4229917 | | 28930 | 414 | 155 (37.4) | | 211 (51.0) | 241 (58.2) | |
| Jyotiba Phule Nagar | | Uttar Pradesh | | 1840221 | | 50066 | 400 | 152 (38.0) | | 205 (51.3) | 230 (57.5) | |
| Purba Medinipur | | West Bengal | | 5095875 | | 28913 | 401 | 83 (20.7) | | 216 (53.9) | 225 (56.1) | |
| Jhargram | | West Bengal | | 1290246 | | 55524 | 410 | 113 (27.6) | | 222 (54.1) | 230 (56.1) | |
| Kamrup Metropolitan | | Assam | | 1253938 | | 130120 | 399 | 110 (27.6) | | 211 (52.9) | 219 (54.9) | |
| Sangli | | Maharashtra | | 2822143 | | 44265 | 410 | 102 (24.9) | | 218 (53.2) | 225 (54.9) | |
| Udalguri | | Assam | | 831668 | | 22117 | 401 | 82 (20.4) | | 199 (49.6) | 207 (51.6) | |
| Bid | | Maharashtra | | 2585049 | | 27600 | 402 | 126 (31.3) | | 169 (42.0) | 202 (50.2) | |
| Thrissur | | Kerala | | 3121200 | | 143187 | 437 | 107 (24.5) | | 206 (47.1) | 216 (49.4) | |
| Pakur | | Jharkhand | | 900422 | | 25242 | 409 | 84 (20.5) | | 180 (44.0) | 199 (48.7) | |
| Karbi Anglong | | Assam | | 956313 | | 8046 | 403 | 93 (23.1) | | 162 (40.2) | 179 (44.4) | |
| Palakkad | | Kerala | | 2809934 | | 151509 | 439 | 94 (21.4) | | 184 (41.9) | 190 (43.3) | |
| Ernakulam | | Kerala | | 3282388 | | 264858 | 432 | 80 (18.5) | | 169 (39.1) | 175 (40.5) | |

*****Cumulative population of 10 clusters selected in each district as per 2011 census
