# Supplementary material for: Seroprevalence of IgG antibodies against SARS-CoV-2 among the general population and healthcare workers in India, June–July 2021: A population-based cross-sectional study
Source: PLoS Med. 2021 Dec 10;18(12):e1003877. doi: 10.1371/journal.pmed.1003877 (PMC8726494; doi:10.1371/journal.pmed.1003877)
Supplement: S4 Table — (DOCX) [file pmed.1003877.s007.docx]

**S4 Table: Unweighted proportion of individuals with SARS-CoV-2 IgG antibodies among unvaccinated individuals aged >=10 years* by districts, during third (Dec 2020-Jan 2021) and fourth (Jun-Jul 2021) national serosurveys**

| State | District | General Population (Unvaccinated and >=10 Years) Round 3 | | | | General Population (Unvaccinated and >=10 Years) Round 4 | | | | Ratio of anti-N/anti-S positivity during round 4 and round 3 |
| --- | --- | --- | --- | --- | --- | --- | --- | --- | --- | --- |
|  |  | **Total Tested** | **No. positive for ant-N (%)** | **No. positive for anti-S Positive (%)** | **No. positive for anti-N or anti-S Positive (%)** | **Total Tested** | **No. positive for ant-N (%)** | **No. positive for anti-S Positive (%)** | **No. positive for anti-N or anti-S Positive (%)** |  |
| Gujarat | Mahisagar | 407 | 11 (2.7) | 15 (3.7) | 20 (4.9) | 171 | 84 (49.1) | 108 (63.2) | 113 (66.1) | 13.5 |
| Chhattisgarh | Surguja | 396 | 33 (8.3) | 31 (7.8) | 43 (10.9) | 250 | 126 (50.4) | 186 (74.4) | 199 (79.6) | 7.3 |
| Gujarat | Narmada | 401 | 41 (10.2) | 42 (10.5) | 47 (11.7) | 216 | 91 (42.1) | 141 (65.3) | 148 (68.5) | 5.9 |
| Uttar Pradesh | Saharanpur | 396 | 41 (10.4) | 51 (12.9) | 60 (15.2) | 294 | 167 (56.8) | 192 (65.3) | 211 (71.8) | 4.7 |
| Uttar Pradesh | Gonda | 417 | 31 (7.4) | 54 (12.9) | 60 (14.4) | 285 | 139 (48.8) | 178 (62.5) | 191 (67.0) | 4.7 |
| Madhya Pradesh | Dewas | 390 | 51 (13.1) | 58 (14.9) | 73 (18.7) | 274 | 160 (58.4) | 209 (76.3) | 219 (79.9) | 4.3 |
| Rajasthan | Rajsamand | 404 | 58 (14.4) | 57 (14.1) | 69 (17.1) | 265 | 148 (55.8) | 179 (67.5) | 192 (72.5) | 4.2 |
| Jharkhand | Latehar | 398 | 37 (9.3) | 48 (12.1) | 60 (15.1) | 256 | 117 (45.7) | 133 (52.0) | 163 (63.7) | 4.2 |
| Bihar | Begusarai | 413 | 48 (11.6) | 62 (15.0) | 77 (18.6) | 255 | 125 (49.0) | 172 (67.5) | 179 (70.2) | 3.8 |
| Uttar Pradesh | Balrampur | 390 | 58 (14.9) | 62 (15.9) | 80 (20.5) | 302 | 139 (46.0) | 216 (71.5) | 229 (75.8) | 3.7 |
| Uttarakhand | Garhwal | 399 | 61 (15.3) | 66 (16.5) | 77 (19.3) | 185 | 117 (63.2) | 114 (61.6) | 131 (70.8) | 3.7 |
| Uttar Pradesh | Unnao | 401 | 44 (11.0) | 66 (16.5) | 76 (19.0) | 250 | 125 (50.0) | 156 (62.4) | 171 (68.4) | 3.6 |
| Rajasthan | Dausa | 390 | 49 (12.6) | 62 (15.9) | 75 (19.2) | 249 | 132 (53.0) | 146 (58.6) | 166 (66.7) | 3.5 |
| Chhattisgarh | Kabeerdham | 399 | 54 (13.5) | 73 (18.3) | 81 (20.3) | 290 | 138 (47.6) | 184 (63.4) | 204 (70.3) | 3.5 |
| Gujarat | Sabar Kantha | 399 | 62 (15.5) | 75 (18.8) | 83 (20.8) | 220 | 96 (43.6) | 145 (65.9) | 155 (70.5) | 3.4 |
| Madhya Pradesh | Ujjain | 405 | 53 (13.1) | 78 (19.3) | 84 (20.7) | 271 | 155 (57.2) | 173 (63.8) | 188 (69.4) | 3.4 |
| Punjab | Jalandhar | 398 | 55 (13.8) | 66 (16.6) | 77 (19.3) | 245 | 85 (34.7) | 152 (62.0) | 158 (64.5) | 3.3 |

| State | District | General Population (Unvaccinated and >=10 Years) Round 3 | | | | General Population (Unvaccinated and >=10 Years) Round 4 | | | | Ratio of anti-N/anti-S positivity during round 4 and round 3 |
| --- | --- | --- | --- | --- | --- | --- | --- | --- | --- | --- |
|  |  | **Total Tested** | **No. positive for ant-N (%)** | **No. positive for anti-S Positive (%)** | **No. positive for anti-N or anti-S Positive (%)** | **Total Tested** | **No. positive for ant-N (%)** | **No. positive for anti-S Positive (%)** | **No. positive for anti-N or anti-S Positive (%)** |  |
| Haryana | Kurukshetra | 397 | 45 (11.3) | 60 (15.1) | 66 (16.6) | 243 | 78 (32.1) | 110 (45.3) | 127 (52.3) | 3.2 |
| Rajasthan | Jalor | 409 | 75 (18.3) | 74 (18.1) | 93 (22.7) | 195 | 104 (53.3) | 123 (63.1) | 134 (68.7) | 3.0 |
| Kerala | Thrissur | 418 | 50 (12.0) | 36 (8.6) | 59 (14.1) | 278 | 71 (25.5) | 109 (39.2) | 117 (42.1) | 3.0 |
| Bihar | Buxar | 422 | 71 (16.8) | 110 (26.1) | 121 (28.7) | 233 | 129 (55.4) | 189 (81.1) | 198 (85.0) | 3.0 |
| Kerala | Palakkad | 408 | 46 (11.3) | 55 (13.5) | 58 (14.2) | 325 | 72 (22.2) | 130 (40.0) | 136 (41.8) | 2.9 |
| Jharkhand | Simdega | 400 | 59 (14.8) | 68 (17.0) | 78 (19.5) | 193 | 70 (36.3) | 101 (52.3) | 110 (57.0) | 2.9 |
| Uttar Pradesh | Auraiya | 398 | 61 (15.3) | 86 (21.6) | 99 (24.9) | 304 | 186 (61.2) | 188 (61.8) | 219 (72.0) | 2.9 |
| Bihar | Muzaffarpur | 401 | 41 (10.2) | 87 (21.7) | 93 (23.2) | 275 | 141 (51.3) | 169 (61.5) | 184 (66.9) | 2.9 |
| Maharashtra | Parbhani | 426 | 46 (10.8) | 81 (19.0) | 88 (20.7) | 278 | 89 (32.0) | 154 (55.4) | 163 (58.6) | 2.8 |
| Karnataka | Chitradurga | 442 | 67 (15.2) | 96 (21.7) | 103 (23.3) | 276 | 133 (48.2) | 171 (62.0) | 182 (65.9) | 2.8 |
| Bihar | Arwal | 416 | 63 (15.1) | 109 (26.2) | 118 (28.4) | 299 | 147 (49.2) | 223 (74.6) | 233 (77.9) | 2.7 |
| Punjab | Ludhiana | 399 | 56 (14.0) | 89 (22.3) | 101 (25.3) | 271 | 113 (41.7) | 182 (67.2) | 187 (69.0) | 2.7 |
| Uttar Pradesh | Mau | 417 | 72 (17.3) | 89 (21.3) | 108 (25.9) | 319 | 172 (53.9) | 214 (67.1) | 223 (69.9) | 2.7 |
| Punjab | Patiala | 400 | 59 (14.8) | 75 (18.8) | 84 (21.0) | 272 | 107 (39.3) | 132 (48.5) | 153 (56.3) | 2.7 |
| Bihar | Madhubani | 404 | 63 (15.6) | 99 (24.5) | 113 (28.0) | 223 | 116 (52.0) | 157 (70.4) | 165 (74.0) | 2.6 |
| Madhya Pradesh | Gwalior | 402 | 68 (16.9) | 107 (26.6) | 112 (27.9) | 182 | 98 (53.8) | 127 (69.8) | 134 (73.6) | 2.6 |
| Uttar Pradesh | Bareilly | 398 | 51 (12.8) | 92 (23.1) | 97 (24.4) | 267 | 125 (46.8) | 153 (57.3) | 171 (64.0) | 2.6 |
| Bihar | Purnia | 414 | 56 (13.5) | 87 (21.0) | 98 (23.7) | 229 | 102 (44.5) | 133 (58.1) | 141 (61.6) | 2.6 |
| Punjab | Gurdaspur | 399 | 75 (18.8) | 81 (20.3) | 101 (25.3) | 241 | 89 (36.9) | 148 (61.4) | 158 (65.6) | 2.6 |
| Telangana | Kamareddy | 413 | 51 (12.3) | 102 (24.7) | 106 (25.7) | 314 | 126 (40.1) | 187 (59.6) | 199 (63.4) | 2.5 |

| State | District | General Population (Unvaccinated and >=10 Years) Round 3 | | | | General Population (Unvaccinated and >=10 Years) Round 4 | | | | Ratio of anti-N/anti-S positivity during round 4 and round 3 |
| --- | --- | --- | --- | --- | --- | --- | --- | --- | --- | --- |
|  |  | **Total Tested** | **No. positive for ant-N (%)** | **No. positive for anti-S Positive (%)** | **No. positive for anti-N or anti-S Positive (%)** | **Total Tested** | **No. positive for ant-N (%)** | **No. positive for anti-S Positive (%)** | **No. positive for anti-N or anti-S Positive (%)** |  |
| Uttar Pradesh | Jyotiba Phule Nagar | 395 | 62 (15.7) | 80 (20.3) | 92 (23.3) | 322 | 123 (38.2) | 160 (49.7) | 182 (56.5) | 2.4 |
| Jharkhand | Pakur | 397 | 44 (11.1) | 63 (15.9) | 75 (18.9) | 285 | 63 (22.1) | 113 (39.6) | 128 (44.9) | 2.4 |
| Karnataka | Gulbarga | 429 | 47 (11.0) | 113 (26.3) | 117 (27.3) | 268 | 118 (44.0) | 161 (60.1) | 168 (62.7) | 2.3 |
| Telangana | Nalgonda | 420 | 59 (14.0) | 96 (22.9) | 108 (25.7) | 327 | 132 (40.4) | 170 (52.0) | 189 (57.8) | 2.2 |
| Tamil Nadu | Coimbatore | 411 | 69 (16.8) | 91 (22.1) | 100 (24.3) | 289 | 117 (40.5) | 149 (51.6) | 156 (54.0) | 2.2 |
| Tamil Nadu | Tiruvannamalai | 409 | 86 (21.0) | 116 (28.4) | 127 (31.1) | 274 | 123 (44.9) | 182 (66.4) | 187 (68.2) | 2.2 |
| Maharashtra | Ahmadnagar | 433 | 64 (14.8) | 98 (22.6) | 106 (24.5) | 293 | 98 (33.4) | 150 (51.2) | 156 (53.2) | 2.2 |
| Kerala | Ernakulam | 420 | 38 (9.0) | 54 (12.9) | 61 (14.5) | 234 | 48 (20.5) | 70 (29.9) | 73 (31.2) | 2.2 |
| Uttar Pradesh | Gautam Buddha Nagar | 398 | 77 (19.3) | 109 (27.4) | 126 (31.7) | 284 | 130 (45.8) | 175 (61.6) | 189 (66.5) | 2.1 |
| Karnataka | Bangalore | 418 | 81 (19.4) | 126 (30.1) | 142 (34.0) | 284 | 116 (40.8) | 193 (68.0) | 201 (70.8) | 2.1 |
| West Bengal | Jhargram | 419 | 89 (21.2) | 110 (26.3) | 119 (28.4) | 347 | 105 (30.3) | 194 (55.9) | 202 (58.2) | 2.0 |
| Telangana | Jangoan | 427 | 59 (13.8) | 106 (24.8) | 112 (26.2) | 323 | 114 (35.3) | 164 (50.8) | 170 (52.6) | 2.0 |
| Maharashtra | Nanded | 420 | 61 (14.5) | 109 (26.0) | 121 (28.8) | 269 | 82 (30.5) | 138 (51.3) | 145 (53.9) | 1.9 |
| Andhra Pradesh | Sri Potti Sriramulu Nellore | 405 | 96 (23.7) | 121 (29.9) | 127 (31.4) | 242 | 76 (31.4) | 134 (55.4) | 140 (57.9) | 1.8 |
| West Bengal | Bankura | 413 | 94 (22.8) | 137 (33.2) | 143 (34.6) | 249 | 94 (37.8) | 151 (60.6) | 156 (62.7) | 1.8 |
| Andhra Pradesh | Vizianagaram | 410 | 57 (13.9) | 166 (40.5) | 172 (42.0) | 256 | 130 (50.8) | 188 (73.4) | 194 (75.8) | 1.8 |
| Odisha | Koraput | 401 | 96 (23.9) | 124 (30.9) | 140 (34.9) | 262 | 106 (40.5) | 142 (54.2) | 160 (61.1) | 1.8 |
| Maharashtra | Jalgaon | 427 | 67 (15.7) | 121 (28.3) | 135 (31.6) | 297 | 115 (38.7) | 138 (46.5) | 162 (54.5) | 1.7 |
| Maharashtra | Bid | 421 | 57 (13.5) | 98 (23.3) | 106 (25.2) | 264 | 85 (32.2) | 88 (33.3) | 114 (43.2) | 1.7 |
| Tamil Nadu | Chennai | 408 | 72 (17.6) | 166 (40.7) | 175 (42.9) | 240 | 111 (46.3) | 167 (69.6) | 174 (72.5) | 1.7 |

| State | District | General Population (Unvaccinated and >=10 Years) Round 3 | | | | General Population (Unvaccinated and >=10 Years) Round 4 | | | | Ratio of anti-N/anti-S positivity during round 4 and round 3 |
| --- | --- | --- | --- | --- | --- | --- | --- | --- | --- | --- |
|  |  | **Total Tested** | **No. positive for ant-N (%)** | **No. positive for anti-S Positive (%)** | **No. positive for anti-N or anti-S Positive (%)** | **Total Tested** | **No. positive for ant-N (%)** | **No. positive for anti-S Positive (%)** | **No. positive for anti-N or anti-S Positive (%)** |  |
| Andhra Pradesh | Krishna | 403 | 70 (17.4) | 148 (36.7) | 154 (38.2) | 238 | 90 (37.8) | 138 (58.0) | 147 (61.8) | 1.6 |
| Jammu & Kashmir | Pulwama | 419 | 99 (23.6) | 132 (31.5) | 145 (34.6) | 198 | 61 (30.8) | 104 (52.5) | 110 (55.6) | 1.6 |
| Chhattisgarh | Bijapur | 396 | 134 (33.8) | 150 (37.9) | 176 (44.4) | 274 | 109 (39.8) | 186 (67.9) | 195 (71.2) | 1.6 |
| Assam | Karbi Anglong | 413 | 71 (17.2) | 86 (20.8) | 102 (24.7) | 294 | 77 (26.2) | 98 (33.3) | 115 (39.1) | 1.6 |
| Odisha | Ganjam | 408 | 95 (23.3) | 142 (34.8) | 157 (38.5) | 205 | 86 (42.0) | 116 (56.6) | 122 (59.5) | 1.5 |
| West Bengal | Purba Medinipur | 415 | 79 (19.0) | 124 (29.9) | 133 (32.0) | 282 | 61 (21.6) | 130 (46.1) | 136 (48.2) | 1.5 |
| Odisha | Rayagada | 407 | 87 (21.4) | 154 (37.8) | 166 (40.8) | 277 | 98 (35.4) | 149 (53.8) | 161 (58.1) | 1.4 |
| West Bengal | South 24 Parganas | 414 | 103 (24.9) | 150 (36.2) | 167 (40.3) | 256 | 68 (26.6) | 138 (53.9) | 140 (54.7) | 1.4 |
| Assam | Kamrup Metropolitan | 408 | 89 (21.8) | 134 (32.8) | 150 (36.8) | 259 | 77 (29.7) | 118 (45.6) | 125 (48.3) | 1.3 |
| West Bengal | Alipurduar | 415 | 90 (21.7) | 163 (39.3) | 175 (42.2) | 310 | 64 (20.6) | 164 (52.9) | 170 (54.8) | 1.3 |
| Himachal Pradesh | Kullu | 400 | 153 (38.3) | 141 (35.3) | 166 (41.5) | 186 | 46 (24.7) | 94 (50.5) | 99 (53.2) | 1.3 |
| Assam | Udalguri | 407 | 90 (22.1) | 114 (28.0) | 142 (34.9) | 271 | 55 (20.3) | 112 (41.3) | 119 (43.9) | 1.3 |
| Maharashtra | Sangli | 426 | 88 (20.7) | 156 (36.6) | 161 (37.8) | 243 | 61 (25.1) | 100 (41.2) | 106 (43.6) | 1.2 |

*Third serosurvey was done among individuals aged 10 years and above
