# Supplementary material for: Seroprevalence of IgG antibodies against SARS-CoV-2 among the general population and healthcare workers in India, June–July 2021: A population-based cross-sectional study
Source: PLoS Med. 2021 Dec 10;18(12):e1003877. doi: 10.1371/journal.pmed.1003877 (PMC8726494; doi:10.1371/journal.pmed.1003877)
Supplement: S5 Table — (DOCX) [file pmed.1003877.s008.docx]

**S5. Table: Estimated number of SARS-CoV-2 infections among individuals aged 6 years and above and infection to case ratio**

|  | Estimate (95% CI) by anti-N and/or anti-S - Seroprevalence considering the unvaccinated population* | Estimate (95% CI) by anti-N and/or S - Seroprevalence by considering the entire population** |
| --- | --- | --- |
|  |  |  |
| Estimated number of infections | 642,751,546  (625,901,252 – 659,601,839) | 807,395,611  (78,64,69,887 – 82,83,21,335) |
| Number of reported COVID-19 cases (9 Jun 2021) | 29,088,245 | 29,088,245 |
| Infection to Case ratio (9 Jun 2021) | 22.1 (21.5 - 22.7) | 27.8 (27.0 - 28.5) |
| Number of reported COVID-19 cases (16 Jun 2021) | 29,632,302 | 29,632,302 |
| Infection to Case ratio (16 Jun 2021) | 21.7 (21.1 - 22.3) | 27.2 (26.5 - 28.0) |

***** Applying the weighted seroprevalence of IgG antibodies against SARS-CoV-2 among individuals aged 6 -17 years to the population aged 6-17 years and the weighted seroprevalence of IgG antibodies against SARS-CoV-2 among unvaccinated individuals aged >= 18 years to the total population of **unvaccinated individuals** aged >=18 years

****** Applying the weighted seroprevalence of IgG antibodies against SARS-CoV-2 among individuals aged 6 -17 years to the population aged 6-17 years and the weighted seroprevalence of IgG antibodies against SARS-CoV-2 among unvaccinated individuals aged 18 years and above to **the total population** aged >=18 years
