# Supplementary material for: Seroprevalence of IgG antibodies against SARS-CoV-2 among the general population and healthcare workers in India, June–July 2021: A population-based cross-sectional study
Source: PLoS Med. 2021 Dec 10;18(12):e1003877. doi: 10.1371/journal.pmed.1003877 (PMC8726494; doi:10.1371/journal.pmed.1003877)
Supplement: S6 Table — (DOCX) [file pmed.1003877.s009.docx]

**S6. Table Comparison of seroprevalence of IgG antibodies against anti-N and/or anti-S protein of SARS-CoV-2 by demographic characteristics between third (Dec 2020-Jan 2021) and fourth (Jun-Jul 2021) serosurveys**

| Characteristics | Third serosurvey (Dec 2020-Jan 2021) Prevalence in % (95%CI) | Fourth serosurvey (Jun-Jul 2021)  Prevalence in % (95%CI) |
| --- | --- | --- |
| Age in years |  |  |
| 10-17 | 27.2 (24.9 - 29.4) | 61.6 (59.8 - 63.3) |
| 18-44 | 22.2 (21.1 - 23.4) | 66.7 (65.3 - 68.0) |
| 45-60 | 26.7 (25.2 - 28.2) | 77.6 (76.1 - 79.0) |
| >60 | 26.3 (24.3 - 28.3) | 76.7 (74.6 - 78.7) |
| Sex |  |  |
| Male | 23.2 (22.1 - 24.5) | 65.8 (64.4 - 67.1) |
| Female | 24.9 (23.7 - 26.3) | 69.2 (67.9 - 70.5) |
| Others | --- |  |
| Residence |  |  |
| Rural | 21.4 (20.3 - 22.6) | 66.7 (65.4 - 68.1) |
| Urban non-slum | 29.5 (27.0 - 32.1) | 69.1 (66.6 - 71.6) |
| Urban Slum | 34.7 (31.2 - 38.5) | 71.0 (66.8 - 74.7) |
| Overall | 24.1 (23.0–25.3) | 67.6 (66.4 - 68.7) |
