# Supplementary material for: Seroprevalence of IgG antibodies against SARS-CoV-2 among the general population and healthcare workers in India, June–July 2021: A population-based cross-sectional study
Source: PLoS Med. 2021 Dec 10;18(12):e1003877. doi: 10.1371/journal.pmed.1003877 (PMC8726494; doi:10.1371/journal.pmed.1003877)
Supplement: S7 Table — (DOCX) [file pmed.1003877.s010.docx]

| Characteristics | Refused/Not available, no. (% of total) | Participated in the survey, no. (% of total) | Total | P Value |
| --- | --- | --- | --- | --- |
| Age Group | **6586** | **28975** | **35561** |  |
| < 10 | 320 (4.9) | 2892 (10.0) | 3212 (9.0) | <0.001 |
| 10-17 | 652 (9.9) | 5798 (20.0) | 6450 (18.1) |  |
| 18 - 44 | 3604 (54.7) | 12522 (43.2) | 16126 (45.3) |  |
| 45 - 60 | 1535 (23.3) | 5545 (19.1) | 7080 (19.9) |  |
| 60+ | 475 (7.2) | 2218 (7.7) | 2693 (7.6) |  |
| Gender | **6583** | **28943** | **35526** |  |
| Male | 3680 (55.9) | 13783 (47.6) | 17463 (49.2) | <0.001 |
| Female | 2903 (44.1) | 15160 (52.4) | 18063 (50.8) |  |

**S7.Table: Characteristics of individuals who participated and those who didn’t participate in the survey**
