# Supplementary material for: Seroprevalence of IgG antibodies against SARS-CoV-2 among the general population and healthcare workers in India, June–July 2021: A population-based cross-sectional study
Source: PLoS Med. 2021 Dec 10;18(12):e1003877. doi: 10.1371/journal.pmed.1003877 (PMC8726494; doi:10.1371/journal.pmed.1003877)
Supplement: S1 Text — (DOCX) [file pmed.1003877.s011.docx]

**S1 Text: Data Analysis**

The survey was conducted in 70 randomly selected districts. From each selected district, 10 villages/wards were selected by probability proportional to size method. Design weights were computed by the inverse of product of probabilities at all stages of selection (i.e. selection of villages/wards within districts and households). The design weights were normalized and attached to the master dataset [1].

The binary coded outcome of seroprevalence (Positive/Negative) was obtained by considering individuals with IgG antibodies against Nucleocapsid and/or Spike protein of SARS-CoV-2 as seropositive and negative for both antibodies as seronegative. The logistic regression model for seroprevalence is given below

$$logit \left( \frac{{Prob(Y}_{ij}= 1)}{1-({Prob(Y}_{ij}= 1)} \right)= \beta_{0}+ \beta_{1}*X$$

Where

Y_ij_ = Seroprevalence (1 = Positive / 0 = Negative) i = Individuals, j = Villages/Wards

X = Independent variables (Gender, Age group, etc)

Generalized linear mixed-effects models with a logit link function was used to evaluate the seroprevalence of SARS-CoV-2 and other independent variables like age group, gender, area of residence etc., Random effects logistic regression model was used to address the clustering effect of estimates by considering villages/wards as the level. A random intercept model with design weights was used to estimate the overall seroprevalence.

$$logit \left( \frac{{Prob(Y}_{ij}= 1)}{1-({Prob(Y}_{ij}= 1)} \right)= \beta_{0j}+ \beta_{1}*X+ \vartheta_{j}$$

Seroprevalence estimates were obtained by exponentiating the log odds values obtained from the model and converting into probability and its corresponding 95% Wald confidence interval were obtained [1].

$Seroprevalence = \frac{\exp(logodds)}{(1+\exp(logodds))}$

The weighted seroprevalence was further adjusted for the joint sensitivity and specificity of the two assays using the sensitivities and specificities estimated by the manufacturer using the following formula [2].

$${Sensitivity}_{Joint}=P\left( {anti-N}^{+} \cup{anti-S}^{+} \right|{Covid19}^{+})$$

$$=Sens\left( {anti-N}^{+} \right)+ Sens\left( {anti-S}^{+} \right) - Sens\left( {anti-N}^{+} \right)*Sens\left( {anti-S}^{+} \right)$$

$${Specificity}_{Joint}=P\left( {anti-N}^{-} \cap{anti-S}^{-} \right|{Covid19}^{-})$$

$$= Spec\left( {anti-N}^{-} \right)*Sens\left( {anti-S}^{-} \right)$$

We additionally conducted a sensitivity analysis to estimate the seroprevalence by using the lowest sensitivity and specificity of the two assays estimated through the external validation studies as well as by considering the sensitivity and specificity estimated during in-house validation (given in the table below).

Reproduced from:

1. Murhekar MV, Bhatnagar T, Selvaraju S et al. SARS-CoV-2 antibody seroprevalence in India, August-September, 2020: findings from the second nationwide household serosurvey. Lancet Glob Health. 2021 Mar;9(3):e257-e266.
2. Branscum AJ, Gardner IA, Johnson WO. Estimation of diagnostic-test sensitivity and specificity through Bayesian modeling. Prev Vet Med. 2005 May 10;68(2-4):145-63. doi: 10.1016/j.prevetmed.2004.12.005. PMID: 15820113.

**Sensitivity and Specificity estimated by external studies and in-house validation of Abbott and Siemens SARS-CoV-2 IgG assay**

| **Source** | **Abbott assay** | | **Siemens assay** | |
| --- | --- | --- | --- | --- |
|  | **Sensitivity** | **Specificity** | **Sensitivity** | **Specificity** |
| Irsara C et al^1^ | 90.8% (86.3–93.9) | 99.3% (97.6–99.8) |  |  |
| Irsara C et al^2^ |  |  | 90.5% (85.2- 94.3) | 99.4% (96.6–100.0) |
| Tang MS^3^ | 93.8% (82.8-98.7) | 99.4% (96.4-99.9%) |  |  |
| Suhandynata RT^4^ | 92.60% | 100% |  |  |
| Theel ES^5^ | 95.70% | 99.60% |  |  |
| Padoan A^6^ | 95.2% (89.1-98.4) | 100.0% (93.4-100.0) |  |  |
| Manalac J^7^ | 97.90% | 99.60% |  |  |
| Hubbard JA^8^ | 91.3% (72.0-98.9) | 100% (99.05-100.00) |  |  |
| National SARS-CoV-2 Serology Assay Evaluation Group.^9^ | 92.7% (90.2 - 94.8) | 99.9% (99.4 - 100) |  |  |
| In house validation of Kits | 61.4% (52.8 - 69.5) | 100% (96.4 – 100) | 80.0% (72.4 – 86.3) | 99.0% (94.6 – 100.0) |

1. Irsara C, Egger A, Prokop W et al. Evaluation of four commercial, fully automated SARS-CoV-2 antibody tests suggests a revision of the Siemens SARS-CoV-2 IgG assay. Clinical Chemistry and Laboratory Medicine (CCLM). 2021;(). <https://doi.org/10.1515/cclm-2020-1758>.
2. Irsara C, Egger AE, Prokop W, Nairz M, Loacker L, Sahanic S, Pizzini A, Sonnweber T, Holzer B, Mayer W, Schennach H, Loeffler-Ragg J, Bellmann-Weiler R, Hartmann B, Tancevski I, Weiss G, Binder CJ, Anliker M, Griesmacher A, Hoermann G. Clinical validation of the Siemens quantitative SARS-CoV-2 spike IgG assay (sCOVG) reveals improved sensitivity and a good correlation with virus neutralization titers. Clin Chem Lab Med. 2021 Apr 9;59(8):1453-1462. doi: 10.1515/cclm-2021-0214. PMID: 33837679.
3. Tang MS, Hock KG, Logsdon NM, et al. Clinical Performance of Two SARS-CoV-2 Serologic Assays. Clin Chem. 2020;66(8):1055-1062. doi:10.1093/clinchem/hvaa120
4. Suhandynata RT, Hoffman MA, Kelner MJ, McLawhon RW, Reed SL, Fitzgerald RL. Multi-Platform Comparison of SARS-CoV-2 Serology Assays for the Detection of COVID-19. J Appl Lab Med. 2020 Nov 1;5(6):1324-1336. doi: 10.1093/jalm/jfaa139. PMID: 32766840; PMCID: PMC7454554.
5. E.S. Theel, J. Harring, H. Hilgart, D. Granger, Performance characteristics of four high-throughput immunoassays for detection of IgG antibodies against SARS-CoV-2, J. Clin. Microbiol. (2020), https://doi.org/10.1128/JCM.01243-20.
6. Padoan A, Bonfante F, Pagliari M, Bortolami A, Negrini D, Zuin S, Bozzato D, Cosma C, Sciacovelli L, Plebani M. Analytical and clinical performances of five immunoassays for the detection of SARS-CoV-2 antibodies in comparison with neutralization activity. EBioMedicine. 2020 Dec;62:103101. doi: 10.1016/j.ebiom.2020.103101. Epub 2020 Nov 4. PMID: 33160207; PMCID: PMC7640894.
7. Manalac J, Yee J, Calayag K, Nguyen L, Patel PM, Zhou D, Shi RZ. Evaluation of Abbott anti-SARS-CoV-2 CMIA IgG and Euroimmun ELISA IgG/IgA assays in a clinical lab. Clin Chim Acta. 2020 Nov;510:687-690. doi: 10.1016/j.cca.2020.09.002. Epub 2020 Sep 8. PMID: 32910980; PMCID: PMC7476889.
8. Hubbard JA, Geno KA, Khan J, Szczepiorkowski ZM, de Gijsel D, Ovalle AA, AlSalman AS, Gallagher TL, Johnston AA, Tibbetts AR, Vital SE, Cervinski MA, Nerenz RD. Comparison of Two Automated Immunoassays for the Detection of SARS-CoV-2 Nucleocapsid Antibodies. J Appl Lab Med. 2021 Mar 1;6(2):429-440. doi: 10.1093/jalm/jfaa175. PMID: 32976593; PMCID: PMC7543392.
9. National SARS-CoV-2 Serology Assay Evaluation Group. Performance characteristics of five immunoassays for SARS-CoV-2: a head-to-head benchmark comparison. Lancet Infect Dis. 2020 Dec;20(12):1390-1400. doi:10.1016/S1473-3099(20)30634-4. Epub 2020 Sep 23. Erratum in: Lancet Infect Dis. 2020 Dec;20(12):e298. PMID: 32979318; PMCID: PMC7511171.
