# Supplementary material for: Seroprevalence of IgG antibodies against SARS-CoV-2 among the general population and healthcare workers in India, June–July 2021: A population-based cross-sectional study
Source: PLoS Med. 2021 Dec 10;18(12):e1003877. doi: 10.1371/journal.pmed.1003877 (PMC8726494; doi:10.1371/journal.pmed.1003877)
Supplement: Attachment — Submitted filename: PMEDICINE-D-21-03454_R1_reviewer.pdf [file pmed.1003877.s012.pdf]

PLOS Medicine

Prevalence of IgG antibodies against SARS-CoV-2 among the general population and healthcare workers in India, June–July 2021

--Manuscript Draft--

Manuscript Number:	PMEDICINE-D-21-03454R1
Full Title:	Prevalence of IgG antibodies against SARS-CoV-2 among the general population and healthcare workers in India, June–July 2021
Short Title:	Seroprevalence of IgG antibodies against SARS-CoV-2, India, June-July 20201
Article Type:	Research Article
Abstract:	Background: India began COVID-19 vaccination in January 2021 initially targeting the healthcare and frontline workers. The vaccination strategy was expanded in a phased manner and currently covers all individuals aged 18 years and above. India witnessed a severe second wave of COVID-19 during March and June 2021. We did the fourth nationwide serosurvey to estimate prevalence of SARS-CoV-2 antibodies in the general population aged >=6 years and health care workers (HCWs).<br>Methods and findings: We did a cross-sectional study between 14 June and 6 July 2021 in 700 clusters in the same 70 districts across 21 states/Union Territory. From each district, a minimum of 400 individuals aged >=6 years from general population and 100 HCWs from the district public health facilities were included. The serum samples were tested for the presence of IgG antibodies against S1-RBD and nucleocapsid protein of SARS-CoV-2 using chemiluminescence immunoassay. We estimated the weighted and test adjusted seroprevalence of IgG antibodies against S1-RBD and/or nucleocapsid protein along with 95% CI.<br>Of the 28,975 sera tested, the weighted and test adjusted prevalence of IgG antibodies against S1-RBD and/or nucleocapsid protein among the general population aged >=6 years was 67.6% (95% CI: 66.4 – 68.7). The seroprevalence increased with age and was not different in rural and urban areas. Compared to unvaccinated adults (62.3%, 95% CI: 60.9 – 63.7), seroprevalence was significantly higher among individuals who received one (81.0%, 95% CI: 79.6 - 82.3) and two doses (89.8%, 95% CI: 88.4 - 91.1). The seroprevalence of IgG antibodies among 7,252 health care workers was 85.2% (95% CI: 83.5 - 86.7).<br>Our study has certain limitations. First, our serosurvey was designed to estimate the seroprevalence at the national level and it might not have captured variation in the seroprevalence within the states and districts. Second, approximately 19% of eligible individuals were not included in the survey, as they were not available in the household at the time of survey or they refused to participate. This could introduce a selection bias, if this non-response was not at random.<br>Conclusions: Nearly two of the three individuals aged ≥6 years from the general population and 85% HCWs had antibodies against SARS- CoV-2 by June 2020 in India. As one third of the population is still seronegative, it is necessary to accelerate the coverage of COVID-19 vaccination among adults. COVID-19 cases in India have been declining since May 2021. However, continued surveillance for COVID-19 cases is necessary to detect upsurge of COVID-19 cases early. The ongoing genomic surveillance for SARS-CoV- 2 also needs to be strengthened to inform about the emergence of newer variants including their ability to circumvent immunity conferred by the natural infection as well as vaccination. Finally, high seroprevalence observed in the general population should not be a reason for complacency. It is essential to continue adherence to non- pharmaceutical interventions, such as avoiding gatherings, ensuring social distancing, and using face masks in public places.
Additional Information:	
Question	Response
**Financial Disclosure**<br><br>Enter a financial disclosure statement that describes the sources of funding for the work included in this submission. Review	MVM received the funding from Indian Council of Medical Research, New Delhi. The funders were involved in study design, and the decision to publish and preparation of the manuscript.

Powered by Editorial Manager® and ProduXion Manager® from Aries Systems Corporation

<table>
<tr><td>

the submission guidelines for detailed requirements. View published research articles from *PLOS Medicine* for specific examples.

This statement is required for submission and **will appear in the published article** if the submission is accepted. Please make sure it is accurate.

Unfunded studies
Enter: *The author(s) received no specific funding for this work.*

Funded studies
Enter a statement with the following details:
• Initials of the authors who received each award
• Grant numbers awarded to each author
• The full name of each funder
• URL of each funder website
• Did the sponsors or funders play any role in the study design, data collection and analysis, decision to publish, or preparation of the manuscript?
• **NO** - Include this sentence at the end of your statement: *The funders had no role in study design, data collection and analysis, decision to publish, or preparation of the manuscript.*
• **YES** - Specify the role(s) played.

* typeset

</td><td></td></tr>
<tr><td>

Competing Interests

Use the instructions below to enter a competing interest statement for this submission. On behalf of all authors, disclose any competing interests that could be perceived to bias this work—acknowledging all financial support and any other relevant financial or non-financial competing interests.

This statement **will appear in the published article** if the submission is accepted. Please make sure it is accurate. View published research articles from *PLOS Medicine* for specific

</td><td>The authors have declared that no competing interests exist.</td></tr>
</table>

examples.<br><br>**NO authors have competing interests**<br><br>Enter: *The authors have declared that no competing interests exist.*<br><br>**Authors with competing interests**<br>Enter competing interest details beginning with this statement:<br><br>*I have read the journal's policy and the authors of this manuscript have the following competing interests: [insert competing interests here]*<br><br>* typeset<br><br>This statement is required for submission and **will appear in the published article** if the submission is accepted. Please make sure it is accurate and that any funding sources listed in your Funding Information later in the submission form are also declared in your Financial Disclosure statement.	
**Ethics Statement**<br><br>You must include an Ethics Statement in the Methods section of your manuscript if your study involved:<br>• Human participants<br>• Human specimens or tissue<br>• Vertebrate animals or cephalopods<br>• Vertebrate embryos or tissues<br>• Field research<br><br>**Not including a statement when required will delay the review process.**<br><br>General guidance is provided below. Please consult the guidelines on human subjects research and animal research for	I confirm that I have read and understood PLOS' requirements for an Ethics Statement.

detailed instructions.<br><br>**Format for specific study types**<br><br>**Human Subject Research (involving human participants and/or tissue)**<br>• Give the name of the institutional review board or ethics committee that approved the study<br>• Include the approval number and/or a statement indicating approval of this research<br>• Indicate the form of consent obtained (written/oral) or the reason that consent was not obtained (e.g. the data were analyzed anonymously)<br><br>**Animal Research (involving vertebrate animals, embryos or tissues)**<br>• Provide the name of the Institutional Animal Care and Use Committee (IACUC) or other relevant ethics board that reviewed the study protocol, and indicate whether they approved this research or granted a formal waiver of ethical approval<br>• Include an approval number if one was obtained<br>• If the study involved *non-human primates*, add *additional details* about animal welfare and steps taken to ameliorate suffering<br><br>**Field Research**<br><br>Include the following details if this study involves the collection of plant, animal, or other materials from a natural setting:<br>• Field permit number<br>• Name of the institution or relevant body that granted permission<br><br>Please check the box to confirm your understanding of this policy, then read and agree to one of the statements.	
Please respond:<br>as follow-up to "**Ethics Statement**	My study requires an ethics statement. I confirm I have included my statement in the Methods section of my uploaded manuscript file.

Powered by Editorial Manager® and ProduXion Manager® from Aries Systems Corporation

You must include an Ethics Statement in the Methods section of your manuscript if your study involved:

- Human participants
- Human specimens or tissue
- Vertebrate animals or cephalopods
- Vertebrate embryos or tissues
- Field research

Not including a statement when required will delay the review process.

General guidance is provided below. Please consult the guidelines on human subjects research and animal research for detailed instructions.

Powered by Editorial Manager® and ProduXion Manager® from Aries Systems Corporation

**Format for specific study types**<br><br>**Human Subject Research (involving human participants and/or tissue)**<br>• Give the name of the institutional review board or ethics committee that approved the study<br>• Include the approval number and/or a statement indicating approval of this research<br>• Indicate the form of consent obtained (written/oral) or the reason that consent was not obtained (e.g. the data were analyzed anonymously)<br><br>**Animal Research (involving vertebrate animals, embryos or tissues)**<br>• Provide the name of the Institutional Animal Care and Use Committee (IACUC) or other relevant ethics board that reviewed the study protocol, and indicate whether they approved this research or granted a formal waiver of ethical approval<br>• Include an approval number if one was obtained<br>• If the study involved *non-human primates*, add *additional details* about animal welfare and steps taken to ameliorate suffering<br><br>**Field Research**<br><br>Include the following details if this study involves the collection of plant, animal, or other materials from a natural setting:<br>• Field permit number<br>• Name of the institution or relevant body that granted permission<br><br>Please check the box to confirm your understanding of this policy, then read and agree to one of the statements. "	
**Data Availability**<br><br>Authors are required to make all data underlying the findings described fully available, without restriction, and from the time of publication. PLOS allows rare exceptions to address legal and ethical concerns. See the PLOS Data Policy and	Yes - all data are fully available without restriction

Powered by Editorial Manager® and ProduXion Manager® from Aries Systems Corporation

FAQ for detailed information.<br><br>A Data Availability Statement describing where the data can be found is required at submission. Your answers to this question constitute the Data Availability Statement and **will be published in the article**, if accepted.<br><br>**Important:** Stating 'data available on request from the author' is not sufficient. If your data are only available upon request, select 'No' for the first question and explain your exceptional situation in the text box.<br><br>Do the authors confirm that all data underlying the findings described in their manuscript are fully available without restriction?	
**Describe where the data may be found in full sentences. If you are copying our sample text, replace any instances of XXX with the appropriate details.**<br><br>• If the data are **held or will be held in a public repository**, include URLs, accession numbers or DOIs. If this information will only be available after acceptance, indicate this by ticking the box below. For example: *All XXX files are available from the XXX database (accession number(s) XXX, XXX.*).<br>• If the data are all contained **within the manuscript and/or Supporting Information files**, enter the following: *All relevant data are within the manuscript and its Supporting Information files.*<br>• If neither of these applies but you are able to provide **details of access elsewhere**, with or without limitations, please do so. For example:<br><br>*Data cannot be shared publicly because of [XXX]. Data are available from the XXX Institutional Data Access / Ethics Committee (contact via XXX) for*	All relevant data are within the manuscript and its Supporting Information files.

researchers who meet the criteria for access to confidential data.<br><br>*The data underlying the results presented in the study are available from (include the name of the third party and contact information or URL).*<br>• This text is appropriate if the data are owned by a third party and authors do not have permission to share the data.<br><br>* typeset	
Additional data availability information:	

Powered by Editorial Manager® and ProduXion Manager® from Aries Systems Corporation

Manuscript

Click here to access/download;Manuscript;SS4_Final manuscript.pdf

Prevalence of IgG antibodies against SARS-CoV-2 among the general population and healthcare workers in India, June–July 2021

Manoj V Murhekar[1], Tarun Bhatnagar[1], Jeromie Wesley Vivian Thangaraj[1], V Saravanakumar[1], Muthusamy Santhosh Kumar[1], Sriram Selvaraju[2], Kiran Rade[3], CP Girish Kumar[1], R Sabarinathan[1], Alka Turuk[4], Nivethitha N Krishnan[2], Aby Robinson[2], Nivetha Srinivasan[2], Smita Asthana[5*], Rakesh Balachandar[6*], Sampada Dipak Bangar[7*], Avi Kumar Bansal[8*], Jyothi Bhat[9*], Vishal Chopra[10*], Dasarathi Das[11*], Alok Kumar Deb[12*], Kangjam Rekha Devi[13*], Gaurav Raj Dwivedi[14*], S Muhammad Salim Khan[15*], M Sunil Kumar[16*], Avula Laxmaiah[17*], Major Madhukar[18*], Amarendra Mahapatra[11*], Chethana Rangaraju[19*], Jyotirmayee Turuk[11*], Suresh Yadav[20*], P K Anand[20†], Rushikesh Andhalkar[2†], Nimmathota Arlappa [17†], Khalid Bashir[15†], Dinesh Kumar Bharadwaj[17†], Pravin Bharti[9†], Debdutta Bhattacharya[11†], Sthita Pragnya Behera[14†], Ashrafjit S Chahal[10†], Debjit Chakraborty[12†], Anshuman Chaudhury[2†], Hirawati Deval[14†], Sarang Dhatrak[6†], Vikas Dhikav[20†], Rakesh Dayal[21†], Prathiksha Giridharan[2†], Inaamul Haq[15†], Babu Jagjeevan[17†], Agam Jain[22†], Arshad Kalliath[16†], Srikanta Kanungo[11†], T Karunakaran[1†], Jaya Singh Kshatri[11†], Niraj Kumar[14†], Vijay Kumar[5†], VG Vinoth Kumar[2†], G G J Naga Lakshmi[23†], Ganesh Mehta[8†], Anindya Mitra[21†], K Nagbhushanam[2†], A R Nirmala[24†], Subrata Kumar Palo[11†], Ganta Venkata Prasad[23†], Uday Kumar Pucha[17†], Mariya Amin Qurieshi[15†], Vikas Sabaharwal[22†], Seema Sahay[7†], Ramesh Kumar Sangwan[20†], Rochak Saxena[25†], Krithikaa Sekar[2†], Vijay Kumar Shukla[25†], Hari Bhan Singh[8†], Prashant Kumar Singh[5†], Pushpendra Singh[9†], Rajeev Singh[14†], Mahendra Thakor[20†], Dantuluri Sheethal Varma[23†], Ankit Viramgami[6†], Pradeep A Menon[2†], Rajiv Yadav[9*], Surabhi Yadav[2], Manjula Singh[4], Amit Chakrabarti[6#], Aparup Das[9#], Shanta Dutta[12#], Rajni Kant[14#], A M Khan[4#], Kanwar Narain[13#], Somashekar Narasimhaiah[19#], Chandrasekaran Padmapriyadarshini[2#], Krishna Pandey[18#], Sanghamitra Pati[11#], Hemalatha Rajkumar[17#], T Ramesh[23#], Arun Kumar Sharma[20#], YK Sharma[25#], Shalini Singh[5#], Samiran Panda[4], D C S Reddy[26], Balram Bhargava[4] on behalf of ICMR serosurveillance group

[1] ICMR National Institute of Epidemiology, Chennai, Tamil Nadu
[2] ICMR National Institute for Research in Tuberculosis, Chennai, Tamil Nadu
[3] WHO Country Office for India, New Delhi
[4] Indian Council of Medical Research, New Delhi
[5] ICMR- National Institute of Cancer Prevention & Research, NOIDA, Uttar Pradesh
[6] ICMR-National Institute of Occupational Health, Ahmedabad, Gujarat
[7] ICMR-National AIDS Research Institute, Pune, Maharashtra
[8] ICMR-National JALMA Institute for Leprosy & Other Mycobacterial Diseases, Agra, Uttar Pradesh
[9] ICMR-National Institute of Research in Tribal Health, Jabalpur, Madhya Pradesh
[10] State TB Training and Demonstration Centre, Patiala, Punjab
[11] ICMR- Regional Medical Research Centre, Bhubaneswar, Odisha
[12] ICMR- National Institute of Cholera and Enteric Diseases, Kolkata, West Bengal
[13] ICMR- Regional Medical Research Centre, N.E. Region, Dibrugarh, Assam
[14] ICMR- Regional Medical Research Centre, Gorakhpur, Uttar Pradesh
[15] Govt Medical College Srinagar, Srinagar, Jammu and Kashmir
[16] State TB Training & Demonstration Centre, Thiruvananthapuram, Kerala
[17] ICMR - National Institute of Nutrition, Hyderabad, Telangana
[18] ICMR-Rajendra Memorial Research Institute of Medical Sciences, Patna, Bihar
[19] National Tuberculosis Institute, Bangalore, Karnataka

[20]ICMR, National Institute for Implementation Research on Non-Communicable Diseases, Jodhpur, Rajasthan
[21] State TB Training and Demonstration Centre, Ranchi, Jharkhand
[22] NTEP Consultant, Uttarakhand
[23] State TB Office, Hyderabad, Andhra Pradesh
[24]Lady Willingdon State TB Centre, Bengaluru, Karnataka
[25] State TB Training and Demonstration Centre, Raipur, Chhattisgarh
[26] Independent consultant, Lucknow, Uttar Pradesh

[*]Authors in alphabetical order, contributed equally
[†]Authors in alphabetical order, contributed equally
[#]Authors in alphabetical order, contributed equally

Correspondence to:
Dr Manoj V Murhekar,
ICMR National Institute of Epidemiology,
Chennai 600077, India
mmurhekar@nieicmr.org.in

ICMR sero-surveillance group

Epidemiology and Surveillance Working Group: Tanu Anand, Giridhara R Babu, Himanshu Chauhan, Tanzin Dikid, Raman R Gangakhedkar, Shashi Kant, Sanket Kulkarni, J P Muliyil, Ravindra Mohan Pandey, Swarup Sarkar, Naman Shah, Aakash Shrivastava, Sujeet K Singh, Sanjay Zodpey

Laboratory and data management team: K Aparna, K Arul, PR Asish, M Chellakumar, D Chokkalingam, R Gunasekaran, Anusha Hindupur, S Kalaivani, K Kalaiyarasi, C Kanagasivam, N N Karthick, G Kiruthika, S Manickam, M Purushothaman, S Sarathkumar, M P Sarath Kumar, E Michaelraj, Josephine Pradhan, E B Arun Prasath, Sudha Rani, Amanda Rozario, R Sivakumar, P Gnana Soundari, K Sujeetha, P Tamilselvi, G Veeravel, Arya Vinod.

Abstract

Background: India began COVID-19 vaccination in January 2021 initially targeting the healthcare and frontline workers. The vaccination strategy was expanded in a phased manner and currently covers all individuals aged 18 years and above. India witnessed a severe second wave of COVID-19 during March and June 2021. We did the fourth nationwide serosurvey to estimate prevalence of SARS-CoV-2 antibodies in the general population aged >=6 years and health care workers (HCWs).

Methods and findings: We did a cross-sectional study between 14 June and 6 July 2021 in 700 clusters in the same 70 districts across 21 states/Union Territory. From each district, a minimum of 400 individuals aged >=6 years from general population and 100 HCWs from the district public health facilities were included. The serum samples were tested for the presence of IgG antibodies against S1-RBD and nucleocapsid protein of SARS-CoV-2 using chemiluminescence immunoassay. We estimated the weighted and test adjusted seroprevalence of IgG antibodies against S1-RBD and/or nucleocapsid protein along with 95% CI.

Of the 28,975 sera tested, the weighted and test adjusted prevalence of IgG antibodies against S1-RBD and/or nucleocapsid protein among the general population aged >=6 years was 67.6% (95% CI: 66.4 – 68.7). The seroprevalence increased with age and was not different in rural and urban areas. Compared to unvaccinated adults (62.3%, 95% CI: 60.9 – 63.7), seroprevalence was significantly higher among individuals who received one (81.0%, 95% CI: 79.6 - 82.3) and two doses (89.8%, 95% CI: 88.4 - 91.1). The seroprevalence of IgG antibodies among 7,252 health care workers was 85.2% (95% CI: 83.5 - 86.7).

Our study has certain limitations. First, our serosurvey was designed to estimate the seroprevalence at the national level and it might not have captured variation in the seroprevalence within the states and districts. Second, approximately 19% of eligible individuals were not included in the survey, as they were not available in the household at the time of survey or they refused to participate. This could introduce a selection bias, if this non-response was not at random.

Conclusions: Nearly two of the three individuals aged ≥6 years from the general population and 85% HCWs had antibodies against SARS- CoV-2 by June 2020 in India. As one third of the population is still seronegative, it is necessary to accelerate the coverage of COVID-19 vaccination among adults. COVID-19 cases in India have been declining since May 2021. However, continued surveillance for COVID-19 cases is necessary to detect upsurge of COVID-19 cases early. The ongoing genomic surveillance for SARS-CoV-2 also needs to be strengthened to inform about the emergence of newer variants including their ability to circumvent immunity conferred by the natural infection as well as vaccination. Finally, high seroprevalence observed in the general population should not be a reason for complacency. It is essential to continue adherence to non-pharmaceutical interventions, such as avoiding gatherings, ensuring social distancing, and using face masks in public places.

Introduction

With more than 30 million cases and 0.48 million deaths as on 5 July 2021, India has the second largest number of COVID-19 cases reported globally.[1] India witnessed a severe second wave of COVID-19 since March 2021, affecting all states of India.[2] Repeated cross sectional serosurveys in the same geographical location are useful to monitor the trends of seroprevalence over time and provide evidence for the public health decision making to plan the response.[3] Serial serosurveys conducted in 70 districts spread across 21 Indian states/Union Territory prior to introduction of COVID-19 vaccination indicated that the seroprevalence increased from 0.73% (95% CI: 0.34-1.13) during May-June 2020, to 6·6% (95% CI 5·8–7·4) during Sept-October 2020 and 24.1 (95%CI: 23.0% to 25.3%) during December 2020–January 2021.[4–6] About 25% healthcare workers working in peripheral health facilities in these 70 districts had evidence of IgG antibodies against SARS-CoV-2 during December 2020 – January 2021.[6] The earlier nationwide serosurveys in the general population were conducted among individuals aged 10 years and above while the information about the seroprevalence among children below 10 years is not known.

Since January 16, 2021 India initiated COVID-19 vaccination with BBV152 (Covaxin; Bharat Biotech International Ltd, Hyderabad) and ChAdOx1 nCoV-19 vaccine (Covishield, Serum Institute of India, Pune) for the healthcare and frontline workers. The vaccination strategy was expanded in a phased manner to cover individuals above 60 years old and above 45 years old with specified comorbidities (phase-2, March 1, 2021), all individuals aged 45 years and above (phase 3, April 1, 2021) and all individuals aged 18 years and above (phase 4, May 1, 2021).[7]

In this context, we conducted fourth round of nationwide serosurvey to estimate the age-specific prevalence of SARS-CoV-2 antibodies in the general population and health care workers.

Methods

Study design and participants:

We conducted a cross-sectional survey between 14 June and 6 July 2021 in the same 70 districts spread across 21 Indian states/Union Territory where three rounds of serosurveys were conducted[4–6]. We selected 10 clusters (Wards in urban areas and villages in rural areas) from each district using population proportion to size method. The survey teams selected four random starting points within each of the selected cluster. Starting from a random starting point, the teams visited consecutive households and listed all household members aged 6 years and above and who were permanently residing in the area. Eligible individuals present in the household were invited to participate in the survey. From each random location, at least 10 consenting individuals (1 aged 6-9 years, 2 aged 10-17 years and 7 aged 18 years and above) were enrolled in the survey. Enrolment of a minimum number of individuals in each age group ensured that the overall distribution of the sampled population was comparable to the age structure of the population in India.[8] Thus, a minimum of 40 individuals from each cluster and 400 individuals from each district were enrolled. We enrolled at least 100 healthcare workers from each of the 70 districts selected for the general population

survey. We consecutively enrolled the willing HCWs (Doctors, nurses, para medical staff and lab staff) working in the district headquarters hospital of the selected study district

Procedures

We interviewed eligible consenting participants to collect information about demographic details, history of symptoms suggestive of COVID-19 (eg, fever, cough, shortness of breath, sore throat, new loss of taste or smell, fatigue) since 1 January 2021, COVID-19 testing and COVID-19 vaccination. Three mL of venous blood was collected from each participant, and serum samples were transported to ICMR National Institute of Epidemiology, Chennai under cold chain.

We tested the serum samples for the presence of IgG antibodies against S1-RBD (ADVIA Centaur XP/XPT, Siemens Healthineers, Munich, Germany) and nucleocapsid (Abbott Architect, Abbott Park, IL, USA) protein of SARS-CoV-2 using chemiluminescence immunoassay, as per the manufacturer's instructions. The Siemens assay is a quantitative antibody assay with analytical measuring interval of 0.50 –150.0; samples with an index value of ≥1 are considered as reactive. The assay has sensitivity of 96.4% (95% CI: 92.7–98.5%) after 21 days of PCR confirmation of SARS-CoV-2 infection and specificity of 99.0% (95% CI: 99.6-99.9).[9] The Abbott assay for IgG antibodies against nucleocapsid protein is a qualitative assay and has a sensitivity of 100·0% and specificity of 99·6% after 14 days of PCR confirmation.[10] As a part of quality control, 10% of positive serum samples and an equal number of negative serum samples were re-tested using the same assay.

We also separately estimated the performance of the two assays by testing 100 pre-pandemic sera samples collected as a part of acute fever surveillance during 2016 and 140 samples from laboratory confirmed COVID-19 patients collected over 30-240 days after confirmation of RT-PCR.[11] We estimated the specificity of 99.0% (95% CI: 94.6–100.0%) and sensitivity of 80.0% (95% CI: 72.4–86.3%) for S1-RBD and specificity of 100% (95% CI: 96.4 – 100) and sensitivity of 61.4% (95% CI: 52.8 - 69.5) for nucleocapsid assay in detecting historical infection (supplementary material).

Data analysis

The characteristics of study participants was described as proportions. Individuals whose sera were positive for IgG antibodies against S1-RBD and/or nucleocapsid proteins were considered as seropositive. We calculated design weights as the product of inverse of sampling fraction for the selection of districts and selection of clusters within each district. We estimated the weighted seroprevalence of IgG antibodies along with 95% CI, using a random-effects model to account for cluster sampling. The weighted seroprevalence was further adjusted for the joint sensitivity and specificity of the two assays using the sensitivities and specificities estimated by the manufacturer.[12] We also conducted a sensitivity analysis to estimate the seroprevalence by using the lowest sensitivity and specificity of the two assays estimated through the external validation studies as well as by considering the sensitivity and specificity estimated during in-house validation (supplementary material). We also estimated the seroprevalence by selected demographic and COVID-19-related characteristics of the study participants.

To estimate the total number of individuals infected with SARS-CoV-2 at the national level, we applied the weighted seroprevalence of IgG antibodies against SARS-CoV-2 among children aged 6-17 years to the total population of children aged 6-17 years. To

estimate the total number of infections among individuals aged ≥18 years, we considered two scenarios: First, we applied the weighted seroprevalence among unvaccinated individuals aged 18 years and above to the total population of unvaccinated individuals aged >=18 years. Second, we applied the weighted seroprevalence among unvaccinated to the total population above 18 years. The infection to case ratio (ICR) was calculated by dividing the estimated number of SARS-CoV-2 infection with the cumulative number of COVID-19 cases reported in India 1 and 2 weeks before the median survey date (23 June 2020) assuming IgG antibodies start appearing between 5 and 15 days post infection.[13]

Protection of human participants: A written informed consent was taken from individuals aged ≥18 years. For children aged between 7 and 17 years, we obtained assent and written consent from their parents or guardians, while parental consent was obtained for children aged 6 years. The Institutional Human Ethics Committee of the ICMR National Institute of Epidemiology, Chennai approved the study protocol.

Results

Seroprevalence among general population

The survey teams visited 16, 074 households from 700 clusters in 70 Indian districts. Of the 35, 561 individuals aged ≥6 years residing in these households, 28, 975 (81.5%) consented to participated in the survey (Fig 1).

Of the 28,975 individuals who participated in the survey, 2892 (10%) were aged 6-9 years, 5798 (20%) were aged 10-17 years and 20,285 (70%) were aged ≥ 18 years. 15,160 (52.3%) participants were females and 21,794 (75.2%) resided in rural areas (Table 1). 4372 (15.1%) of the 28, 956 individuals reported history of COVID-19 testing in the past, of whom 782 (17.9%) reported a positive test result. Of the 20,268 adult participants, 5038 (24.8%) and 2631 (13.0%) reported receipt of one and two doses of COVID-19 vaccines respectively, while the remaining 12,599 (62.2%) were unvaccinated. Most (n=6945, 90.6%) had received Covishield vaccine (Table 1). The median interval between the receipt of the first dose and date of sample collection was 36 days (IQR: 14 - 68) and 60 days (IQR: 34 – 98) between the receipt of second dose and sample collection.

Of the 28,975 sera tested, 11, 289 (38.9%) had IgG antibodies against nucleocapsid protein, 18388 (63.5%) had antibodies against S1-RBD and 19336 (66.7%) had antibodies against nucleocapsid and/or S1-RBD. The weighted prevalence of IgG antibodies either against S1-RBD and/or nucleocapsid protein was 67.6% (95% CI: 66.4 – 68.7) after adjusting for assay characteristics (Table 2). The overall seropositivity in states ranged between 44.3% (Kerala) and 80.0% (Madhya Pradesh) (Supplementary material). The seroprevalence was heterogenous among the 70 districts, ranging between 40.5% (Ernakulam, Kerala) and 86.8% (Buxar, Bihar) (Supplementary material). Compared to December 2020, the seropositivity among unvaccinated individuals aged 10 years and above during June 2021 had increased by 1.2 - 1.9 folds in 21 (30.0%) districts, 2 – 3 folds in 31 (44.3%) districts and >3 folds in 18 (25.7%) districts (Supplementary material).

The seroprevalence showed a rising trend with age. Among children aged 6-9 years and 10-17 years, respectively 57.2% (95% CI: 55.0 - 59.4) and 61.6% (95% CI: 59.8 – 63.3)

had antibodies against SARS-CoV-2. The seroprevalence among adults ranged between 66.7% (95% CI: 65.3 - 68.0) in 18-44 years and 77.6% (95% CI: 76.1 - 79.0) in 45-60 years. The seroprevalence was not different in rural, urban non-slum and urban slum areas. Among the 487 unvaccinated individuals with history of laboratory confirmed COVID-19 infection, 88.0% (95% CI: 83.0–91.8) had detectable antibodies against SARS-CoV-2 (Table 3).

Compared to unvaccinated adults, the seroprevalence was significantly higher among individuals who received one (81.0%, 95% CI: 79.6 - 82.3, p=0.001) and two doses (89.8, 95% CI: 88.4 - 91.1, p=0.001) of COVID-19 vaccines. This increase was observed in all age-groups, both sexes and all localities among the individuals in general population as well as healthcare workers (Table 4 and supplementary material). The seroprevalence was higher among individuals who received Covishield (85.2%, 83.8 – 86.5) as compared to those who received Covaxin (80.2%, 76.1 – 83.8) (p=0.004). (Table 3). After one dose, the seroprevalence was 80.8% (95% CI: 75.7-85.0) for Covaxin and 82.0% (95% CI: 80.3-86.6) among Covishield recipients. Individuals who received two doses had higher seroprevalence, ranging between 86.3% to 90.3% for Covaxin and Covishield respectively (Table 4). A similar pattern was observed among healthcare workers (Supplementary material).

The weighted prevalence of IgG antibodies against nucleocapsid protein was 38.3 (95% CI: 37.0 - 39.5) (Table 2). The anti-N seropositivity was not different by age, gender and area of residence (Supplementary material). Also, around one third of the individuals vaccinated with Covishield had anti-N IgG antibodies.

Using the sensitivities and specificities estimated from the inhouse validation and external studies, the overall seroprevalence of IgG antibodies against SARS-CoV-2 was 73.2% (95% CI: 71.9 - 74.4) and 68.0% (95% CI: 66.8 - 69.1) respectively.

We estimated 642,751,546 to 807, 395,611 SARS-CoV-2 infections in India by mid-June 2021. With 2,90,88,245 and 2,96,32,302 cases reported by 9 June and 16 June 2021 respectively, the infections per reported COVID-19 case ranged between 21.7 and 27.2 (supplementary material).

Seroprevalence among healthcare workers

We enrolled 7,252 healthcare workers from the district public hospitals of the 70 districts selected for general population survey. Most (n=5133, 70.8%) HCWs were aged between 18-44 years and 51.3% (n=3722) were females. Of the 4892 (67.4%) HCWs who reported history of COVID-19 testing, 1354 (27.7%) had a positive test result. 89.5% (n=6493) had reported history of COVID-19 vaccination, while the remaining were unvaccinated (Table 1).

Of the 7252 healthcare workers, 6186 (85.3%) had antibodies against nucleocapsid and/or S1-RBD with the seroprevalence of 85.2 (83.5 - 86.7) after adjusting for assay characteristics (Table 2). The seroprevalence was not different by age groups and sex (Table 3). The seroprevalence was higher among HCWs who received one (87.7%, 95% CI: 85.0 – 89.9) and two doses (88.6%, 95% CI: 87.1 – 90.1) of COVID-19 vaccines as compared to unvaccinated individuals (64.8%, 95% CI: 60.1 – 69.2).

Discussion

The results from the fourth nationwide serosurvey indicate that about two-third of India's population aged 6 years and above had antibodies against SARS-CoV-2 by June 2021. The seroprevalence increased with age. The seroprevalence was comparable in rural, urban non-slum and urban slum areas. Majority of HCWs working in the district level health facilities were positive for IgG antibodies. These findings have important implications on the future trajectory of COVID-19 in India.

The overall prevalence of IgG antibodies against SARS-CoV-2 increased from 24.1% by December 2020 to 67.6% by June 2021. This increase in the seroprevalence could be on account of natural infection as well as COVID-19 vaccination. The seroprevalence among unvaccinated adults in June 2021 was 62.3% as against 24.3% in December 2020. This finding indicates that a large proportion of the increase in the seroprevalence was on account of the natural infection during the second wave of COVID-19 in India during March and June 2021. Around 38% of the individuals had anti-N antibodies indicating recent transmission of SARS-CoV-2.[14] During this period more than 20 million COVID-19 cases were reported from India,[2] with delta variant being a predominant circulating variant of concern (VOC).[15]

The increase in seroprevalence was observed in all age groups, including children aged 10-17 years (2.2 fold) (supplementary material). Children aged 6-9 years were not covered during the previous serosurveys. During June 2021, about 60% of children had evidence of antibodies against SARS-CoV-2. The findings of high seropositivity among children observed in our study are consistent with a recent study conducted during March and June 2021 among children from five sites in India .[16] Among the 700 individuals aged <18 years surveyed in this study, 55.7% were seropositive.

Earlier serosurveys have indicated higher seroprevalence among urban slums and urban non-slum areas than rural areas. This gradient in the seroprevalence seems to have faded by June 2021, with a comparable seroprevalence in the rural and urban areas. Higher seroprevalence in rural areas observed in our survey indicate that the infection in the second wave was widespread in rural areas.

The results of the Phase 2 trials reported that 98.4% (95% CI: 95.3 – 99.7) individuals vaccinated with BBV152 (Covaxin)[17] and 100 % (95% CI: 97.4 – 100.0) individuals vaccinated with Covishield[18] seroconverted 56 days after the second dose. An observational multi-centric study among health care workers in India reported a seropositivity of 98.1% among Covishield and 80% among Covaxin recipients.[19] In another study of antibody responses in a cohort of 45,965 adults from the general population of the United Kingdom who received either ChAdOx1 or the BNT162b2 SARS-CoV-2 vaccines, authors estimated that about 6% of the participants were 'low responders'.[20] In our serosurvey, about 10-14 % of the vaccinees were found to be seronegative even after the receipt of two doses of COVID-19 vaccine. The proportion of seronegative individuals did not change when we considered the optimal interval between the second dose and sample collection of more than 7 days. Because of cross-sectional nature of the study, we are not able to comment if the seronegativity among the fully vaccinated individuals was on account of lower antibody response or decline in antibodies. Moreover, the possibility of misclassification of vaccination status cannot be ruled out as the information about vaccination was based on recall.

The number of infections per reported case between December 2020 (27.1- 26.7) and June 2021 (21.7 – 27.8) has not changed. This reflects on sustained testing (around 210 million tests conducted during this period) of both symptomatic and asymptomatic eligible individuals.[6]

Seroprevalence studies can help predict the future course of the pandemic.[3] Prior to the second wave in India, about 75% of the population was seronegative.[6] The serosurvey findings indicate that about one third of the general population in India did not have detectable antibodies against SARS-CoV-2 by June 2021. It is therefore possible that more COVID-19 cases are likely to occur in coming months, especially in areas where the proportion of people without detectable antibodies is higher. The available evidence indicates that immunity acquired through natural infection can last up to one year.[21] IgG antibodies against the RBD of the spike protein shows a high correlation to virus neutralization titres indicating the neutralising nature of the antibodies.[22,23] Studies also indicate that re-infections among previously infected individuals are less frequent.[24] It is thus reasonable to expect that the future surge of cases in India would be lower than the second wave. However, the immunity acquired through natural infection as well as vaccination is expected to wane over time. Although S1-RBD are considered to have the neutralising effect, protective titre among the seropositive individuals is not known. Further, COVID-19 cases could rapidly increase after emergence of immune escape variants.[25] It is therefore necessary to continue monitoring the emergence of VOCs.

Our study has certain limitations. First, our serosurvey was designed to estimate the seroprevalence at the national level and it might not have captured variation in the seroprevalence within the states and districts. Second, approximately 19% of eligible individuals were not included in the survey, as they were not available in the household at the time of survey or they refused to participate. This could introduce a selection bias, if this non-response was not at random. Third, IgG antibodies against SARS-CoV-2 wane over time.[14,26] In our study, the seropositivity to S1-RBD and nucleocapsid protein among unvaccinated individuals with laboratory confirmed COVID-19 RT-PCR was 82.4% and 63.4% respectively. Hence, the observed seroprevalence might be under-estimate of the actual seroprevalence in the population.

In conclusion, our serosurvey findings indicate that nearly two of the three individuals aged ≥6 years from the general population and 85% HCWs had antibodies against SARS-CoV-2 by June 2020 in India. As one third of the population is still seronegative, it is necessary to accelerate the coverage of COVID-19 vaccination among adults. COVID-19 cases in India have been declining since May 2021. However, continued surveillance for COVID-19 cases is necessary to detect upsurge of COVID-19 cases early. The ongoing genomic surveillance for SARS-CoV-2 also needs to be strengthened to inform about the emergence of newer variants including their ability to circumvent immunity conferred by the natural infection as well as vaccination. Finally, high seroprevalence observed in the general population should not be a reason for complacency. It is essential to continue adherence to non-pharmaceutical interventions, such as avoiding gatherings, ensuring social distancing, and using face masks in public places.

Authors contributions

MVM, TB, JWVT, MSaK, KR, DCSR, SP and BB designed the study. SSe, AT, NNK, AR, NS MVM, MSaK, JWVT did project management. MSK, JWVT, RSa, SA, RB, SDB, AKB, JB, VC, DD, AKD, KRD, GRD, SMSK, MSuK, AL, MM, AMa, CR, JT, SY, PKA,RA, NA, KB, DKB, PB, DB, SPB, ASC, DC, AC, HD, SD, VD, RD, PG, IH, BJ, AJ, AK,SK, JSK, NK, VK, VGVK, GGJN, GM, KN, ARN, SKP, AKP, GVP, UKP, MAQ, VSab, SSa, RKS, RS, KS, VKS, HBS, PKS, PS, RSi, MT,DSV,AV, PAM, RY, SY, MS, AC, AD, SD, RK, AMK, KN, SN, CP, KP, SaP, HR, TR, AKS, YKS and ShS coordinated the blood sample and data collection. CPGK with the support of TK coordinated the laboratory processing and testing of samples. VS, JWVT, MVM, RSa and MSaK performed data analysis. MVM, TB, JWVT, MSaK, KR, SP, DCSR and BB performed data interpretation. MVM, TB, VS, JWVT, RSa and MSaK accessed and verified the data. MVM, TB, MSaK and JWVT wrote the first draft of the manuscript. All authors approved the final version of the manuscript.

Declaration of interests

We declare no competing interests.

Data sharing

A subset of the key anonymised individual participant data collected during the study, along with a data dictionary, is available upon request to the corresponding author, after approval of a proposal with a signed data access agreement.

References

1 World Health Organization. WHO Coronavirus (COVID-19) Dashboard. 2021; published online May 7. https://covid19.who.int/table (accessed July 28, 2021).

2 Government of India. COVID-19 Dashboard. 2021; published online May 7. https://www.mygov.in/covid-19.

3 World Health Organization. Population-based age-stratified seroepidemiological investigation protocol for coronavirus 2019 (COVID-19) infection, 26 May 2020. Geneva: World Health Organization, 2020 https://apps.who.int/iris/handle/10665/332188.

4 Murhekar MV, Bhatnagar T, Selvaraju S, *et al.* Prevalence of SARS-CoV-2 infection in India: Findings from the national serosurvey, May-June 2020. *Indian J Med Res* 2020; **152**: 48–60.

5 Murhekar MV, Bhatnagar T, Selvaraju S, *et al.* SARS-CoV-2 antibody seroprevalence in India, August–September, 2020: findings from the second nationwide household serosurvey. *The Lancet Global Health* 2021; **9**: e257–66.

6 Murhekar MV, Bhatnagar T, Thangaraj JWV, *et al.* SARS-CoV-2 seroprevalence among the general population and healthcare workers in India, December 2020–January 2021. *International Journal of Infectious Diseases* 2021; **108**: 145–55.

7 Ministry of Health and Family Welfare. COVID-19 Vaccine FAQs. https://www.mohfw.gov.in/covid_vaccination/vaccination/faqs.html (accessed July 29, 2021).

8 Census of India Website : Office of the Registrar General & Census Commissioner, India. C-13 Single year age returns by residence and sex. https://censusindia.gov.in/2011census/C-series/C-13.html (accessed July 29, 2021).

9 Siemens Healthineers. SARS-CoV-2 IgG Assay. https://www.siemens-healthineers.com/laboratory-diagnostics/assays-by-diseases-conditions/infectious-disease-assays/cov2g-assay (accessed July 29, 2021).

10 Health C for D and R. EUA Authorized Serology Test Performance. *FDA* 2021; published online May 25. https://www.fda.gov/medical-devices/coronavirus-disease-2019-covid-19-emergency-use-authorizations-medical-devices/eua-authorized-serology-test-performance (accessed July 29, 2021).

11 Thangaraj JWV, Kumar MS, Kumar CG, *et al.* Persistence of humoral immune response to SARS-CoV-2 up to 7 months post-infection: Cross-sectional study, South India, 2020-21. *J Infect* 2021; : S0163-4453(21)00268-1.

12 Sempos CT, Tian L. Adjusting Coronavirus Prevalence Estimates for Laboratory Test Kit Error. *American Journal of Epidemiology* 2021; **190**: 109–15.

13 Sette A, Crotty S. Adaptive immunity to SARS-CoV-2 and COVID-19. *Cell* 2021; **184**: 861–80.

14 Iyer AS, Jones FK, Nodoushani A, *et al.* Persistence and decay of human antibody responses to the receptor binding domain of SARS-CoV-2 spike protein in COVID-19 patients. *Sci Immunol* 2020; **5**. DOI:10.1126/sciimmunol.abe0367.

15 Dhar MS, Marwal R, Radhakrishnan VS, *et al.* Genomic characterization and Epidemiology of an emerging SARS-CoV-2 variant in Delhi, India. *medRxiv* 2021; : 2021.06.02.21258076.

16 Misra P, Kant S, Guleria R, Rai SK, Aiims WUS study team of. Serological prevalence of SARS-CoV-2 antibody among children and young age (between age 2-17 years) group in India: An interim result from a large multi-centric population-based seroepidemiological study. *medRxiv* 2021; : 2021.06.15.21258880.

17 Ella R, Reddy S, Jogdand H, *et al.* Safety and immunogenicity of an inactivated SARS-CoV-2 vaccine, BBV152: interim results from a double-blind, randomised,

multicentre, phase 2 trial, and 3-month follow-up of a double-blind, randomised phase 1 trial. *The Lancet Infectious Diseases* 2021; **21**: 950–61.
18 Marathe S. Covishield-ChAdOx1 nCoV-19 Corona Virus Vaccine Insert, Domestic - New 02012021.cdr. ; : 2.
19 Kumar Singh A, Phatak SR, Singh R, *et al.* Antibody Response after Second-dose of ChAdOx1-nCOV (Covishield™) and BBV-152 (Covaxin™) among Health Care Workers in India: Final Results of Cross-sectional Coronavirus Vaccine-induced Antibody Titre (COVAT) study. Allergy and Immunology, 2021 DOI:10.1101/2021.06.02.21258242.
20 Wei J, Stoesser N, Matthews PC, *et al.* Antibody responses to SARS-CoV-2 vaccines in 45,965 adults from the general population of the United Kingdom. *Nat Microbiol* 2021; : 1–10.
21 Turner JS, Kim W, Kalaidina E, *et al.* SARS-CoV-2 infection induces long-lived bone marrow plasma cells in humans. *Nature* 2021; **595**: 421–5.
22 Wajnberg A, Amanat F, Firpo A, *et al.* Robust neutralizing antibodies to SARS-CoV-2 infection persist for months. *Science* 2020; **370**: 1227–30.
23 Irsara C, Egger AE, Prokop W, *et al.* Clinical validation of the Siemens quantitative SARS-CoV-2 spike IgG assay (sCOVG) reveals improved sensitivity and a good correlation with virus neutralization titers. *Clinical Chemistry and Laboratory Medicine (CCLM)* 2021; **59**: 1453–62.
24 Hall VJ, Foulkes S, Charlett A, *et al.* SARS-CoV-2 infection rates of antibody-positive compared with antibody-negative health-care workers in England: a large, multicentre, prospective cohort study (SIREN). *The Lancet* 2021; **397**: 1459–69.
25 Prévost J, Finzi A. The great escape? SARS-CoV-2 variants evading neutralizing responses. *Cell Host Microbe* 2021; **29**: 322–4.
26 Bolotin S, Tran V, Osman S, *et al.* SARS-CoV-2 Seroprevalence Survey Estimates Are Affected by Anti-Nucleocapsid Antibody Decline. *J Infect Dis* 2021; **223**: 1334–8.

Acknowledgements

The authors acknowledge the field supervision and support provided by WHO India; the Ministry of Health and Family Welfare, Government of India; state and district health officials; and primary healthcare staff in the planning and conduct of the serosurvey. The authors thank the following team members from different Indian states.

Assam

Doirai Basumatary, Liladhar Boro, Jumanjyoti Sarmah, Anjan Saikia, Anu Chouhan, Dhurbajyoti Pathak, Nagen Sarmah, Tapan Hazarika, Nandeswar Hazarika, Arup Kr. Deka, Joydhon Timung, Suprotin Khatoniar, Upashna Bora, Dondodhar Gogoi, Thomas Langthasa, Thaneswar Teron, Bijoybhusan Deka, Sibsankar Chakaraboty, Pranjal Nath Ranjit Das, Bibek Saikia, Ritwik Dutta, Dipti Kangkana Kachari, Amir Sohail Khan, Mrinal Nagtey, Rimpi Konwar, Jasuda Chetia, Gunin Mili, Boidujya Rai Gogoi, Bidya Pegu, Ananta Borah, Gamuk Kuttum, Ranjali Doley, Agastin Kerketta, Santanu kakoty, Pullab Das, Prahlad Das, Paramananda Upadhaya, Krishna Khadka, Pankaj Phukan, Pranjal Das, Samir Ranjan Borah, Rituraj Buragohain, Tapan Kalita, Mrinmoy Gogoi, Amit Kr. Ray, Diganta Karmakar and Mahanta Gogoi

Bihar

Naveen Kumar Mandal, Kumar Gautam, Sanjeev Gupta, Ujjwal Prakash, Sahdeo Mandal, Prakash Ranjan, Saurav Kumar, Santosh Kumar, Rohit Kumar, Sashi Bhusan Awasthi, Mritunjay Kumar Pandey, Paras Kumar, Kumar Rakesh Mandal, Satish Kumar Thakur, Subhash Kumar, Manohar Kumar, Amit Lakra, Ashish Kumar, Binod Kumar, Rishikesh Kumar, Prateek Raushan, Vikas Kumar, Saurabh Krishna, Baidyanath Roy, Alok Kumar, Santosh Singh, Sakaldeep Kumar, Ajeet Kumar, Aaditya Panday, Umesh Kumar, Dhirendra Kumar, Amar Kant Singh, Sanjeet Kumar, Bhoop Dhakar, Vikash Kumar Roy, Kundan Kunal, Vikash Kumar, Kumar Abhijeet, Amrendra Kumar, Vikram Kumar Chaudhary, Mohammad Arif, Raneeta Singh, Madhu Kumari, Roushan Kumar, Parul Priya, Pooja Kumari, Manoj Kumar, B.P. Subramanya, Ujjwal Prasad Sinha, Rajeev Chandra Kumar, Ashish Tigga, Sanjay Kumar Singh, Geetika Shankar, Anand Gautam, Susheel Gautam, Rajendra Kumar, Adarsh Varghese, Anisur Rehman Bhuiyan and Kunnal Kuvalekar.

Chhattisgarh

Archana Nagwanshi, Sunil Kumar Pankaj, Irshad Khan, Chetan Ravishankar Raut, Kunjbihari Patel, Chandra Kishore Thakre, Pekhan Kumar Sahu, Nand Kumar Modi, Nand Kumar Sahu, Bhoopendra Thakur, Awadh Baghel, Hemant Bawthande, Dev Kumar Sahu, Rajesh Kumar Soni, Devdas Joshi and Vokesh Yadu

Gujarat

Dinkar Raval, SK Makwana, A.M. Kadri, Harsh Bakshi, Pranav Patel, Arthur Mcwan, Anand Santoke, Pankaj Nimawat, Shabbir Ali Dedhrotiya, YK Jani, Jitendra Patel, Hasmukh Parmar, Hardik Nakshiwala, Vaidehi Gohil, Jaydip Oza, Vikas Kokare, Mihir Rupani, Ankit Sheth, Parulben Patel, Jigneshbhai Tadvi, Priyank Gandhi, Piyushbhai Parasar, Vinodbhai Valvi, Jagdishbhai Padvi, Dhawal Patel, Divyaben Zala, Mayurbhai Vasava, Manmitbhai Solanki, Darshnaben Patel, Chetnaben Chaudhari, Aartiben Rathva,

Riyaben Mistry, Nikiben Bhau, Jyotsnaben Bariya, Tejasbhai Patel, Kartikbhai Prajapati, Babita Roy, Pareshbhai Parmar, Manojbhai Bhagora, Pareshbhai Patel, Hemantbhai Kalasva, Shardaben Vankar, Divya Patel, Ravisinh Chauhan, Nimisha Patel, Misha Patel, Harsha Sadat, Puja Patel, Girish Shah, Partapsinh Taviyad, Raginiben Gosai, Krutikaben Rana, Imtiyazbhai Shaikh, Madhuben Mahera, Bhavikaben Patel, Prakashakumar Patel, Sangitaben Patel, Geetaben Patel, Pratapbhai Pagi, Bharatbhai Rana, Jinalben Patel, Archanaben Pandavi, Dilipbhai Baria, Ishavar Sinh Rathod, Sharmishtha Patel, Sunitaben Solanki, Bhavesh Vaghela, Moinuddin Mansuri, Nitesh Rathore, Purvi Nayak, Hardeep Khair, Rajendra Acharya, Vijyaben Amin, Nirmal Prajapati, V.J. Pargi, Asmitaben Kharadi, Rajubhai Patel, Komalben, Hemangini Baria, Meenaben Bamaniya, Shantaben Prajapati, Rameshbhai Patel, Imran Mansuri, Yashvantbhai Nayak, K.K. Parmar, Rahul Siroi, Krunal Darji, Mahavir Solanki, Shivani Joshi and Mahesh Gavit.

Jammu and Kashmir

Haseena Mir, Syed Arshad Rafiq, Iram Sabah, Misbah Ferooz Kawoosa , Abdul Aziz Lone, Ishtiyaq Ahmad Sumji, Mehvish Afzal Khan, Shaista Ismail, Anjum Asma, Shifana Ayoub, Javed Ahmad Bhat, Shafin Ashraf wani, Asima Nazir, Mohd. Ashraf Bhat, Irfana Gani, Neelofar Akram, Riyaz A Wani, Manzoor A Magray, Gulban Fayaz, Parsa Shafi, Aasim Abubakar, Aymen Deeba, Aasim Maqbool, Firdous Mushtaq Khan, Mir Waleed, Inam Zahoor, Nimrat ul Ain Banday, Kaisar Hamid Malla, Saika Parvaiz, Wajahat Nazir Shazia Akhtar, Nazir Ahmed Bhat, Mushtaq Ahmad Bhat, Jehangi Ahmad Bhat, Naseeb Singh Bali, Ferooz Ahmad, Altaf Ahmad, Abdul Rashid Dar, Farooq Ahmad Magray, Manzoor ahmad, Gh Mustafa Bhat, Feroz Ahmad Bhat.

Jharkhand

Asit Mansingh, Amiya Ranjan Mohanta, Anjan Kumar Bishoyee, Trilochan Bhoi, Matrujyoti Pattnaik, Santosh Kumar Sahoo, Rajesh Kumar Panda, Ashok Kumar Mahakud, Debashish Mishra, Partha Sarathi Patra, Dasrath Majhi, Santosh Kumar Behuria, Jeevan Kumar Mohanto, Biren Kumar Padhy, Chanan Kumar Majhi, Krushna Chandra Dalei, Soumya Ranjan Panda

Karnataka

Jawaid Akhtar, K V Thrilok Chandra, Arundathi Chandrashekar, Patil Om Prakash, Rameshchandra Reddy,N Udanesha, Sarika Jain Agrawal, H P Arundathi Das, Ranganath R, Vivekanand Reddy,Mahesh Kumar, Bilagumba Ramaiah Venkateshaiah, Prathibha Javare Gowda, Ambaraya Rudrawadi, Basavaraj Biradar, Anand PrakashK R Nischith, G Hamsaveni, Satish Ghatage, R S Sreedhar, Mishba Hani, Anil Talikoti, N T Nagraja, , Pranesh Katti, Ratnasheela, Qayyum, Sangamesh,Shridhar, Amrut, Sankar Gouda, Maruthi Kamble, Anitha Jadhav, Deepak Patil, Amaraya Rudrawadi, S K Ravikanti,G Abhay Kumar, Ratnakar Toran, Suresh Doddamani, Suresh Mekin ,Deepak Patil, Siddaramappa Patil, Avinash Khaske, Abdul Jabbar, Shashidhar Patanaik, Amaresh Kolur, Asha,Shobha Nagesh, Ramya R, Babu Mahendra, Kusuma S, Roopashree, Malathi Y S, Bharath,Latha, Sudha,Shivakumar, G, Srinivas, Sadiqulla Sharif, Jagadeesh, Dilip,Vijay, Mallikarjunappa, Kavitha, Sudha, Premasudha, Chandrashekhar Kambalimat, Girish, Jayashimha, Venkatesh, Ananda Prakash, Shree Harsha, Vijayalakshmi N, Somashekharayya S Hiremath, K Jaya Ganesh, H S Charan Raj Rao, T.N.Basavaraju, B.Sampath, T.G.Gangadharaiah, H T Mangalagowri, Nagaraja P C, Ravikumara M T, Srikantha Y G, Hariprashanth R, Praveen Pujar, Bheema Zakeer

Hussain, Namewar Hanmanth, Kokila Manickam, Gowri K, Shadul Fakirsab Sayyad, Lal Kumar R, Ullera Ashoka, A N Sunil, Umar Farooque M Dalawai, Manjunatha M, Venkatesh A Millanatti, B.Dinesh Kumar, Narasimharaju N, Deepak, Sundara Murthi.R.

Kerala

Aravind Krishnan, Manoj M, Anumol Raju, Anupranam M P, Arima A R, Soniya Joseph, Shilna A, Vishnu Raj, Prakash Jaison V, Venoth V S, Gladson J, Ganga G S

Maharashtra

Ankita Dahiwade, Shabana Khan, Swati Salunkhe, Seema Nair, Shudhanshu Rane, Kanad Patil, Suresh Kumar, Savita Bahekar, Rakesh Gaikwad, Pravin Dandge, Namrata Hajari, Anil Rathod, Shaikh Shahrukh, Dinesh Jadhav, Pooja Pawara, Sonal Gawai, Priyanka Bawane, Vinod Pethkar, Dhiraj Panpatil, Ajit Buchude, Sourabh Tidake, Sagar Rokade, Bhuddhabhushan Bhadke, Kapil Khandagale, Chaitanya Deshmukh, Adinath Rokde, Suraj Rakhunde, Prathamesh Chavan, Pramod Jamale, Rahul Arke, Vivek Yengade, Tejas Phale, Amarkumar Ambade, N. Ramaswami, Satish Pawar, Archana Patil, Pradeep Awate, Jyoti Gurav, Abhijeet Raut, Deepak Mugalikar, Vipin Itankar, Rajendra Bhosale, Ravindra Jagtap, Abhijeet Chaudhari, Rajesh Deshmukh, Nagurao S. Chavan, Bhimashankar Jamadar, Nilkanth Bhosikar, Balaji Shinde, Suryankant Sable, Radhakrishna Pawar, Sunil Pokhrna, Sandip Sangle, Sanjay Salunkhe, Milind Pore, Balasaheb Nagargoje, Shankararo Deshmukh, Sandip Bharaswadkar, Aniruddha Kadu, Rajiv Kumar, Hemant Kharnare, Suhasini Kadhe, Avinash Jadhav, Chetan Khade, Amol Gaikwad, Virendra Wankhede, Prakash Nandapurkar, Jyoti Salve

Madhya Pradesh

M.P. Sharma, Shivendra Mishra, Mahavir Khandelwal, Sunita Parmar, Devendra Gothwal, Manish Sharma, Seema Jaiswal, B.K. Tiwari, Arvind Verma, Ajay Goel, Purushottam Patel Ganesh Damor, , Bhagwansingh Patil, Ramswaroop Uikey, , Akanksha Kushram, Sandip Sharma, Himmat Singh, Yogendra Mourya, Prahlad Soni, Pushpendra Rajput, Priyanka Birha, Monu Sen, Rekha Prajapati, Priyanka Singore, Lipi Jain, Ashok Solanki, Kalpana Patel, Bhagat Dhurvey, Rahul Choubey, Pehal Singh Tekam, Rajendra Mehra, Ramcharit Rakesh Tiwari, Prathinesh Parouha, Rahul Belawi, Yagendra Kumar, Amit Raikwar and Twinkle Dewaker

Odisha

AsitMansingh, Shakti Ranjan Barik, Siba Prasad Mallik, Prashant Majhi, Deepak Sahoo, ParthaSarathi Patra, A.K. Bishoyee, Hitesh Kumar Jain, Indurani Sagar, Santosh Kumar Sahoo, Rajesh Panda, Santosh Kumar Beuria, Sidhartha Kar, Soumya Panda, K. Sahu, MatrujyotiPattnaik, Dasarath Majhi, Biren Kumar Padhi, Padmamohan Pradhan and Arun Padhi.

Rajasthan

Rajkumar Kalunda, Rajneesh Kumar, Anil Purohit, Chet Ram Meena, Pankaj Kumar, Trilok Kumar, Bhanwar Manohar, Mohit Yadav, Jograj Singh, Ayub Khan, Ram Naresh Jani

Tamil Nadu

Y. John Arokyadoss, P. Kumaravel, A. Vasudevan, Magesh Kumar, J. Chitra, Santhana Kumar, Sadham Hussain, Kuppusamy Chandrabalu, Chandra Kumar, Selvam Suresh, Nandha Kumar, Dhanagopal Rajmohan, G, Thanappan Selvendran, Suryanarayanan Santhosh, Annadurai Arjun, N. Kumaravel, Vinoth

Telangana

R. Ananthan, Dudekula Anwar Basha, Blessy Prabhu Priyanka Salavadi, ChandraKumar Dolla, S. Devindra, Gargi Meur, Indrapal.I. Meshram, Paras Sharma, P. Raghavendra, Santosh Kumar Banjara, J. Ch. Sairam, Srinivas Rao, F. Sylvia, , MV Surekha, N.Samarasimha Reddy, Tata Aruna Kumari, V.Aruna, Ch. Anitha, N. Anjaiah, G.Bhavani, Ch. Bujji, Hrusikesh Panda, G. Hari Babu, N. Jhansi, S.V.J.Mohan Naidu, G.Neeraja, B.V. Nancharamma, D.Narasimhulu, P.Nagulu, Sk.Nasarvali, S.P.V. Prasad, B. Praveen, Ch.Pallavi, R.Raghunadh Babu, R.Rajyalaxmi, G.Venkat Raji Reddy, D.Rani, J.Raju Naik, V.Ravi, Ch.Sai Babu, G.L.Stephen, K. Sree Ramakrishna, PV. Sunu, P. Sreenu, D.Swaroopa, E. Sheela, M.Srinivas, P.Sathaiah, M.Suresh, P.Sriram, K.Sridhar, B. Srikant, R.Satyanarayanan, V.Sai Santosh, G.Tulasi, T.Usha Rani, K.Venkata Ramana, P. Venkatamma, D.Vasundhara and G. Vijaya Lakshmi.

Uttar Pradesh- West

Prabhat kumar, Dilip Singh, Naresh Dhakar, Rahul Kumar, Akash Yadav, Arpita Chaturvedi, Swati Singh, Brijesh Maurya, Manisha Dhakar, Narendra Singh Yadav, Sheena Singh, Renu Kanwar, Sonu Yadav, Rahul Yadav, Mewa kumar, Himalaya Kumar, Raju Deen Dayal, Balijeet Sodhi, Rajesh Jain, Shivanka Gaur, Akhil Tondon, Deepak Ohari, Saubhagya Prakash, Amit kumar, Haridutt Nemi

Uttar Pradesh- Central/Himachal Pradesh & Uttarakhand

Dechen Yangdol, Upendra Singh, Amit Yadav, Mohit Tiwari, Gopal Prasad, Sapna Yadav, Basudev Singh, Deepak Babu, Rahul Kumar, Chakrapani Katara, Chandra Pratap Singh, Simran Kaur Bhojwani, Manish Kumar, P. Vedival, Ranjan Karmakar, Vivek Kumar, Rahul Gond, Prabhat Kumar, Hariom Kushwah, Gani Aftridi, Nistha Verma, Veer Vishal, Rakesh Sharma, Uday Singh, Saurav Yadav, Navneet Rajput, Satya Prakash, Sunny Sharma, Santosh Kushwah, Akhalesh Yadav, Papai Das, Mahavir Singh Chaudhary, Prabha Shakya, Gulshan Sahu, Kumari Dipika, Sushil Chander, Manoj Sharma, Satyvrat Vaidya, Archana Srivastava, Shishir Puri, Vishal Agnihotri, Ashish Dixit, Ashutosh Kumar, Jairam Singh, Lalit Kumar, Tanmay Kakkar, Misaam Abbas, Akhileshwar Singh, Ashish Nautiyal, Manoj Kumar, Sushil Kumar, Shailendra Barthwal, Prakash Tapriyal, Subodh Kumar, Sanjay Pandey, Namita Puri, Arti Behal, V.C Kala, Ghambir Taliyan, Gaurav Raturi, Rashi Ranjan Kukreti, Pankaj Juyal, Kuldeep Kumar, Ashok Kumar, Dhani Ram, Prem Kumar, Loknath Sharma, Suraj Mani, Raj Kumari, Jitendra Kumar, Kiran Chauhan, Prakash Tapriyal, Vineet Kumar Shukla, J.S. Rawat, Himanshu Sharma, Madhu Kumari, Rajesh Mourya, R.S. Yadav, Surinder Singh, Raju Kumavat, Sandeep Patil, Pradhumn Katara, Namrata Soni, Prashant Upadhyaya, Praveen Pachauri, Ajay Rawat, Sanjay Chopra, Jyoti Mishra, Mohammed Husain, Devi Lal

Uttar Pradesh- East

Amit Mohan Prasad, Shruti Pandey, Markandey Shahi, Amit S Bansal, Atul Kumar Singhal, Sushil Kumar, Radhey Shyam Kesari, Ajay Pratap Singh, Vinay Dange, Ghanshyam Singh, Shri Prakash Agrawal, Shyam Narayan Dubey, Birendra Panchal, Vishal Yadav, Mukesh Kumar Mishra, Ravi Shankar Singh, Kamlesh Sah, Sonal Rajput, Sushil Pal, Ravi Nishad, Rohit Baghel, Punit Kumar, Abhishek Kumar Mishra, Avdhesh Kumar, Anugunj Chaudhary, Pawan Kumar, Surabhi Kushwaha, Mohammed Afroz, Lal Chand, Nisha Yadav, Anil Kushwaha, Deepak Kumar, Vipul Kumar, Kiran Kumari, Akash Kushwaha, Vinod Kumar, Ram Poojan Yadav and Santosh Kumar.

West Bengal

Falguni Debnath, Agniva Majumder, Gargi Dutta Bhattacharyya, Subrata Biswas, Ajay Chakraborty, Jayesh Mehta, Bandita Sengupta, Abhijit Dey, Arup Chakrabortty, Subhendu Kumar Ray, Shourav Tarak, Subhadip Bhunia, Debasish Roy, Shyamal Soren, Jagannath Sarkar, Somnath Mukherjee, Prakash Chandra Mridha, Girish Chandra Bera, Bibhas Roy, Santanu Sahu, Atrayee Chakraborty, Rabiul Islam Gayen, Dilip Biswas, Samudra Sengupta, Barnaman Tudu, Tanmoy Kumar Ghosh, Ananya Biswas, Poulami Sen, Sonali Das, Tridib Das, Tarun Khatua, Paritosh Mondal, Saptarshi Bannerjee, Amit Baran Barat, Arindam Sarkar, Soumen Jana, Joyeeta Bhattacharyya, Medhavi Manish, Biswajit Namasharma, Chandan Ghosh, Debarati Chakraborty, Kunal Maiti, Milan Barman, Pintu Manik, Purnima Roy, Rajani Kurmi, Rocky Ansari, Sanglap Maity, Somobrota Naskar, Sourav Pradhan, Bishakha Pramanik, Dipannita Sardar, Sujit Kumar Shreshta, Arpita Das, Ujjal Maitra, Nawaid Ali, Chandan Ghosh, Susanta Bera, Sk. Monirul Jaman, Dev Kumar Dolai, Purnima Das, Wasim Reza, Shrikant Shankar Gawali and Rajesh Das.

Figure: Participant enrolment

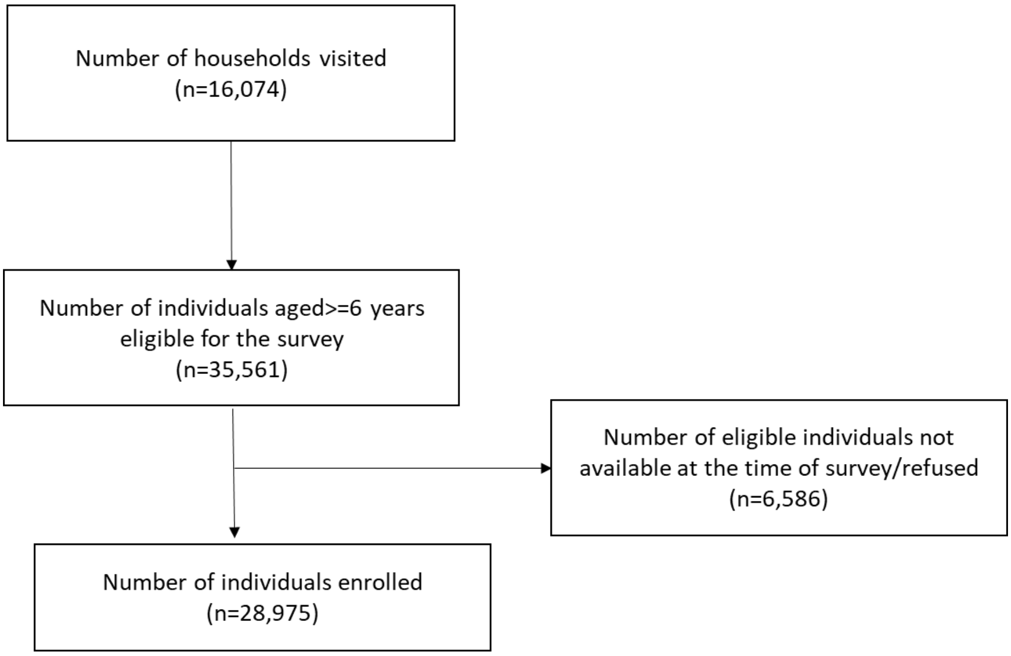

Table 1: Participant characteristics

Characteristics	General population n (%)	Healthcare workers n (%)
**Age (in years)**	**n= 28975**	**n=7252**
6-9	2892 (10.0)	-
10-17	5798 (20.0)	-
18-44	12522 (43.2)	5133 (70.8)
45-60	5545 (19.1)	1997 (27.5)
>60	2218 (7.7)	122 (1.7)
**Sex**	**n= 28975**	**n=7252**
Male	13783 (47.6)	3523 (48.6)
Female	15160 (52.3)	3722 (51.3)
Other	32 (0.1)	7 (0.1)
**Area of residence**	**n=28975**	
Rural	21794 (75.2)	-
Urban non-slum	5266 (18.2)	-
Urban slum	1915 (6.6)	
**COVID-19 related symptoms**	**n= 28975**	**n=7252**
History of COVID-19 symptoms since January 2021	1748 (6.0)	925 (12.8)
	**n=1729**	**n=925**
Medical care sought for symptomatic cases	574 (33.2)	558 (60.3)
History of hospitalization	140 (24.4)	171 (30.6)
	**n=28956**	**n=7252**
History of contact with a known COVID-19 case	2129 (7.3)	4562 (62.9)
	**n=28956**	**n=7252**
**Previously tested for COVID-19**	4372 (15.1)	4892 (67.4)
**Previous positive COVID-19 test**	782 (17.9)	1354 (27.7)
**COVID-19 vaccination status among adults**	**n=20268**	**n=7252**
0 dose	12599 (62.2)	759 (10.5)
1 dose	5038 (24.8)	972 (13.4)
2 doses	2631 (13.0)	5521 (76.1)
**Type of Vaccine**	**n=7669**	**n=6493**
Covaxin	587 (7.7)	498 (7.7)
Covishield	6945 (90.6)	5973 (92.0)
Sputnik	18 (0.2)	6 (0.1)
Don’t know	119 (1.6)	16 (0.2)

Table 2: Seroprevalence (%) of immunoglobulin G antibodies against severe acute respiratory syndrome coronavirus-2 infection, India, June-July 2021

	General population aged ≥ 6 years			Healthcare workers		
	Anti-N antibodies	Anti-S1-RBD antibodies	Anti-N and/or anti-S antibodies	Anti-N antibodies	Anti-S1-RBD antibodies	Anti-N and/or anti-S antibodies
Number of individuals tested	28975	28975	28975	7252	7252	7252
Number of positives	11289	18388	19336	2305	6112	6186
Unweighted prevalence * (%) 95% CI	38.9 (37.9 - 40.1)	63.5 (62.3 - 64.6)	66.7 (65.6 - 67.8)	31.8 (29.7 - 34.0)	84.3 (82.5 - 85.9)	85.3 (83.6 - 86.8)
Weighted prevalence** (%) 95% CI	38.5 (37.3 - 39.7)	64.4 (63.2 -65.6)	66.6 (65.3 - 67.9)	-	-	-
Adjusted prevalence*** (%) 95% CI	38.3 (37.0 - 39.5)	66.8 (65.5 - 68.0)	67.6 (66.4 - 68.7)	31.5 (29.4 - 33.7)	87.4 (85.6 - 89.1)	85.2 (83.5 - 86.7)

*Adjusted for clustering
**Weighted for design weights
***Adjusted for test performance

Table 3: Seroprevalence of IgG antibodies by selected characteristics, June-July 2021

Characteristics	General population			Healthcare workers		
	No. tested	No. positive (anti-N and/or anti-S1-RBD antibodies)	Weighted and test-performance-adjusted seroprevalence % (95% CI)	No. tested	No. positive (anti-N and/or anti-S1-RBD antibodies)	Test-performance-adjusted seroprevalence % (95% CI)
**Age, years**						
6-9	2892	1635	57.2 (55.0 - 59.4)	-	-	-
10-17	5798	3584	61.6 (59.8 - 63.3)	-	-	-
18-44	12522	8245	66.7 (65.3 - 68.0)	5133	4401	86.5 (84.9 - 88.0)
45-60	5545	4217	77.6 (76.1 - 79.0)	1997	1686	85.1 (83.0 - 87.1)
>60	2218	1655	76.7 (74.6 - 78.7)	122	99	80.4 (71.9 - 86.8)
**Sex**						
Male	13783	9018	65.8 (64.4 - 67.1)	3523	3024	86.2 (84.4 - 87.8)
Female	15160	10295	69.2 (67.9 - 70.5)	3722	3157	85.9 (84.1 - 87.6)
Other	32	23	83.4 (59.1 - 94.6)	7	5	66.4 (26.9 - 91.3)
**Area of residence**						
Rural	21794	14398	66.7 (65.4 - 68.1)	-	-	-
Urban non-slum	5266	3587	69.1 (66.6 - 71.6)	-	-	-
Urban slum	1915	1351	71.0 (66.8 - 74.7)	-	-	-
**History of COVID-19 related symptoms since 1 Jan 2021**						
Yes	1748	1262	76.8 (74.4 - 79.0)	925	838	85.2 (83.6 - 86.8)
No	27227	18074	66.9 (65.7 - 68.1)	6327	5348	91.5 (89.2 - 93.2)
**Previously tested for COVID-19**						
Yes	4372	3196	78.7 (77.1 - 80.2)	4892	4194	86.9 (85.2 - 88.4)
No	24584	16127	65.6 (64.4 - 66.9)	2360	1992	84.2 (81.9 - 86.3)
**Previous COVID-19 test result**						
Reported positive for COVID-19	782	674	88.9 (86.8 - 90.8)	1354	1275	94.8 (93.4 - 96.0)
Reported Negative for COVID-19	3419	2425	75.2 (73.2 - 77.0)	3395	2789	83.3 (81.1 - 85.3)

Don't know	171	97	71.1 (59.2 - 80.5)	143	130	90.1 (80.6 - 95.2)
**COVID-19 vaccination status among adults**						
0 dose	12599	7758	62.3 (60.9 - 63.7)	759	507	64.8 (60.1 - 69.2)
1 dose	5038	4016	81.0 (79.6 - 82.3)	972	834	87.7 (85.0 - 89.9)
2 doses	2631	2331	89.8 (88.4 - 91.1)	5521	4845	88.6 (87.1 - 90.1)
**Optimal interval between vaccination and blood sample collection**						
Less than 21 days of 1st dose	1711	1242	73.5 (70.6 - 76.2)	191	151	78.0 (70.7 - 83.9)
21 days after 1st dose	3327	2774	85.9 (84.3 - 87.4)	781	683	89.8 (87.2 - 92.1)
7 days after 2nd dose	2630	2330	90.4 (88.9 - 91.7)	5513	4837	88.6 (87.1 - 90.1)
**Vaccine type**						
Covaxin	587	473	80.2 (76.1 – 83.8)	498	428	86.5 (82.7 – 89.5)
Covishield	6945	5751	85.2 (83.8 – 86.5)	5973	5229	88.6 (87.0 – 90.1)
**Previously positive for COVID-19**						
0 dose	487	402	88.0 (83.0 - 91.8)	140	116	83.6 (76.0 - 89.2)
1 dose	145	134	95.0 (90.6 - 97.4)	154	146	95.2 (90.4 - 97.7)
2 doses	150	138	94.0 (88.2 - 97.1)	1060	1013	96.1 (94.4 - 97.4)

CI, confidence interval; COVID-19, coronavirus disease 2019; N, nucleocapsid protein; S1-RBD, spike protein of the receptor binding domain

Table 4: Seroprevalence of select characteristics by vaccination status, General population, June – July 2021

Characteristics	Unvaccinated			Vaccinated with one dose			Vaccinated with two doses		
	Tested	Positives N/S	Cluster and test adjusted Seroprevalence % (95% CI)	Tested	Positives N/S	Cluster and test adjusted Seroprevalence % (95% CI)	Tested	Positives N/S	Cluster and test adjusted Seroprevalence % (95% CI)
**Age, years**									
18-44	8986	5381	60.8 (59.2 - 62.3)	2426	1896	80.4 (78.2 - 82.4)	1096	958	89.1 (86.7 - 91.3)
45-60	2701	1778	67.0 (64.8 - 69.1)	1830	1521	85.5 (83.4 - 87.3)	1013	917	91.9 (89.7 - 93.6)
>60	912	599	67.0 (63.6 - 70.4)	782	599	78.9 (75.4 - 82.0)	522	456	88.7 (85.4 - 91.5)
**Sex**									
Male	10040	5962	59.6 (57.8 - 61.4)	2598	2050	81.0 (79.0 - 82.9)	1136	999	89.4 (87.1 - 91.5)
Female	11223	6998	64.9 (63.2 - 66.4)	2436	1962	83.1 (81.1 - 84.9)	1493	1330	90.7 (88.6 - 92.3)
Other	26	17	74.0 (44.9 - 90.8)	4	4	-	2	2	-
**Area of residence**									
Rural	3702	2280	62.1 (60.5 - 63.7)	1046	846	81.1 (79.2 - 82.9)	515	459	90.1 (88.0 - 91.7)
Urban non-slum	16236	9832	63.6 (60.2 - 66.8)	3603	2840	83.2 (79.8 - 86.1)	1941	1716	90.4 (86.9 - 93.0)
Urban slum	1351	865	64.6 (59.0 - 69.8)	389	330	87.1 (82.1 - 90.9)	175	156	90.9 (84.7 - 94.7)
**Previous COVID-19 test result**									
Reported positive for COVID-19	487	402	86.0 (82.2 - 89.1)	145	134	94.7 (90.1 - 97.2)	150	138	93.8 (88.6 - 96.7)
Reported Negative for COVID-19	1732	1051	62.9 (59.7 - 66.1)	962	739	80.1 (76.9 - 83.0)	724	634	89.4 (86.5 - 91.8)
Don't know	119	58	60.2 (46.5 - 72.5)	36	28	84.1 (67.5 - 93.1)	16	11	66.7 (37.4 - 87.0)
**Vaccine type**									
Covaxin	-	-	-	385	302	80.8 (75.7 - 85.0)	202	171	86.3 (80.4 - 90.8)
Covishield	-	-	-	4565	3636	82.0 (80.3 - 83.6)	2380	2115	90.3 (88.5 - 91.8)
**Optimal interval between vaccination and blood sample collection*** (21 days after 1st dose, 7 days after 2nd dose)									
Covaxin	-	-	-	223	186	84.3 (78.4 - 88.9)	201	170	86.3 (80.3 - 90.7)
Covishield	-	-	-	3045	2535	84.8 (83.1 - 86.5)	2380	2115	90.3 (88.5 - 91.8)

Supplementary Material

Contents Page

1. Statistical Analysis..........2

2. Sensitivity and Specificity estimated by external studies and in-house validation of Abbott and Siemens assay 3

3. Cluster adjusted proportion of individuals with SARS-CoV-2 IgG antibodies by states, fourth national SARS-CoV-2 serosurvey, India, June-July 2021..........5

4. Unweighted proportion of individuals with SARS-CoV-2 IgG antibodies by districts, fourth national SARS-CoV-2 serosurvey June-July2021..........6

5. Unweighted proportion of individuals with SARS-CoV-2 IgG antibodies among unvaccinated and aged above 10 years by districts, third and fourth national SARS-CoV-2 serosurvey, India 9

6. Seroprevalence of selected characteristics by vaccination status, Healthcare workers, June – July 2021..........13

7. Seroprevalence of IgG antibodies against Nucleocapsid by selected characteristics, June-July 2021..........14

8. Estimated number of infections among individuals aged 6 years above and infection case ratio..........15

9. Comparison of seroprevalence against N/S-antibody by demographic characteristics between third and fourth serosurvey 16

1. Statistical Analysis

The survey was conducted in 70 randomly selected districts. From each selected district, 10 villages/wards were selected by probability proportional to size method. Design weights were computed by the inverse of product of probabilities at all stages of selection (i.e. selection of villages/wards within districts and households). The design weights were normalized and attached to the master dataset.

Random effects logistic regression model was used to address the clustering effect of estimates by considering district as the level. A random intercept model with design weights was used to estimate the overall seroprevalence. Further, seroprevalence estimates for other factors like age, gender was estimated using the same model. Seroprevalence estimates were obtained by exponentiating the log odds values obtained from the model and converting into probability and its corresponding 95% Wald confidence interval were obtained.

Reproduced from:

Murhekar MV, Bhatnagar T, Selvaraju S et al. SARS-CoV-2 antibody seroprevalence in India, August-September, 2020: findings from the second nationwide household serosurvey. Lancet Glob Health. 2021 Mar;9(3):e257-e266.

2. **Sensitivity and Specificity estimated by external studies and in-house validation of Abbott and Siemens assay**

Source	Abbott assay		Siemens assay	
	Sensitivity	Specificity	Sensitivity	Specificity
Irsara C et al[1]	90.8% (86.3–93.9)	99.3% (97.6–99.8)		
Irsara C et al[2]			90.5% (85.2–94.3)	99.4% (96.6–100.0)
Tang MS[3]	93.8% (82.8-98.7)	99.4% (96.4-99.9%)		
Suhandynata RT[4]	92.60%	100%		
Theel ES[5]	95.70%	99.60%		
Padoan A[6]	95.2% (89.1-98.4)	100.0% (93.4-100.0)		
Manalac J[7]	97.90%	99.60%		
Hubbard JA[8]	91.3% (72.0-98.9)	100% (99.05-100.00)		
National SARS-CoV-2 Serology Assay Evaluation Group.[9]	92.7% (90.2 - 94.8)	99.9% (99.4 - 100)		
In house validation of Kits	61.4% (52.8 - 69.5)	100% (96.4 – 100)	80.0% (72.4 – 86.3)	99.0% (94.6 – 100.0)

1. Irsara C, Egger A, Prokop W et al. Evaluation of four commercial, fully automated SARS-CoV-2 antibody tests suggests a revision of the Siemens SARS-CoV-2 IgG assay. Clinical Chemistry and Laboratory Medicine (CCLM). 2021;(). https://doi.org/10.1515/cclm-2020-1758.
2. Irsara C, Egger AE, Prokop W, Nairz M, Loacker L, Sahanic S, Pizzini A, Sonnweber T, Holzer B, Mayer W, Schennach H, Loeffler-Ragg J, Bellmann-Weiler R, Hartmann B, Tancevski I, Weiss G, Binder CJ, Anliker M, Griesmacher A, Hoermann G. Clinical validation of the Siemens quantitative SARS-CoV-2 spike IgG assay (sCOVG) reveals improved sensitivity and a good correlation with virus neutralization titers. Clin Chem Lab Med. 2021 Apr 9;59(8):1453-1462. doi: 10.1515/cclm-2021-0214. PMID: 33837679.
3. Tang MS, Hock KG, Logsdon NM, et al. Clinical Performance of Two SARS-CoV-2 Serologic Assays. Clin Chem. 2020;66(8):1055-1062. doi:10.1093/clinchem/hvaa120
4. Suhandynata RT, Hoffman MA, Kelner MJ, McLawhon RW, Reed SL, Fitzgerald RL. Multi-Platform Comparison of SARS-CoV-2 Serology Assays for the Detection of COVID-19. J Appl Lab Med. 2020 Nov 1;5(6):1324-1336. doi: 10.1093/jalm/jfaa139. PMID: 32766840; PMCID: PMC7454554.
5. E.S. Theel, J. Harring, H. Hilgart, D. Granger, Performance characteristics of four high-throughput immunoassays for detection of IgG antibodies against SARS-CoV-2, J. Clin. Microbiol. (2020), https://doi.org/10.1128/JCM.01243-20.
6. Padoan A, Bonfante F, Pagliari M, Bortolami A, Negrini D, Zuin S, Bozzato D, Cosma C, Sciacovelli L, Plebani M. Analytical and clinical performances of five immunoassays for the detection of SARS-CoV-2 antibodies in comparison with neutralization activity. EBioMedicine. 2020 Dec;62:103101. doi: 10.1016/j.ebiom.2020.103101. Epub 2020 Nov 4. PMID: 33160207; PMCID: PMC7640894.
7. Manalac J, Yee J, Calayag K, Nguyen L, Patel PM, Zhou D, Shi RZ. Evaluation of Abbott anti-SARS-CoV-2 CMIA IgG and Euroimmun ELISA IgG/IgA assays in a clinical lab. Clin Chim Acta. 2020 Nov;510:687-690. doi: 10.1016/j.cca.2020.09.002. Epub 2020 Sep 8. PMID: 32910980; PMCID: PMC7476889.
8. Hubbard JA, Geno KA, Khan J, Szczepiorkowski ZM, de Gijsel D, Ovalle AA, AlSalman AS, Gallagher TL, Johnston AA, Tibbetts AR, Vital SE, Cervinski MA, Nerenz RD. Comparison of Two Automated Immunoassays for the Detection of SARS-CoV-2 Nucleocapsid Antibodies. J Appl Lab Med. 2021 Mar 1;6(2):429-440. doi: 10.1093/jalm/jfaa175. PMID: 32976593; PMCID: PMC7543392.
9. National SARS-CoV-2 Serology Assay Evaluation Group. Performance characteristics of five immunoassays for SARS-CoV-2: a head-to-head benchmark comparison. Lancet Infect Dis. 2020 Dec;20(12):1390-1400. doi: 10.1016/S1473-3099(20)30634-4. Epub 2020 Sep 23. Erratum in: Lancet Infect Dis. 2020 Dec;20(12):e298. PMID: 32979318; PMCID: PMC7511171.

3. **Cluster adjusted proportion of individuals with SARS-CoV-2 IgG antibodies by states, fourth national SARS-CoV-2 serosurvey, India, June-July 2021**

State/Union Territory	Total Tested	Positive for either anti-N or anti-S	Cluster Adjusted Seroprevalence % (95% CI)
Madhya Pradesh	1229	683	80.0 (76.3 - 83.2)
Rajasthan	1226	628	77.1 (73.2 - 80.7)
Bihar	2461	1174	76.7 (73.9 - 79.3)
Gujarat	1219	540	76.4 (72.4 - 80.1)
Chhattisgarh	1198	509	75.7 (71.6 - 79.4)
Uttarakhand	401	202	74.2 (66.7 - 80.6)
Uttar Pradesh	3733	1848	71.7 (69.3 - 74.1)
Andhra Pradesh	1260	462	71.2 (66.8 - 75.2)
Karnataka	1326	535	70.3 (65.9 - 74.4)
Tamil Nadu	1258	524	70.1 (65.6 - 74.2)
Odisha	1230	437	68.9 (64.3 - 73.1)
Punjab	1581	597	67.2 (63.2 - 71.0)
Telangana	1373	512	63.5 (58.8 - 67.9)
Jammu & Kashmir	430	124	63.4 (55.0 - 71.0)
Himachal Pradesh	400	82	62.5 (54.0 - 70.3)
Jharkhand	1231	415	61.9 (57.1 - 66.6)
West Bengal	2042	529	61.3 (57.6 - 65.0)
Haryana	398	137	60.4 (51.8 - 68.4)
Maharashtra	2468	785	58.4 (54.9 - 61.8)
Assam	1203	285	50.3 (45.3 - 55.3)
Kerala	1308	281	44.3 (39.5 - 49.2)

4. **Unweighted proportion of individuals with SARS-CoV-2 IgG antibodies by districts, fourth national SARS-CoV-2 serosurvey, India, June-July 2021**

District	State	Total Tested	N Positive (%)	S Positive (%)	N/S Positive (%)
Buxar	Bihar	400	216 (54.0)	335 (83.8)	347 (86.8)
Gwalior	Madhya Pradesh	416	219 (52.6)	330 (79.3)	340 (81.7)
Dewas	Madhya Pradesh	407	230 (56.5)	319 (78.4)	330 (81.1)
Vizianagaram	Andhra Pradesh	434	196 (45.2)	340 (78.3)	346 (79.7)
Surguja	Chhattisgarh	397	176 (44.3)	301 (75.8)	316 (79.6)
Chennai	Tamil Nadu	415	184 (44.3)	320 (77.1)	330 (79.5)
Madhubani	Bihar	424	213 (50.2)	327 (77.1)	337 (79.5)
Jalor	Rajasthan	409	207 (50.6)	310 (75.8)	323 (79.0)
Sabar Kantha	Gujarat	402	183 (45.5)	303 (75.4)	313 (77.9)
Arwal	Bihar	410	202 (49.3)	302 (73.7)	317 (77.3)
Balrampur	Uttar Pradesh	431	192 (44.5)	317 (73.5)	331 (76.8)
Rajsamand	Rajasthan	410	215 (52.4)	294 (71.7)	308 (75.1)
Narmada	Gujarat	398	160 (40.2)	289 (72.6)	297 (74.6)
Dausa	Rajasthan	407	206 (50.6)	280 (68.8)	303 (74.4)
Begusarai	Bihar	417	186 (44.6)	303 (72.7)	310 (74.3)
Ujjain	Madhya Pradesh	406	234 (57.6)	286 (70.4)	301 (74.1)
Mau	Uttar Pradesh	455	245 (53.8)	328 (72.1)	337 (74.1)
Saharanpur	Uttar Pradesh	399	220 (55.1)	275 (68.9)	295 (73.9)
Mahisagar	Gujarat	419	197 (47.0)	294 (70.2)	308 (73.5)
Ludhiana	Punjab	394	158 (40.1)	279 (70.8)	288 (73.1)
Garhwal	Uttarakhand	401	202 (50.4)	273 (68.1)	293 (73.1)
Unnao	Uttar Pradesh	395	202 (51.1)	270 (68.4)	288 (72.9)

District	State	Total Tested	N Positive (%)	S Positive (%)	N/S Positive (%)
Auraiya	Uttar Pradesh	398	234 (58.8)	259 (65.1)	290 (72.9)
Kabeerdham	Chhattisgarh	403	192 (47.6)	267 (66.3)	293 (72.7)
Ganjam	Odisha	419	154 (36.8)	297 (70.9)	304 (72.6)
Bangalore	Karnataka	447	165 (36.9)	313 (70.0)	323 (72.3)
Bijapur	Chhattisgarh	398	141 (35.4)	272 (68.3)	285 (71.6)
Bareilly	Uttar Pradesh	400	185 (46.3)	261 (65.3)	284 (71.0)
Gautam Buddha Nagar	Uttar Pradesh	398	186 (46.7)	265 (66.6)	281 (70.6)
Muzaffarpur	Bihar	404	194 (48.0)	264 (65.3)	281 (69.6)
Tiruvannamalai	Tamil Nadu	415	168 (40.5)	282 (68.0)	288 (69.4)
Gulbarga	Karnataka	428	177 (41.4)	288 (67.3)	296 (69.2)
Simdega	Jharkhand	400	151 (37.8)	261 (65.3)	275 (68.8)
Gonda	Uttar Pradesh	457	232 (50.8)	293 (64.1)	314 (68.7)
Bankura	West Bengal	411	144 (35.0)	274 (66.7)	281 (68.4)
Kamareddy	Telangana	471	186 (39.5)	309 (65.6)	322 (68.4)
Chitradurga	Karnataka	451	193 (42.8)	293 (65.0)	307 (68.1)
Krishna	Andhra Pradesh	409	144 (35.2)	266 (65.0)	278 (68.0)
Jalandhar	Punjab	398	148 (37.2)	261 (65.6)	270 (67.8)
Purnia	Bihar	406	163 (40.1)	263 (64.8)	275 (67.7)
Gurdaspur	Punjab	390	144 (36.9)	247 (63.3)	261 (66.9)
Latehar	Jharkhand	422	180 (42.7)	239 (56.6)	279 (66.1)
Rayagada	Odisha	411	134 (32.6)	259 (63.0)	271 (65.9)
Koraput	Odisha	400	149 (37.3)	245 (61.3)	263 (65.8)
South Twenty Four Parganas	West Bengal	412	106 (25.7)	257 (62.4)	260 (63.1)
Pulwama	Jammu & Kashmir	430	124 (28.8)	263 (61.2)	271 (63.0)

District	State	Total Tested	N Positive (%)	S Positive (%)	N/S Positive (%)
Sri Potti Sriramulu Nellore	Andhra Pradesh	417	122 (29.3)	253 (60.7)	261 (62.6)
Parbhani	Maharashtra	420	130 (31.0)	252 (60.0)	262 (62.4)
Nanded	Maharashtra	403	141 (35.0)	242 (60.0)	251 (62.3)
Kullu	Himachal Pradesh	400	82 (20.5)	243 (60.8)	248 (62.0)
Alipurduar	West Bengal	408	83 (20.3)	241 (59.1)	247 (60.5)
Nalgonda	Telangana	451	171 (37.9)	252 (55.9)	273 (60.5)
Jangoan	Telangana	451	155 (34.4)	265 (58.8)	271 (60.1)
Kurukshetra	Haryana	398	137 (34.4)	218 (54.8)	239 (60.1)
Ahmadnagar	Maharashtra	419	131 (31.3)	243 (58.0)	251 (59.9)
Coimbatore	Tamil Nadu	428	172 (40.2)	244 (57.0)	252 (58.9)
Patiala	Punjab	399	147 (36.8)	210 (52.6)	233 (58.4)
Jalgaon	Maharashtra	414	155 (37.4)	211 (51.0)	241 (58.2)
Jyotiba Phule Nagar	Uttar Pradesh	400	152 (38.0)	205 (51.3)	230 (57.5)
Purba Medinipur	West Bengal	401	83 (20.7)	216 (53.9)	225 (56.1)
Jhargram	West Bengal	410	113 (27.6)	222 (54.1)	230 (56.1)
Kamrup Metropolitan	Assam	399	110 (27.6)	211 (52.9)	219 (54.9)
Sangli	Maharashtra	410	102 (24.9)	218 (53.2)	225 (54.9)
Udalguri	Assam	401	82 (20.4)	199 (49.6)	207 (51.6)
Bid	Maharashtra	402	126 (31.3)	169 (42.0)	202 (50.2)
Thrissur	Kerala	437	107 (24.5)	206 (47.1)	216 (49.4)
Pakur	Jharkhand	409	84 (20.5)	180 (44.0)	199 (48.7)
Karbi Anglong	Assam	403	93 (23.1)	162 (40.2)	179 (44.4)
Palakkad	Kerala	439	94 (21.4)	184 (41.9)	190 (43.3)
Ernakulam	Kerala	432	80 (18.5)	169 (39.1)	175 (40.5)

5. **Unweighted proportion of individuals with SARS-CoV-2 IgG antibodies among unvaccinated and aged above 10 years by districts, third and fourth national SARS-CoV-2 serosurvey, India**

State	District	General Population (Unvaccinated and >=10 Years) ROUND 3				General Population (Unvaccinated and >=10 Years) ROUND 4				Ratio of N/S
		Total Tested	N Positive (%)	S Positive (%)	N/S Positive (%)	Total Tested	N Positive (%)	S Positive (%)	N/S Positive (%)	
Gujarat	Mahisagar	407	11 (2.7)	15 (3.7)	20 (4.9)	171	84 (49.1)	108 (63.2)	113 (66.1)	13.5
Chhattisgarh	Surguja	396	33 (8.3)	31 (7.8)	43 (10.9)	250	126 (50.4)	186 (74.4)	199 (79.6)	7.3
Gujarat	Narmada	401	41 (10.2)	42 (10.5)	47 (11.7)	216	91 (42.1)	141 (65.3)	148 (68.5)	5.9
Uttar Pradesh	Saharanpur	396	41 (10.4)	51 (12.9)	60 (15.2)	294	167 (56.8)	192 (65.3)	211 (71.8)	4.7
Uttar Pradesh	Gonda	417	31 (7.4)	54 (12.9)	60 (14.4)	285	139 (48.8)	178 (62.5)	191 (67.0)	4.7
Madhya Pradesh	Dewas	390	51 (13.1)	58 (14.9)	73 (18.7)	274	160 (58.4)	209 (76.3)	219 (79.9)	4.3
Rajasthan	Rajsamand	404	58 (14.4)	57 (14.1)	69 (17.1)	265	148 (55.8)	179 (67.5)	192 (72.5)	4.2
Jharkhand	Latehar	398	37 (9.3)	48 (12.1)	60 (15.1)	256	117 (45.7)	133 (52.0)	163 (63.7)	4.2
Bihar	Begusarai	413	48 (11.6)	62 (15.0)	77 (18.6)	255	125 (49.0)	172 (67.5)	179 (70.2)	3.8
Uttar Pradesh	Balrampur	390	58 (14.9)	62 (15.9)	80 (20.5)	302	139 (46.0)	216 (71.5)	229 (75.8)	3.7
Uttarakhand	Garhwal	399	61 (15.3)	66 (16.5)	77 (19.3)	185	117 (63.2)	114 (61.6)	131 (70.8)	3.7
Uttar Pradesh	Unnao	401	44 (11.0)	66 (16.5)	76 (19.0)	250	125 (50.0)	156 (62.4)	171 (68.4)	3.6
Rajasthan	Dausa	390	49 (12.6)	62 (15.9)	75 (19.2)	249	132 (53.0)	146 (58.6)	166 (66.7)	3.5
Chhattisgarh	Kabeerdham	399	54 (13.5)	73 (18.3)	81 (20.3)	290	138 (47.6)	184 (63.4)	204 (70.3)	3.5
Gujarat	Sabar Kantha	399	62 (15.5)	75 (18.8)	83 (20.8)	220	96 (43.6)	145 (65.9)	155 (70.5)	3.4
Madhya Pradesh	Ujjain	405	53 (13.1)	78 (19.3)	84 (20.7)	271	155 (57.2)	173 (63.8)	188 (69.4)	3.4
Punjab	Jalandhar	398	55 (13.8)	66 (16.6)	77 (19.3)	245	85 (34.7)	152 (62.0)	158 (64.5)	3.3

State	District	General Population (Unvaccinated and >=10 Years) ROUND 3				General Population (Unvaccinated and >=10 Years) ROUND 4				Ratio of N/S
		Total Tested	N Positive (%)	S Positive (%)	N/S Positive (%)	Total Tested	N Positive (%)	S Positive (%)	N/S Positive (%)	
Haryana	Kurukshetra	397	45 (11.3)	60 (15.1)	66 (16.6)	243	78 (32.1)	110 (45.3)	127 (52.3)	3.2
Rajasthan	Jalor	409	75 (18.3)	74 (18.1)	93 (22.7)	195	104 (53.3)	123 (63.1)	134 (68.7)	3.0
Kerala	Thrissur	418	50 (12.0)	36 (8.6)	59 (14.1)	278	71 (25.5)	109 (39.2)	117 (42.1)	3.0
Bihar	Buxar	422	71 (16.8)	110 (26.1)	121 (28.7)	233	129 (55.4)	189 (81.1)	198 (85.0)	3.0
Kerala	Palakkad	408	46 (11.3)	55 (13.5)	58 (14.2)	325	72 (22.2)	130 (40.0)	136 (41.8)	2.9
Jharkhand	Simdega	400	59 (14.8)	68 (17.0)	78 (19.5)	193	70 (36.3)	101 (52.3)	110 (57.0)	2.9
Uttar Pradesh	Auraiya	398	61 (15.3)	86 (21.6)	99 (24.9)	304	186 (61.2)	188 (61.8)	219 (72.0)	2.9
Bihar	Muzaffarpur	401	41 (10.2)	87 (21.7)	93 (23.2)	275	141 (51.3)	169 (61.5)	184 (66.9)	2.9
Maharashtra	Parbhani	426	46 (10.8)	81 (19.0)	88 (20.7)	278	89 (32.0)	154 (55.4)	163 (58.6)	2.8
Karnataka	Chitradurga	442	67 (15.2)	96 (21.7)	103 (23.3)	276	133 (48.2)	171 (62.0)	182 (65.9)	2.8
Bihar	Arwal	416	63 (15.1)	109 (26.2)	118 (28.4)	299	147 (49.2)	223 (74.6)	233 (77.9)	2.7
Punjab	Ludhiana	399	56 (14.0)	89 (22.3)	101 (25.3)	271	113 (41.7)	182 (67.2)	187 (69.0)	2.7
Uttar Pradesh	Mau	417	72 (17.3)	89 (21.3)	108 (25.9)	319	172 (53.9)	214 (67.1)	223 (69.9)	2.7
Punjab	Patiala	400	59 (14.8)	75 (18.8)	84 (21.0)	272	107 (39.3)	132 (48.5)	153 (56.3)	2.7
Bihar	Madhubani	404	63 (15.6)	99 (24.5)	113 (28.0)	223	116 (52.0)	157 (70.4)	165 (74.0)	2.6
Madhya Pradesh	Gwalior	402	68 (16.9)	107 (26.6)	112 (27.9)	182	98 (53.8)	127 (69.8)	134 (73.6)	2.6
Uttar Pradesh	Bareilly	398	51 (12.8)	92 (23.1)	97 (24.4)	267	125 (46.8)	153 (57.3)	171 (64.0)	2.6
Bihar	Purnia	414	56 (13.5)	87 (21.0)	98 (23.7)	229	102 (44.5)	133 (58.1)	141 (61.6)	2.6
Punjab	Gurdaspur	399	75 (18.8)	81 (20.3)	101 (25.3)	241	89 (36.9)	148 (61.4)	158 (65.6)	2.6
Telangana	Kamareddy	413	51 (12.3)	102 (24.7)	106 (25.7)	314	126 (40.1)	187 (59.6)	199 (63.4)	2.5

State	District	General Population (Unvaccinated and >=10 Years) ROUND 3				General Population (Unvaccinated and >=10 Years) ROUND 4				Ratio of N/S
		Total Tested	N Positive (%)	S Positive (%)	N/S Positive (%)	Total Tested	N Positive (%)	S Positive (%)	N/S Positive (%)	
Uttar Pradesh	Jyotiba Phule Nagar	395	62 (15.7)	80 (20.3)	92 (23.3)	322	123 (38.2)	160 (49.7)	182 (56.5)	2.4
Jharkhand	Pakur	397	44 (11.1)	63 (15.9)	75 (18.9)	285	63 (22.1)	113 (39.6)	128 (44.9)	2.4
Karnataka	Gulbarga	429	47 (11.0)	113 (26.3)	117 (27.3)	268	118 (44.0)	161 (60.1)	168 (62.7)	2.3
Telangana	Nalgonda	420	59 (14.0)	96 (22.9)	108 (25.7)	327	132 (40.4)	170 (52.0)	189 (57.8)	2.2
Tamil Nadu	Coimbatore	411	69 (16.8)	91 (22.1)	100 (24.3)	289	117 (40.5)	149 (51.6)	156 (54.0)	2.2
Tamil Nadu	Tiruvannamalai	409	86 (21.0)	116 (28.4)	127 (31.1)	274	123 (44.9)	182 (66.4)	187 (68.2)	2.2
Maharashtra	Ahmadnagar	433	64 (14.8)	98 (22.6)	106 (24.5)	293	98 (33.4)	150 (51.2)	156 (53.2)	2.2
Kerala	Ernakulam	420	38 (9.0)	54 (12.9)	61 (14.5)	234	48 (20.5)	70 (29.9)	73 (31.2)	2.2
Uttar Pradesh	Gautam Buddha Nagar	398	77 (19.3)	109 (27.4)	126 (31.7)	284	130 (45.8)	175 (61.6)	189 (66.5)	2.1
Karnataka	Bangalore	418	81 (19.4)	126 (30.1)	142 (34.0)	284	116 (40.8)	193 (68.0)	201 (70.8)	2.1
West Bengal	Jhargram	419	89 (21.2)	110 (26.3)	119 (28.4)	347	105 (30.3)	194 (55.9)	202 (58.2)	2.0
Telangana	Jangoan	427	59 (13.8)	106 (24.8)	112 (26.2)	323	114 (35.3)	164 (50.8)	170 (52.6)	2.0
Maharashtra	Nanded	420	61 (14.5)	109 (26.0)	121 (28.8)	269	82 (30.5)	138 (51.3)	145 (53.9)	1.9
Andhra Pradesh	Sri Potti Sriramulu Nellore	405	96 (23.7)	121 (29.9)	127 (31.4)	242	76 (31.4)	134 (55.4)	140 (57.9)	1.8
West Bengal	Bankura	413	94 (22.8)	137 (33.2)	143 (34.6)	249	94 (37.8)	151 (60.6)	156 (62.7)	1.8
Andhra Pradesh	Vizianagaram	410	57 (13.9)	166 (40.5)	172 (42.0)	256	130 (50.8)	188 (73.4)	194 (75.8)	1.8
Odisha	Koraput	401	96 (23.9)	124 (30.9)	140 (34.9)	262	106 (40.5)	142 (54.2)	160 (61.1)	1.8
Maharashtra	Jalgaon	427	67 (15.7)	121 (28.3)	135 (31.6)	297	115 (38.7)	138 (46.5)	162 (54.5)	1.7
Maharashtra	Bid	421	57 (13.5)	98 (23.3)	106 (25.2)	264	85 (32.2)	88 (33.3)	114 (43.2)	1.7
Tamil Nadu	Chennai	408	72 (17.6)	166 (40.7)	175 (42.9)	240	111 (46.3)	167 (69.6)	174 (72.5)	1.7

State	District	General Population (Unvaccinated and >=10 Years) ROUND 3				General Population (Unvaccinated and >=10 Years) ROUND 4				Ratio of N/S
		Total Tested	N Positive (%)	S Positive (%)	N/S Positive (%)	Total Tested	N Positive (%)	S Positive (%)	N/S Positive (%)	
Andhra Pradesh	Krishna	403	70 (17.4)	148 (36.7)	154 (38.2)	238	90 (37.8)	138 (58.0)	147 (61.8)	1.6
Jammu & Kashmir	Pulwama	419	99 (23.6)	132 (31.5)	145 (34.6)	198	61 (30.8)	104 (52.5)	110 (55.6)	1.6
Chhattisgarh	Bijapur	396	134 (33.8)	150 (37.9)	176 (44.4)	274	109 (39.8)	186 (67.9)	195 (71.2)	1.6
Assam	Karbi Anglong	413	71 (17.2)	86 (20.8)	102 (24.7)	294	77 (26.2)	98 (33.3)	115 (39.1)	1.6
Odisha	Ganjam	408	95 (23.3)	142 (34.8)	157 (38.5)	205	86 (42.0)	116 (56.6)	122 (59.5)	1.5
West Bengal	Purba Medinipur	415	79 (19.0)	124 (29.9)	133 (32.0)	282	61 (21.6)	130 (46.1)	136 (48.2)	1.5
Odisha	Rayagada	407	87 (21.4)	154 (37.8)	166 (40.8)	277	98 (35.4)	149 (53.8)	161 (58.1)	1.4
West Bengal	South 24 Parganas	414	103 (24.9)	150 (36.2)	167 (40.3)	256	68 (26.6)	138 (53.9)	140 (54.7)	1.4
Assam	Kamrup Metropolitan	408	89 (21.8)	134 (32.8)	150 (36.8)	259	77 (29.7)	118 (45.6)	125 (48.3)	1.3
West Bengal	Alipurduar	415	90 (21.7)	163 (39.3)	175 (42.2)	310	64 (20.6)	164 (52.9)	170 (54.8)	1.3
Himachal Pradesh	Kullu	400	153 (38.3)	141 (35.3)	166 (41.5)	186	46 (24.7)	94 (50.5)	99 (53.2)	1.3
Assam	Udalguri	407	90 (22.1)	114 (28.0)	142 (34.9)	271	55 (20.3)	112 (41.3)	119 (43.9)	1.3
Maharashtra	Sangli	426	88 (20.7)	156 (36.6)	161 (37.8)	243	61 (25.1)	100 (41.2)	106 (43.6)	1.2

6. Seroprevalence of selected characteristics by vaccination status, Healthcare workers, June – July 2021

Characteristics	Unvaccinated			Vaccinated with one dose			Vaccinated with two doses		
	Tested	Positives (N/S)	Cluster and test adjusted Seroprevalence % (95% CI)	Tested	Positives (N/S)	Cluster and test adjusted Seroprevalence % (95% CI)	Tested	Positives (N/S)	Cluster and test adjusted Seroprevalence % (95% CI)
**Age (years)**									
18-44	651	429	65.6 (60.5 - 70.5)	828	712	88.2 (84.8 - 91.0)	3654	3260	90.1 (88.5 - 91.5)
45-60	105	77	73.8 (63.3 - 82.0)	142	120	85.1 (77.7 - 90.5)	1750	1489	86.1 (83.7 - 88.2)
>60	3	1	35.6 (3.8 - 87.4)	2	2	-	117	96	81.9 (73.2 - 88.2)
**Sex**									
Male	343	230	66.0 (59.7 - 72.0)	451	392	88.3 (84.4 - 91.5)	2729	2402	88.7 (86.9 - 90.4)
Female	414	277	67.4 (61.4 - 72.9)	521	442	87.1 (83.0 - 90.4)	2787	2438	88.5 (86.7 - 90.3)
Other	2	0	-	-	-	-	5	5	-
**Results of COVID-19 testing**									
Reported positive for COVID-19	140	116	85.0 (77.0 - 90.6)	154	146	95.6 (90.9 - 97.9)	1060	1013	96.3 (94.9 - 97.3)
Reported Negative for COVID-19	267	156	58.7 (50.5 - 66.4)	491	408	85.9 (80.9 - 89.7)	2637	2225	85.4 (83.1 - 87.4)
Don't know	16	11	71.9 (37.0 - 91.7)	15	15	-	112	104	92.1 (81.9 - 96.8)
**Vaccine type**									
Covaxin	-	-	-	127	96	79.0 (69.5 - 86.0)	371	332	89.0 (84.8 - 92.2)
Covishield	-	-	-	841	734	88.5 (85.5 - 91.1)	5132	4495	88.6 (87.0 - 90.1)
**Optimal interval between vaccination and blood sample collection*** (21 days after 1st dose, 7 days after 2nd dose)									
Covaxin	-	-	-	103	81	83.8 (73.5 - 90.6)	368	329	88.9 (84.7 - 92.1)
Covishield	-	-	-	675	599	91.0 (87.4 - 93.6)	5127	4490	88.6 (87.0 - 90.1)

7. Seroprevalence of IgG antibodies against Nucleocapsid by selected characteristics, June-July 2021

Characteristics	General population			Healthcare workers		
	No. tested	No. positive (anti-N antibodies)	Weighted and test-performance-adjusted seroprevalence % (95% CI)	No. tested	No. positive (anti-N antibodies)	Test-performance-adjusted seroprevalence % (95% CI)
**Age (years)**						
6-9	2892	1076	36.5 (34.4 - 38.8)	-	-	-
10-17	5798	2329	39.0 (37.2 - 40.7)	-	-	-
18-44	12522	4847	37.7 (36.2 - 39.1)	5133	1635	31.0 (28.7 - 33.4)
45-60	5545	2149	38.3 (36.4 - 40.0)	1997	624	30.2 (27.5 - 33.1)
>60	2218	888	41.5 (39.1 - 44.0)	122	46	36.5 (28.1 - 46.0)
**Sex**						
Male	13783	5311	37.1 (35.8 - 38.6)	3523	1162	31.5 (29.0 - 34.0)
Female	15160	5967	39.2 (37.9 - 40.6)	3722	1141	30.3 (27.9 - 32.8)
Other	32	11	16.4 (6.1 - 36.3)	7	2	26.6 (6.2 - 65.7)
**Area of residence**						
Rural	21794	8556	39.0 (37.6 - 40.4)	-	-	-
Urban non-slum	5266	1997	37.2 (34.7 - 39.8)	-	-	-
Urban slum	1915	736	37.3 (33.1 - 41.6)	-	-	-
**History of COVID-19 related symptoms since 1 Jan 2021**						
Yes	1748	808	51.6 (48.7 - 54.6)	925	422	45.9 (41.8 - 50.1)
No	27227	10481	37.6 (36.3 - 38.8)	6327	1883	28.7 (26.5 - 31.0)
**Results of COVID-19 testing**						
Reported positive for COVID-19	782	464	63.4 (59.8 - 66.7)	1354	630	46.6 (42.9 - 50.3)
Reported Negative for COVID-19	3419	1222	38.1 (35.8 - 40.3)	3395	922	26.3 (24.0 - 28.9)
Don't know	171	55	34.5 (24.4 - 46.3)	143	56	37.4 (26.6 - 49.8)
**COVID-19 vaccination among adults**						
0 dose	12599	5122	40.3 (38.9 - 41.7)	759	339	43.2 (39.0 - 47.5)
1 dose	5038	1898	36.1 (34.3 - 38.0)	972	358	35.9 (32.2 - 39.8)
2 doses	2631	856	31.3 (29.1 - 33.5)	5521	1608	28.3 (26.2 - 30.5)
**Vaccine type**						
Covaxin	587	274	50.6 (45.7 - 55.5)	498	255	51.6 (46.5 - 56.8)
Covishield	6945	2422	32.7 (30.9 - 34.5)	5973	1702	27.6(25.5 - 29.8)

8. Estimated number of infections among individuals aged 6 years above and infection case ratio

	**Estimate (95% CI) by N/S - Seroprevalence considering the unvaccinated population***	**Estimate (95% CI) by N/S - Seroprevalence by considering the entire population****
Estimated number of infections	64,27,51,546 (62,59,01,252 – 65,96,01,839)	80,73,95,611 (78,64,69,887 – 82,83,21,335)
Number of reported COVID-19 cases (9 June 2021)	2,90,88,245	2,90,88,245
**Infection Case ratio (9 June 2021)**	22.1 (21.5 - 22.7)	27.8 (27.0 - 28.5)
Number of reported COVID-19 cases (16 June 2021)	2,96,32,302	2,96,32,302
**Infection Case ratio (16 June 2021)**	21.7 (21.1 - 22.3)	27.2 (26.5 - 28.0)

* Applying the weighted seroprevalence to the population aged 6 -17 years and the unweighted seroprevalence among unvaccinated individuals aged 18 years and above to the total population of unvaccinated individuals aged >=18 years
** Applying the weighted seroprevalence to the population aged 6 -17 years and the weighted seroprevalence among unvaccinated individuals aged 18 years and above to the total population aged >=18 years

9. **Comparison of seroprevalence against N/S-antibody by demographic characteristics between third and fourth serosurvey**

	Third serosurvey Prevalence (95%CI)	Fourth serosurvey Prevalence (95%CI)
**Age in years**		
10-17	27.2 (24.9 - 29.4)	61.6 (59.8 - 63.3)
18-44	22.2 (21.1 - 23.4)	66.7 (65.3 - 68.0)
45-60	26.7 (25.2 - 28.2)	77.6 (76.1 - 79.0)
>60	26.3 (24.3 - 28.3)	76.7 (74.6 - 78.7)
**Sex**		
Male	23.2 (22.1 - 24.5)	65.8 (64.4 - 67.1)
Female	24.9 (23.7 - 26.3)	69.2 (67.9 - 70.5)
Others	---	
**Residence**		
Rural	21.4 (20.3 - 22.6)	66.7 (65.4 - 68.1)
Urban non-slum	29.5 (27.0 - 32.1)	69.1 (66.6 - 71.6)
Urban Slum	34.7 (31.2 - 38.5)	71.0 (66.8 - 74.7)
