# Supplementary material for: Seroprevalence of IgG antibodies against SARS-CoV-2 among the general population and healthcare workers in India, June–July 2021: A population-based cross-sectional study
Source: PLoS Med. 2021 Dec 10;18(12):e1003877. doi: 10.1371/journal.pmed.1003877 (PMC8726494; doi:10.1371/journal.pmed.1003877)
Supplement: Attachment — Submitted filename: murhekar.pdf [file pmed.1003877.s013.pdf]

This paper describes a survey of the prevalence of antibodies against SARS–CoV–2 in India.

I was asked for a statistical report and I interpret that to include all aspects of the design and conduct of the study.

Points of detail

Page 3 '... in the same 70 districts...'. The same as what? Page 4 is much better as it tells us so I would re–word here.

Page 4 India may have the second highest number of cases but it is a big country. Cases per million would be much more informative.

Page 4 How were the individual participants sampled in each district? Is this a random sample from an enumerated list? Are these people the same as those in previous waves or are they chosen to be different or some mixture of the two? Page 2 of the supplement does not expand on this.

Page 5 What does it mean to sample HCW consecutively? Do the authors mean they sampled people in the order they arrived for work in the morning? Are these people consecutively mentioned in the payroll? Are they different or the same as HCW in previous waves.

Page 6 I am afraid I do not understand the two scenarios for estimating total number of infections nor why two methods were needed when on the face of it they seem very different. Indeed Supplementary 8 suggests they do give rise to very different estimates.

Page 9 I do not see any mention in the results section of genomic scanning in this study so this is going beyond the authors' study. Similarly I see no mention of mask wearing. I suggest these should be removed. The same applies to the abstract.

Page 18 We do not seem to have equivalent figures for the HCW.

Table 1 Do the authors mean sex rather than gender? I am no expert here but my understanding is that some cultures recognise more than two genders and I believe in India there are *hijra*.

Table 4 I think it might be helpful for the caption to tell us what the abbreviation N/S means. I know if the reader has looked at the previous tables it should be fairly clear. The same applies to some of the supplementary tables.

Supplementary 8 This uses the *crore* and *lakh* notation which I do not think is widely understood outside South Asia so I would change to the more widely accepted millions and thousands notation.

Points of more substance

We need much more detail about how participants were selected in each cluster. Since about 18% of those approached did not participate we need to know which characteristics predict missingness and the models need to include those characteristics. In general the authors would benefit from re–visiting the STROBE guidelines (von Elm et al., 2007; Vandenbroucke et al., 2007) and provide more information as requested there.

Summary

Requests for clarification and more details.

Michael Dewey

References

J P Vandenbroucke, E von Elm, D G Altman, P C Gøtzsche, C D Mulrow, S J Pocock, C Poole, J J Schlesselman, M Egger, and the STROBE initiative. Strengthening the reporting of observational studies in epidemiology (STROBE): explanation and elaboration. *PLOS Medicine*, 4(e297), 2007.

E von Elm, M Egger, D G Altman, S J Pocock, P C Gøtzsche, J P Vandenbroucke, and for the STROBE Initiative. Strengthening the reporting of observational studies in epidemiology (STROBE) statement: guidelines for reporting observational studies. *British Medical Journal*, 335:806–808, 2007.
