# Supplementary material for: Seroprevalence of IgG antibodies against SARS-CoV-2 among the general population and healthcare workers in India, June–July 2021: A population-based cross-sectional study
Source: PLoS Med. 2021 Dec 10;18(12):e1003877. doi: 10.1371/journal.pmed.1003877 (PMC8726494; doi:10.1371/journal.pmed.1003877)
Supplement: Attachment — Submitted filename: Replies to reviewers comments_MM_18Oct_F.docx [file pmed.1003877.s014.docx]

**Replies to reviewers comments**

Requests from the editors:

1. We ask you to limit the number of authors to fewer than 30, with the additional contributors forming an author group.

>>>>>>We request the Editor to kindly consider our request to include all the authors in the main list as they meet the ICMJE criteria for authorship. Earlier publications of the three national serosurveys in India had included all the authors in the main list. However, if only 30 authors can be included as per the journal guidelines in the main list, we will include the remaining authors as group, with a request to kindly index all the authors in the PubMed.

2. Noting PLOS' data policy (https://journals.plos.org/plosmedicine/s/data-availability), please confirm that de-identified study data will be made available, and supply a non-author contact for those interested in inquiring about data access.

The authors confirm that, for IHEC-approved reasons, some access restrictions apply to the data underlying the findings. An individual may email to ICMR Data Repository office ([harpreets.hq@icmr.gov.in](mailto:harpreets.hq@icmr.gov.in)) to request the data. Given the nature of these data, we will ask users to sign a data sharing agreement. This is not intended to restrict access, but to ensure that requests are for ethical research purposes and that any analyses undertaken will not compromise the confidentiality of individual participants, and are not for commercial purposes.

3. Please adapt the title to include a study descriptor following a colon, e.g., "... June-July, 2021: A population-based cohort study".

>>>We have changed the title to “Seroprevalence of IgG antibodies against SARS-CoV-2 among the general population and healthcare workers in India, June–July 2021: A population-based cross-sectional study”

4. In the abstract and throughout the paper, please quote p values alongside 95% CI, where available.

>>>We have quoted the p values alongside 95% CI, wherever the seroprevalence is compared between the subgroups.

5. Please trim the "Conclusions" subsection of your abstract to around half the current length.

>>>As suggested, we have trimmed the conclusion sub-section of the manuscript.

6. After the abstract, please add a new and accessible "Author summary" in non-identical prose. You may find it helpful to consult one or two recent research papers published in PLOS Medicine to get a sense of the preferred style.

>>>We have added Author Summary in the abstract.

7. In the methods section, please state whether the study had a protocol or prespecified analysis plan, and if so attach the document as a supplementary file, referred to in the text.

>>>The study has a protocol and we have attached the same as supplementary file

8. Please highlight analyses that were not prespecified.

>>>Sensitivity analysis for the range of sero-prevalence was not pre-specified in the protocol.

9. Throughout the text, please adapt reference call-outs to the following style: "... cases reported globally [1].".

>>>We have made the changes as suggested by the Editor.

10. Please remove the information on competing interests and data sharing from the end of the main text. In the event of publication this will appear in the article metadata, via entries in the submission form.

>>>We have removed the information on competing interests and data sharing from the end of the main text.

11. Please move the list of contributors at the end of the ms to a supplementary file.

>>>We have moved the list of contributors at the end of manuscript to supplementary file

12. In the reference list, please convert italics and boldface text into plain text.

>>>We have made the changes to the reference list.

13. Where appropriate, 6 author names should be listed, followed by "et al.".

    >>>We have made the changes to the reference list.
14. Please add a completed checklist for the most appropriate reporting guideline, e.g., STROBE, as an attachment, labelled "S1_STROBE_Checklist" or similar and referred to as such in your Methods section. In the checklist, please refer to individual items by section, e.g., "Methods", and paragraph numbers, not by line or page numbers as these generally change in the event of publication.

>>>We have included STROBE checklist for cross-sectional studies.

**Comments from the reviewers:**

**Reviewer #1:**

This work is one of the extensive work done during the time of covid-19 and particularly from countries where drastic infection has occurred. Very limited layout or format comments.

1. I like the title of "Seroprevalance" in this case than "prevalence", as testing was used to detect the antibodies rather than other type of testing.

>>>We agree with the reviewer’s suggestion and have changed the titled as: “Seroprevalence of IgG antibodies against SARS-CoV-2 among the general population and healthcare workers in India, June–July 2021: A population-based cross-sectional study”

2. Abstract: the paragraph related to the limitations, could be reflected clearly under subheadings in the discussion, as it was done. no need to be in the abstract.

>>>The submission guidelines recommend describing the main limitations of the study in the abstract under Methods and Findings. We have therefore included a line about the limitations of the study.

3. Abstract, in the conclusion: the second line, "by June 2020". I think it should be stated as "June 2021".

>>>We thank the reviewer for pointing out this mistake. We have corrected the same in the revised manuscript.

4. Abstract: The conclusion is so long and should be reduced to the main points. No matter to be little wider in the text.

>>>We have shortened the conclusion section.

5. Result, page 7: The paragraph started with "the weighted prevalence of IgG antibodies ...........(table 2)", probably should be stated when the description of table 2 is done.

>>>We have made the changes as suggested by the reviewer.

6. References, page 11: Format and layout of the references is required according to the Journal instructions.

>>>We have updated the reference style in the revised manuscript, as per the journal instructions.

7. I recommend to use the table related to the Health care workers to be among the main tables in the text and not among the supplementary.

>>>As suggested by the reviewer, we have included the table showing seroprevalence among healthcare workers by selected characteristics (supplementary table 6) in the main table.

**Reviewer #2:**

1. Abstract: in conclusion there is typo error ".... by June 2020"

>>>We have corrected the typographical error.

2. Methods: sample size calculation with design effect should be provided for the general population and also for the HCWs.

>>>We have mentioned the sample size calculation in the supplementary material of the revised manuscript.

**Reviewer #4:**

1. Important study showing the power of infection and capacity of transmission this virus in a different population (non-HCW and HCW) living in different regions (rural, non-slum, and -slum areas) of a big country. The authors dedicate strong space to describe the methods used, however, I would like to suggest improving the description of the regression model, including why the authors did not use Poisson Regression model in this cross-sectional study?!

>>>We have provided the details of regression model in the S3_Text_Data analysis. Since the presence of IgG antibodies on account of SARS-CoV-2 infection as well as COVID-19 vaccination is not a rare event, we did not consider Poisson regression model. The random effects model was considered appropriate to account for heterogeneity in the seroprevalence within the selected clusters (villages/wards) of the districts. We have added these details in the Supplementary material.

2. On the other hand, I understood the use of weights design mainly because of the probable difference among the size population of the district studied, however, the authors should have provided in the tables (#3, and #4 and #5) the target-population, this is important to share with the readers the probable high variability among the clusters represented of the districts studied.

>>>As suggested by the reviewer, we have provided the target population of the selected districts and clusters selected within each district in the S6_Table. We feel that presenting the total size of the target population in table 3,4 and 5 might not reflect the variability among the clusters selected within the districts.

**Reviewer #5 (Michael Dewey):**

1. Page 3 ‘... in the same 70 districts...’. The same as what? Page 4 is much better as it tells us so I would re–word here.

>>>We have revised the text to “same 70 districts where the earlier three serosurveys were conducted”.

2. Page 4 India may have the second highest number of cases but it is a big country. Cases per million would be much more informative.

>>>As suggested by the reviewer, we have provided information about cases per million besides providing total number of cases.

3. Page 4 How were the individual participants sampled in each district? Is this a random sample from an enumerated list? Are these people the same as those in previous waves or are they chosen to be different or some mixture of the two? Page 2 of the supplement does not expand on this.

>>>From each district, we selected 10 clusters (Wards in urban areas and villages in rural areas) from each district using population proportion to size method. Since the sampling frame of the selected district was not available, and since the enumeration of all households in the 700 clusters was not feasible, the survey teams selected four random starting points within each of the selected cluster. Starting from a random starting point, the teams visited consecutive households and listed all household members aged 6 years and above and who were permanently residing in the area. From each random point, at least 10 consenting individuals (1 aged 6-9 years, 2 aged 10-17 years and 7 aged 18 years and above) were enrolled in the survey.

>>>Since the clusters selected in the fourth round of survey were different than the earlier rounds, survey participants were different than the earlier surveys.

This information is provided in the methods section of the revised manuscript as well as in S2_Study protocol.

4. Page 5 What does it mean to sample HCW consecutively? Do the authors mean they sampled people in the order they arrived for work in the morning? Are these people consecutively mentioned in the payroll? Are they different or the same as HCW in previous waves.

>>>We enrolled all the willing HCWs working in the selected district health facilities. In case, the required sample size of 100 was not achieved, the survey team visited the sub-district level health facility nearest to the district health facility and all will HCWs were enrolled in the study. We have deleted the word consecutively in the revised manuscript.

>>>The third serosurvey was conducted among HCWs working in the sub-district level health facilities, whereas in the present round HCWs working in the district level health facilities were surveyed.

5. Page 6 I am afraid I do not understand the two scenarios for estimating total number of infections nor why two methods were needed when on the face of it they seem very different. Indeed Supplementary 8 suggests they do give rise to very different estimates.

>>>In the first three serosurveys, we applied the weighted seroprevalence to the entire population to estimate the number of individuals infected with SARS-CoV-2 for calculation of infection to case ratio. However, since the COVID-19 vaccination has begun in India, the presence of IgG antibodies could be on account of natural infection or COVID-19 vaccination. We therefore considered two scenarios: First, we applied the weighted seroprevalence among unvaccinated individuals aged >=18 years to the total population of unvaccinated individuals aged >=18 years. Second, we applied the weighted seroprevalence among individuals aged >=18 years to the total population above 18 years (vaccinated as well as unvaccinated). The estimated number of infection and hence ICR are expected to be more conservative in the first method as 24% of India’s population had received at least one dose of COVID-19 vaccines by 16 June 2021.

6. Page 9 I do not see any mention in the results section of genomic scanning in this study so this is going beyond the authors’ study. Similarly I see no mention of mask wearing. I suggest these should be removed. The same applies to the abstract.

>>> We agree with the reviewer’s point. However, we have mentioned about the need for strengthening genomic surveillance and continued use of non-pharmaceutical interventions including the use of face mask, in the context of discussion on the implications of serosurvey findings. We feel that these measures are required to reduce the transmission as well detect any upsurge of cases on account of vaccine escape variants. We are therefore keeping the text in the discussion but have removed the same from the abstract.

7. Page 18 We do not seem to have equivalent figures for the HCW.

>>>We have included equivalent figure for HCWs showing number of HCWs who refused and who participated in the survey.

8. Table 1 Do the authors mean sex rather than gender? I am no expert here but my understanding is that some cultures recognise more than two genders and I believe in India there are hijra.

>>>We thank the reviewer for this comment. We have replaced the word ‘sex’ with ‘gender’ in table-1 and throughout the manuscript.

9. Table 4 I think it might be helpful for the caption to tell us what the abbreviation N/S means. I know if the reader has looked at the previous tables it should be fairly clear. The same applies to some of the supplementary tables.

>>>We have revised the abbreviations (N/S) in table 4 as well as supplementary tables.

10. Supplementary 8 This uses the crore and lakh notation which I do not think is widely understood outside South Asia so I would change to the more widely accepted millions and thousands notation.

>>>We agree with reviewer’s suggestions. We have changed the notations to millions.

11. Points of more substance : We need much more detail about how participants were selected in each cluster.

>>>We have provided the details of participant selections within each cluster in the methods section of revised manuscript as well as S2_Study protocol.

12. Since about 18% of those approached did not participate we need to know which characteristics predict missingness and the models need to include those characteristics. In general the authors would benefit from revisiting the STROBE guidelines (von Elm et al., 2007; Vandenbroucke et al., 2007) and provide more information as requested there.

>>>In the revised manuscript and S10_table, we have shown the comparison of characteristics (age/sex) of individuals who participated and who did not participate in the survey and have mentioned its implications on the seroprevalence in the limitation section of revised manuscript.
