# Supplementary material for: Seroprevalence of IgG antibodies against SARS-CoV-2 among the general population and healthcare workers in India, June–July 2021: A population-based cross-sectional study
Source: PLoS Med. 2021 Dec 10;18(12):e1003877. doi: 10.1371/journal.pmed.1003877 (PMC8726494; doi:10.1371/journal.pmed.1003877)
Supplement: Attachment — Submitted filename: PMEDICINE_Reply to Reviewers comments_Final_TJ.docx [file pmed.1003877.s015.docx]

**Reply to reviewers’ comments**

1. Please adapt the data sharing statement (submission form) to read "... Given the nature of the data, potential users will be asked to sign a data-sharing agreement".

>>>We have revised the data sharing statement as suggested by the editor in the submission form.

2. As mentioned previously, please include no more than 30 authors, with the remaining individuals participating as a named author group, with group members listed in the Acknowledgements.

>>> We have included 30 authors and the rest have been listed as a named group author. We request you that the group authors may be placed above the acknowledgement section and if possible index all the authors in the PubMed.

3. We ask you to add an additional sentence, say, to the abstract to quote additional information on the cohort studied (quoting the number of participants and some information on age, sex and residence).

>>> We have added information regarding number of participants along with their age and gender in the abstract of the revised manuscript

4. Please make that "test-adjusted" in the abstract, and throughout the text where the phrase is used as an adjective.

>>> We have revised phrase as test-adjusted both in abstract and in main text of the revised manuscript

5. Please revisit the "Author summary" section, aiming for no more than 3-4 points in each of the three subsections. For example, the final point of the first subsection ("We conducted the fourth ...") should either be deleted or moved to the second subsection.

>>> We have revised the author summary as per suggestions given by the editor

6. At the end of the Introduction (main text), please make that "We conducted the fourth round of a national ...".

>>> We have made the changes in the revised manuscript

7. Under editorial query "8" you mention non-prespecified sensitivity analyses. Please add a few words to the methods and/or results sections of the paper, if not already present, to highlight these non-prespecified analyses.

>>> We have already mentioned in the methods section about the non-prespecified sensitivity analyses.

8. In the second paragraph of the Discussion section (main text) there is a superscript "2". Please reformat this if it is a reference call-out.

>>> We have made the changes in the revised manuscript

9. Noting reviewer 5's comment, we ask you to add "we believe" or similar when you are making recommendations not directly based on the findings of your study.

>>> We have reworded the recommendation as suggested by the reviewer

10. Please spell out the institutional author name for reference 10.

>>> We have mentioned the author name for reference 10 in the revised manuscript

11. Noting references 15 & 16, please add "[preprint]" to all preprints cited, unless you are able to substitute the corresponding peer-reviewed papers.

>>> We have mentioned the preprint to all the reference that are in preprints stage and replaced with corresponding peer-reviewed papers wherever it was possible

*** Reviewer #4:

12. I believe is very important to discuss in the limitation section the option related to use in the analysis a mixed-models GLM with overdispersion of the data, mainly when it's applied in cross sectional study.

>>> The overall mean and standard deviation of the seroprevalence was given by 0.667 (0.471). This shows that the observed mean was greater than variance which rules out the condition of overdispersion. Of the cluster specific mean and standard deviation of seropositivity, lowest estimate was 0.175 (0.384) and highest estimate was 0.973 (0.164). This shows a few clusters to have overdispersion. Nearly 12% (83/700) of the clusters have been found to be over dispersed. The heterogeneity seen might not affect the overall estimates and mixed effects models would have taken care of the over-dispersion seen in a few clusters.

*** Reviewer #5:

13. The authors have addressed my points. We still disagree about the wisdom of including recommendations (genomic scanning and mask wearing) in the conclusion which are not supported by the authors' research however sensible they may be. I think this is perhaps more of an editorial policy issue so I leave it to the team to decide.

>>> We have reworded the recommendation as suggested by the editor.
